# Acyl Amidines by Pd-Catalyzed Aminocarbonylation: One-Pot Cyclizations and ^11^C Labeling

**DOI:** 10.1021/acs.joc.2c02115

**Published:** 2022-12-15

**Authors:** Jonas Rydfjord, Sara Roslin, Tamal Roy, Alaa Abbas, Marc Y. Stevens, Luke R. Odell

**Affiliations:** Department of Medicinal Chemistry, Uppsala University, Box 574, SE-751 23 Uppsala, Sweden

## Abstract

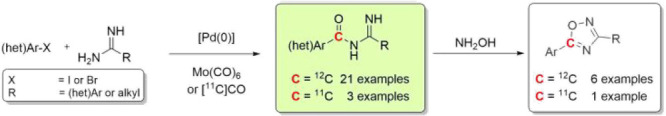

A protocol for the carbonylative synthesis of acyl amidines from aryl halides, amidines, and carbon monoxide catalyzed by Pd(0) is reported herein. Notably, carbon monoxide is generated ex situ from a solid CO source, and several productive palladium ligands were identified with complementary benefits and substrate scope. Furthermore, sequential one-pot, two-step protocols for the synthesis of 1,2,4-triazoles and 1,2,4-oxadiazoles via acyl amidine intermediates are reported. In addition, this approach was extended to isotopic labeling using [^11^C]carbon monoxide to allow, for the first time, synthesis of ^11^C-labeled acyl amidines as well as a ^11^C-labeled 1,2,4-oxadiazole.

## Introduction

Acyl amidines are useful intermediates in the synthesis of a number of heterocycles, such as 1,2,4-triazoles,^1,2^ 1,3,5-triazines,^3^ 1,2-dihydro-3*H*-pyrrol-3-ones,^4^ and 1,2,4-oxadiazoles.^5,6^ They are also interesting motifs in drug discovery, and biologically active examples are found throughout the literature, including angiotensin II receptor ligands,^7^ thrombin (prodrug),^8^ β-secretase,^9^ cathepsin D,^9^ and renin inhibitors.^9^ The most straightforward synthesis of acyl amidines is by acylation of amidines and was indeed reported by Pinner already in 1889 from an acid anhydride.^10^ Alternative strategies include reaction of acylimidates with amines,^11,12^ hydroalumination,^13^ a copper-catalyzed multicomponent reaction,^14^ and a rhodium-catalyzed synthesis from nitrosobenzene derivatives with *N*-sulfonyl-1,2,3-triazoles.^15^

**Scheme 1:**
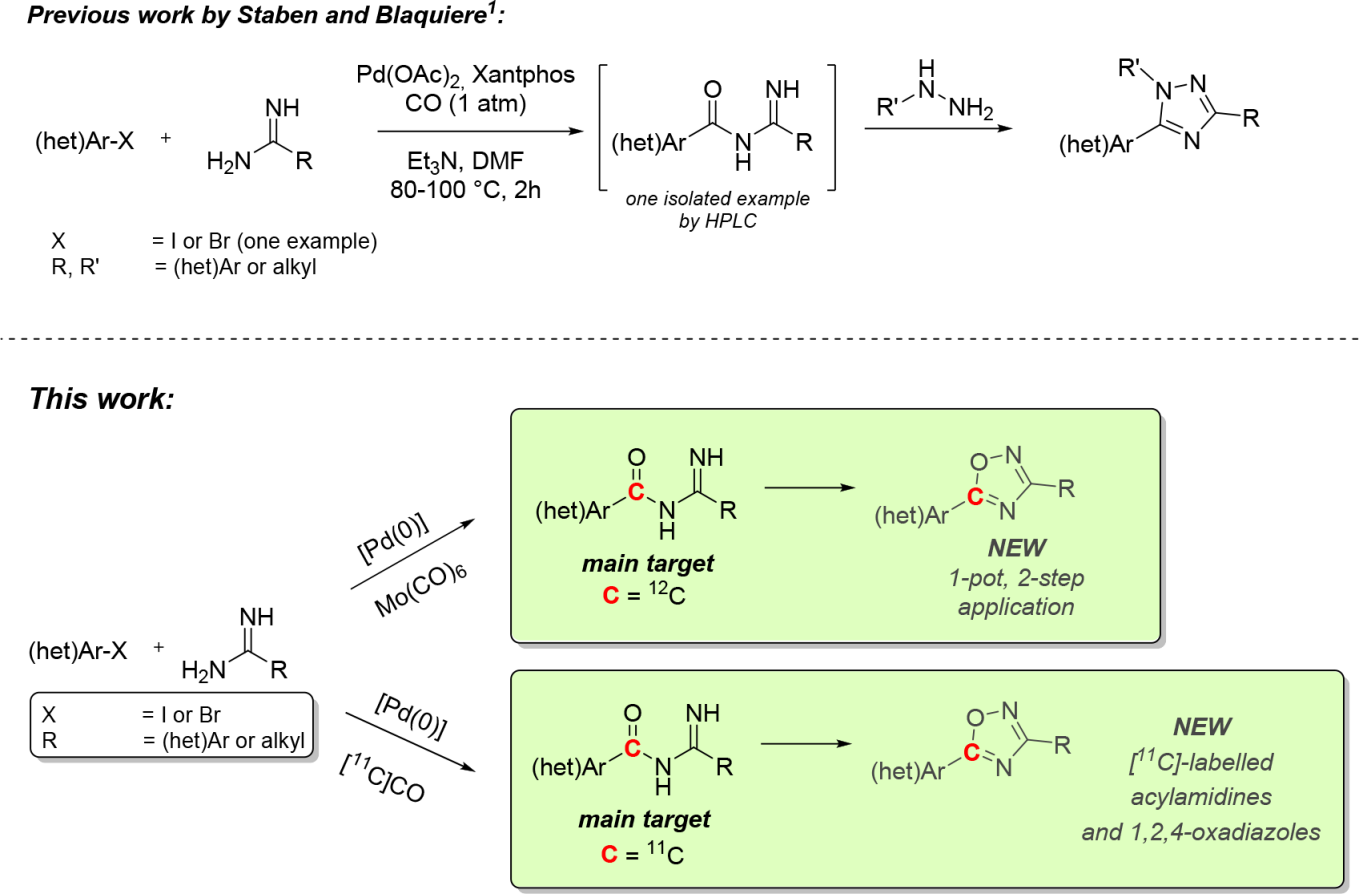
Previous Work by Staben and Blaquiere and the Work Presented Herein

As part of our research program on the development of palladium(0)-catalyzed carbonylation reactions, we have previously investigated the use of amidine nucleophiles to afford acyl amidines. Initial attempts using molybdenum hexacarbonyl as an in situ solid CO source^16^ were unsuccessful due to problematic purification of the product, and the project was halted. Since then, Staben and Blaquiere have published an elegant one-pot, two-step protocol in which they used aryl iodides (and one example of an aryl bromide), amidines, and carbon monoxide in a palladium(0)-catalyzed carbonylation to give acyl amidines, which were subsequently reacted with hydrazines to give the corresponding 1,2,4-triazoles (see Scheme 1). More recently, two-chamber systems such as COware developed by Skrydstrup et al. have enabled the use of ex situ carbon monoxide generated by a large array of convenient carbon monoxide sources.^17,18^ With this progress in mind, we decided to re-evaluate the carbonylative synthesis of acyl amidines, this time taking advantage of a two-chamber system^19^ for ex situ generation of carbon monoxide from Mo(CO)_6_. Noting that the acyl amidine was only isolated as one example by HPLC in the protocol by Staben and Blaquiere, we decided to focus on developing a method for the synthesis and isolation of acyl amidines using a safe and convenient solid source of carbon monoxide.^1^ As secondary objectives, we noted that heterocycles other than 1,2,4-triazoles should be accessible in a similar one-pot, two-step fashion and thus decided to pursue a protocol for the synthesis of 1,2,4-oxadiazoles, a structural motif present in many biologically active compounds as well as in approved drugs.^20^

One of the major advantages of the carbonylation reaction, in comparison with other strategies to access carbonyl derivatives, is the ability to prepare ^11^C-, ^13^C-, or ^14^C-labeled products using isotopically modified carbon monoxide. To demonstrate this versatility, the method was also translated into a radiochemical setting to produce ^11^C-labeled acyl amidines and 1,2,4-oxadiazoles by employing [^11^C]CO, thus enabling future positron emission tomography (PET) applications.

## Results and Discussion

The investigation started by screening solvents, catalysts, stoichiometry, time, and temperature to establish general reaction conditions for the reaction between aryl iodides and amidines, see Table 1. 4-Iodotoluene (**1a**) and benzamidine (**2a**) were chosen as model substrates, and the reaction was performed in a two-chamber setup (see SI) in which chamber 1 is the reaction chamber while chamber 2 serves as the CO-releasing chamber. The CO-releasing system^21^ was kept constant throughout the screening of reaction conditions, and chamber 2 thus contained 0.5 equiv of Mo(CO)_6_ in 2.5 mL of 1,4-dioxane with 2.5 equiv of DBU as the base that promotes the release of CO.^22^

**Table 1:**
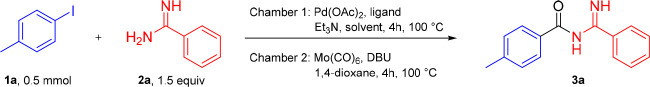
Optimization of Reaction Conditions for the Synthesis of Acyl Amidines from Aryl Iodides and Amidines^a^

entry	solvent	catalyst precursor	ligand	NMR yield (%)
1	DMA	5% Pd(OAc)_2_		61
2	DMF	5% Pd(OAc)_2_		74
3	DMSO	5% Pd(OAc)_2_		12
4	DMF	5% Pd(OAc)_2_	PPh_3_	92
5	DMF	5% Pd(PPh_3_)_4_		91
6	DMF	2.5% Pd(OAc)_2_	PPh_3_	12
7	DMF	10% Pd(OAc)_2_	PPh_3_	70
8	DMF	5% Pd(OAc)_2_	PPh_3_	76^b^
**9**	**DMF**	**5% Pd(OAc)**_**2**_	**PPh**_**3**_	**87**^c^

a NMR yield calculated by addition of benzyl alcohol as internal standard. Reaction conditions: In chamber 1 **1a** (0.5 mmol), **2a** (1.5 equiv), Pd(OAc)_2_, ligand (2:1 ligand:Pd ratio), and Et_3_N (2.5 equiv) were mixed in the specified solvent (2.5 mL), and in chamber 2 Mo(CO)_6_ (0.5 equiv) and DBU (2.5 equiv) were mixed in 1,4-dioxane (2.5 mL). Both chambers were capped and heated at 100 °C for 4 h.

b A 0.5 mmol amount of **2a** and 1.5 equiv of **1a**.

c Reaction run for 2 h at 80 °C.

Testing a number of suitable solvents with Pd(OAc)_2_ as the sole component of the catalytic system revealed that DMF was most productive, giving 74% NMR yield (Table 1, entry 2), compared to 61% and 12% for DMA and DMSO, respectively (entries 1 and 3). Adding PPh_3_ as a ligand (2:1 ratio to palladium) increased the yield to 92% (entry 4) with the use of Pd(PPh_3_)_4_ equally successful, giving 91% yield (entry 5). Increasing or decreasing the catalyst loading (10% or 2.5%) resulted in lower yields (70% and 12%, respectively, entries 7 and 6), and using **1a** in excess provided no added advantage (entry 8, 76%). Decreasing the time and temperature to 2 h and 80 °C gave a similar yield (entry 9, 87%, compare with entry 4). Thus, the reaction conditions were established using 5% Pd(OAc)_2_ and 10% PPh_3_ as the catalytic system in DMF, with the amidine nucleophile in excess toward the yield-determining aryl iodide.

Next, an investigation of the scope of the reaction with regard to the (hetero)aryl iodide partner was performed, see Table 2. Excellent yields were achieved for 4-methyl-, 3-methyl-, and 4-bromo-substituted iodobenzenes, furnishing 97%, 91%, and 90% of **3a**, **3c**, and **3d**, respectively. The thiophene derivative **3e** could be isolated in 63% yield from the corresponding iodide. Electron-poor 4-acetyl, 4-trifluoromethyl, and 3-nitro iodobenzenes gave varying yields of 83% (**3b**), 9% (**3g**), and 47% (**3i**) yields, respectively, whereas electron-rich 4-iodoanisole (**1f**) gave **3f** in 57% yield. Somewhat surprisingly, 2-methyl-substituted **3h** was only isolated in 20% yield, while 1-iodonaphtalene (**1k**) gave 39% isolated yield of **3k**. Unfortunately, pyridine derivative **3j** was only formed in trace amounts. At this point, in an attempt to improve the outcome for the less productive (hetero)aryl iodides, other ligands (DPEphos, Xantphos, dppp, and dppf) were tested. The change of ligand for aryl iodides **1f**–**1k** proved beneficial and resulted in improved yields for all but **3j**, albeit with different ligands. The yield for **3f** was improved from 57% to 87% by use of Xantphos, whereas the other ligands offered only slight improvements compared to PPh_3_. Xantphos also turned out to be beneficial in the synthesis of **3g** and **3i**, where the yields were drastically improved from 9% and 47% to 86% and 86%, respectively. For the sterically encumbered **3h**, DPEphos was the best ligand, and the yield was raised to 70% compared to the 20% obtained with PPh_3_. However, this strategy was not successful in the case of 1-naphthyl derivative **3k**, where the gain in yield was only modest with DPEphos. 2-Iodopyridine (**1j**) was not productive using DPEphos or Xantphos as ligand.

**Table 2:**
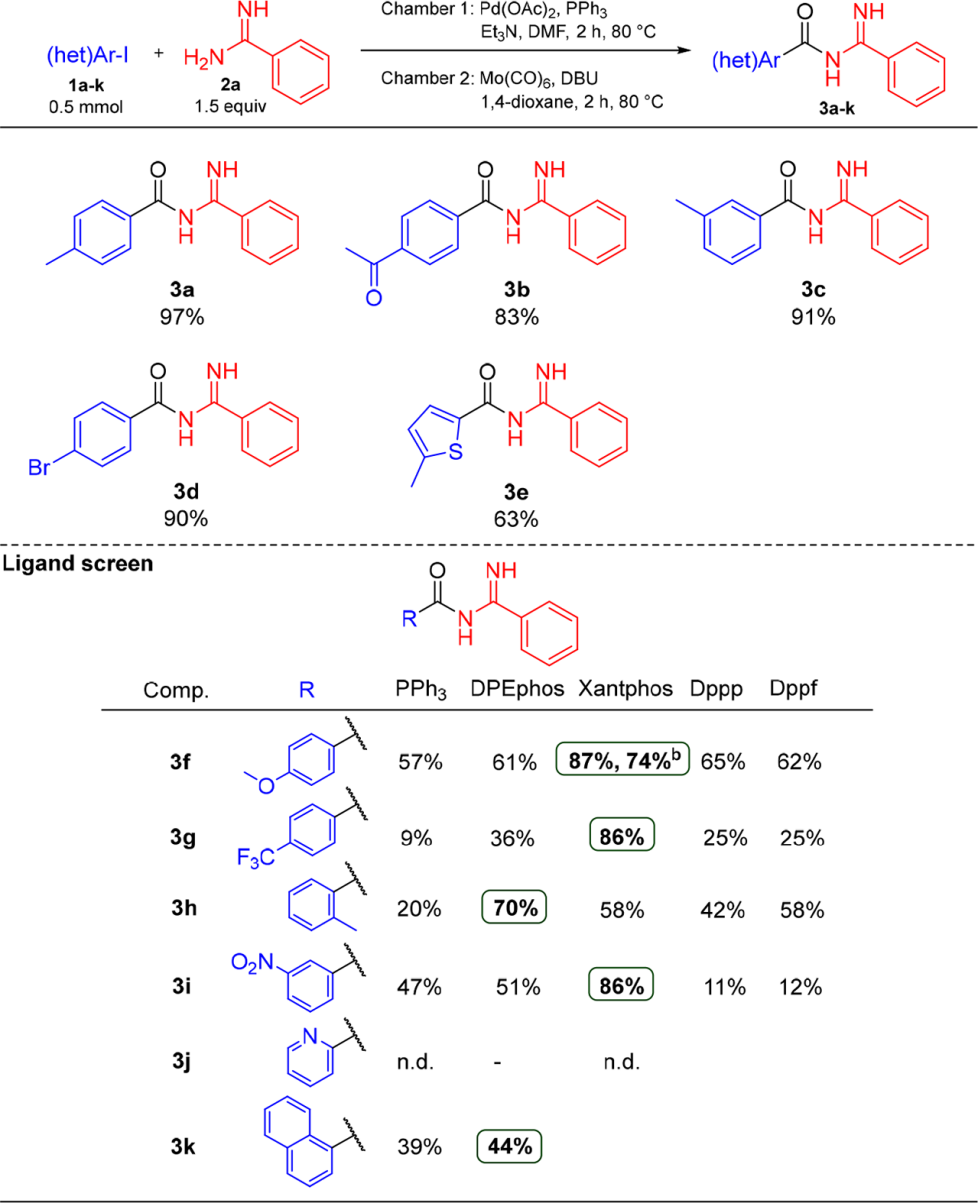
Investigation of the Aryl Iodide Scope for the Synthesis of Acyl Amidines^a^

a Isolated yield (>95% purity as determined by ^1^H NMR). Reaction conditions: In chamber 1 **1a**–**k** (0.5 mmol), **2a** (1.5 equiv), Pd(OAc)_2_ (5%), PPh_3_ (10%), and Et_3_N (2.5 equiv) were mixed in DMF (2.5 mL), and in chamber 2 Mo(CO)_6_ (0.5 equiv) and DBU (2.5 equiv) were mixed in 1,4-dioxane (2.5 mL). Both chambers capped and heated at 80 °C for 2 h. Ligand screen: DPEphos (5%), Xantphos (5%), dppp (5%), dppf (5%).

b One millimole scale.

The scope of the reaction with regard to the amidine nucleophile was also investigated, see Table 3. DPEphos was used as the ligand for the investigation as we reasoned that the combination of its bidentate nature and increased flexibility would provide the greatest generality. Pleasingly, the ligand was in general productive, affording yields of 34–71% for products **3l**–**v**, with aryl, heteroaryl, and alkyl amidines as nucleophiles. Specifically, electron-rich (hetero)aryl amidines worked well, giving a 71% isolated yield of **3l**, **3m**, and **3t**, respectively. The 3-pyridine derivative **3r** was isolated in 34% yield, whereas thiophene derivative **3s** was isolated in 43% yield. Alkyl amidines furnished yields of the corresponding products **3n**–**3q** in the range of 44–73%. Electron-poor aryl amidines afforded slightly lower yields, returning the chloro-substituted derivative **3u** in 45% yield and the trifluoromethyl-substituted product **3v** in 41% yield. Analogously to the less productive substrates in Table 2, **3u** was synthesized using Xantphos, dppp, dppf, and PPh_3_, thus affording **3u** in 57%, 20%, 76%, and 78% yield, respectively. Of note was the poor performance by Xantphos, whereas dppf and PPh_3_ returned the best yields and were also tested in the synthesis of **3v**. Both ligands improved the yield in a similar extent as for **3v**.

**Table 3:**
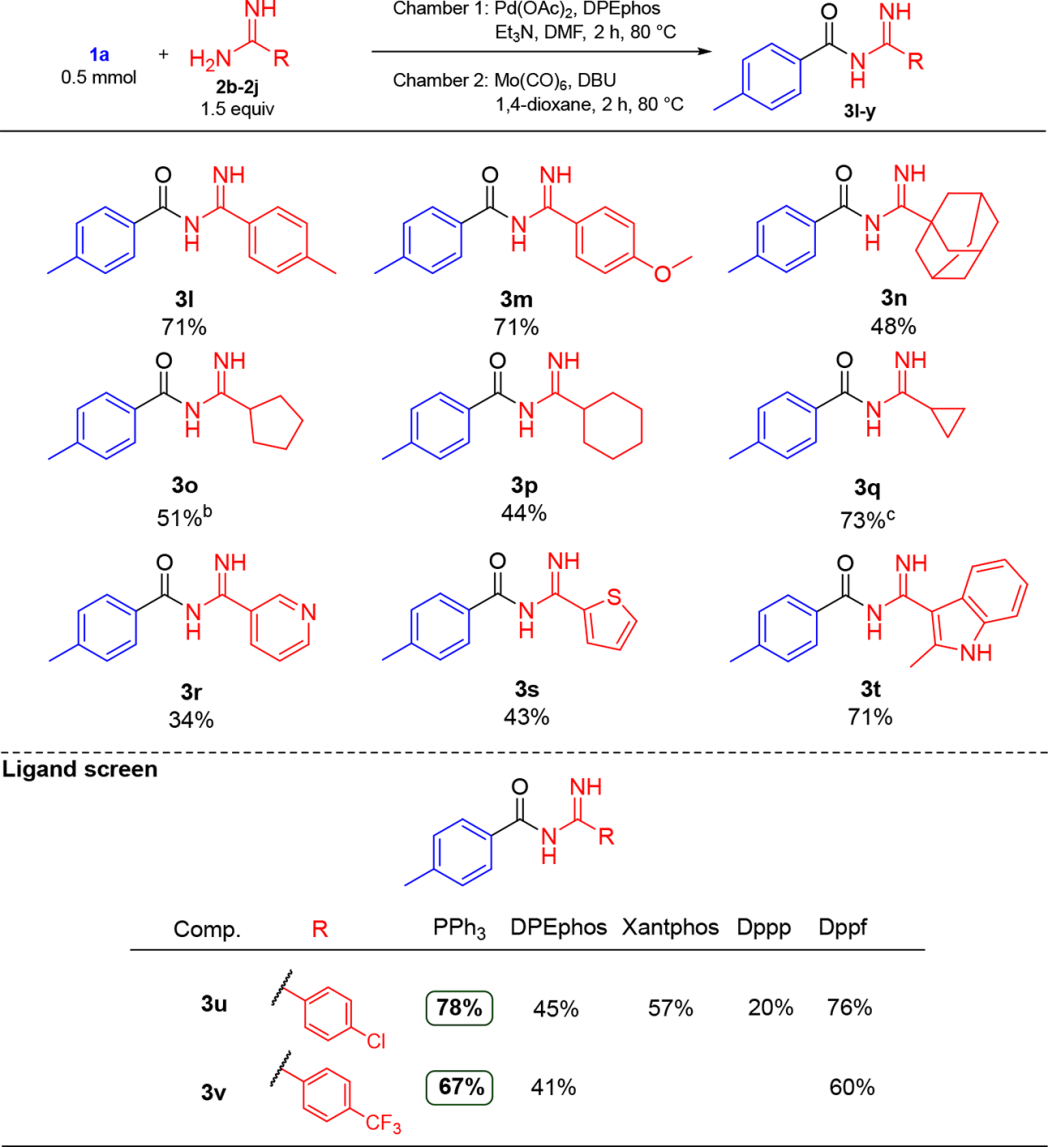
Investigation of the Amidine Scope of the Synthesis of Acyl Amidines^a^

a Isolated yield (>95% purity as determined by ^1^H NMR unless otherwise stated). Reaction conditions: In chamber 1 **1a** (0.5 mmol), **2b**–**2j** (1.5 equiv), Pd(OAc)_2_ (5%), DPEphos (5%), and Et_3_N (2.5 equiv) were mixed in DMF (2.5 mL), and in chamber 2 Mo(CO)_6_ (0.5 equiv) and DBU (2.5 equiv) were mixed in 1,4-dioxane (2.5 mL). Both chambers were capped and heated at 80 °C for 2 h. Ligand screen: PPh_3_ (10%), Xantphos (5%), dppp (5%), dppf (5%).

b **3o** 85% pure.

c **3q** > 90% purity as determined by ^1^H NMR.

Given the good performance of aryl iodides, we also opted to investigate aryl bromides as aryl–palladium precursors in this reaction, see Table 4. Initial screening revealed that the reaction time and temperature needed to be increased, and the reactions were run for 4 h at 100 °C. 4-Bromotoluene was productive with all ligands tested with good isolated yields using PPh_3_, Xantphos, and dppf, at 89%, 84%, and 72%, respectively. These three ligands were then used for investigation of electron-poor aryl bromide 4-bromoacetophenone and electron-rich aryl bromide 4-bromoanisole. Xantphos gave the best outcome for 4-bromoacetophenone with 78% yield compared with 19% yield with PPh_3_ and 58% yield with dppf. Xantphos was also the ligand of choice for 4-bromoanisole with 82% yield, while the performance of the other ligands was reversed in this case: PPh_3_ gave 79% yield and dppf 29% yield. For the substrate 2-bromotoluene, DPEphos was included in the investigation due to the favorable results for the corresponding iodine derivative. Notably, Xantphos was unproductive, and dppf was found to be the most productive ligand with 32% isolated yield.

**Table 4:**
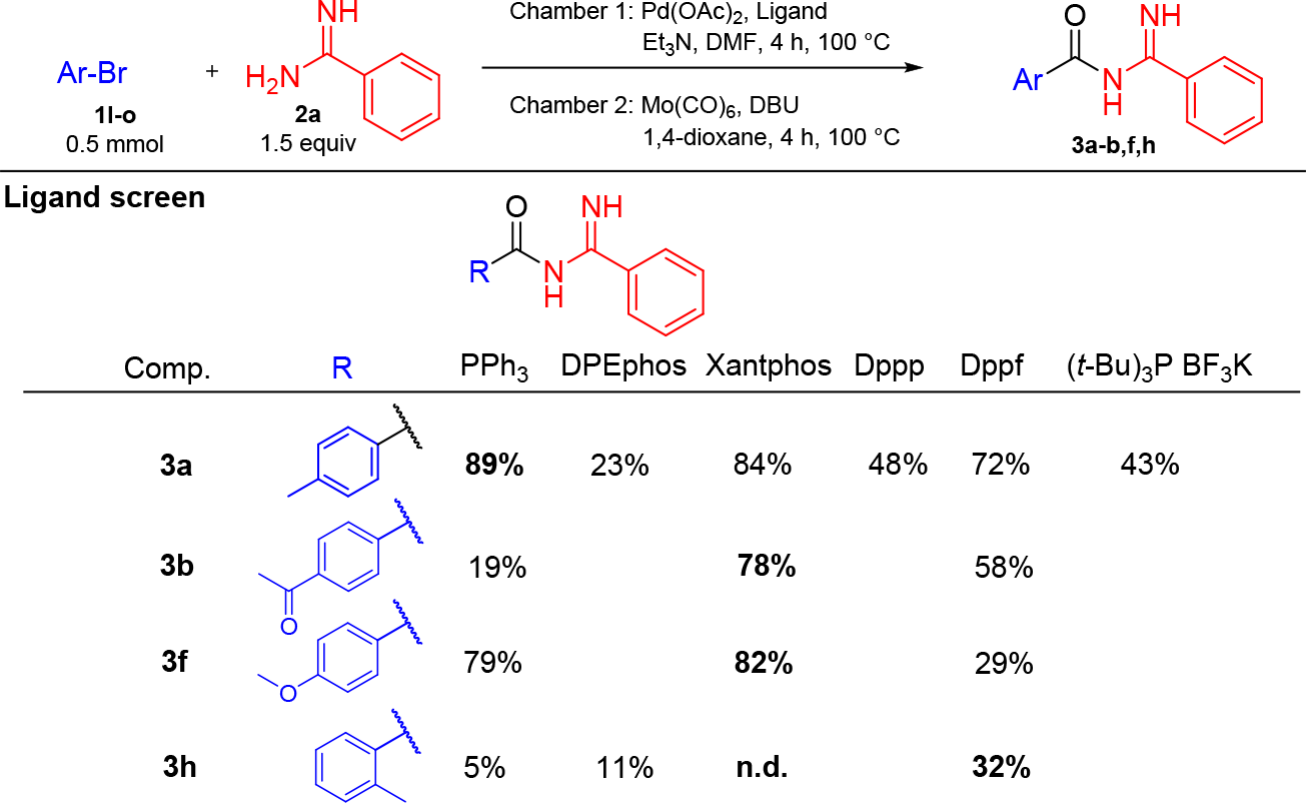
Investigation of the Aryl Bromide Scope for the Synthesis of Acyl Amidines^a^

a Isolated yield (>95% purity as determined by ^1^H). Reaction conditions: In chamber 1 **1l**–**o** (0.5 mmol), **2a** (1.5 equiv), Pd(OAc)_2_ (5%), ligand (X%), and Et_3_N (2.5 equiv) were mixed in DMF (2.5 mL), and in chamber 2 Mo(CO)_6_ (0.5 equiv) and DBU (2.5 equiv) were mixed in 1,4-dioxane (2.5 mL). Both chambers are capped and heated at 100 °C for 4 h. Ligand screen: PPh_3_ (10%), DPEphos (5%), Xantphos (5%), dppp (5%), dppf (5%), (*t*-Bu)_3_P BF_3_K (10%).

**Scheme 2:**
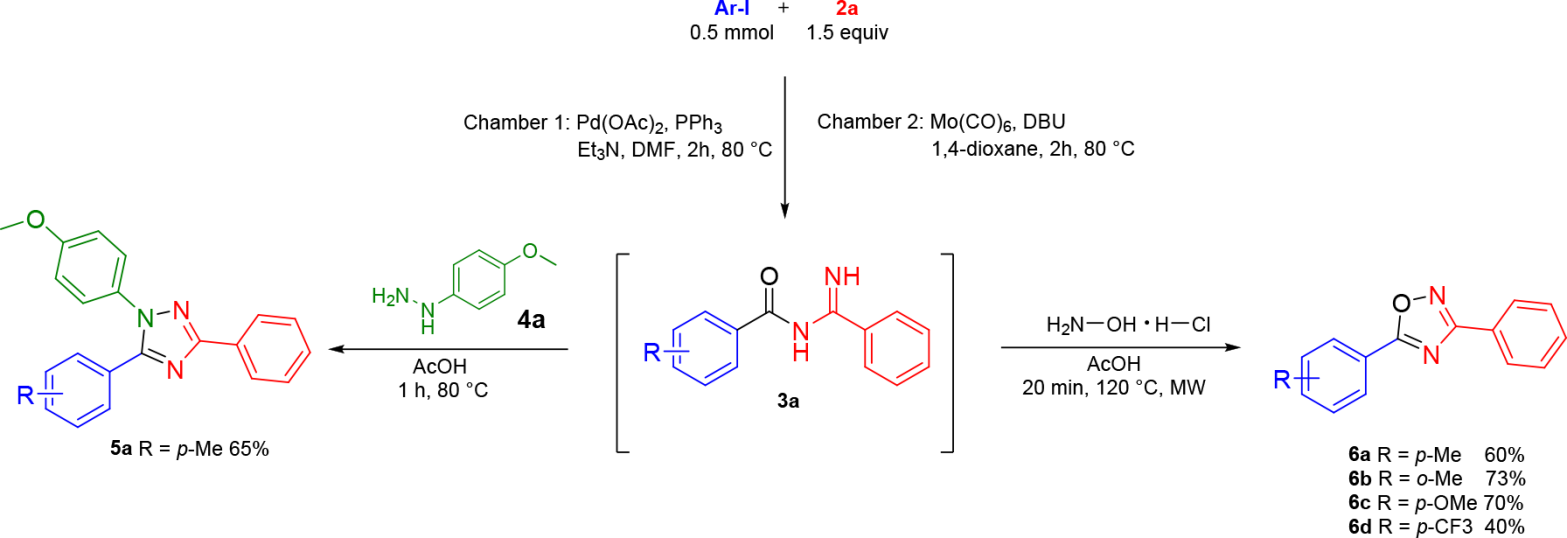
One-Pot, Two-Step Synthesis of 1,2,4-Triazole **5a** and 1,2,4-Oxadiazole **6a**–**6d** Isolated yield (>95% purity as determined by ^1^H NMR).

Having established viable conditions for the generation of the acyl amidines from aryl iodides/bromides and amidines, the investigation moved on to the direct use of the formed acyl amidine as an intermediate in the synthesis of 1,2,4-triazoles and 1,2,4-oxadiazoles. Pleasingly, an adaption of the protocol by Staben and Blaquiere^1^ with the conditions developed herein to generate the acyl amidine intermediate gave triazole **5** in 65% yield over two steps (Scheme 2).

1,2,4-Oxadiazoles are accessible from acyl amidines by reaction with hydroxylamine hydrochloride or sodium hypochlorite.^5,6^ After some experimentation, we discovered that the former reagent was suitable for the two-step one-pot preparation of 1,2,4-oxadiazoles. Specifically, we utilized this strategy to prepare unsymmetrical diaryl-1,2,4-oxadiazoles **6a**–**6d** in 40–73% yield from the respective aryl iodide and **2a** over two steps (Scheme 2).

**Scheme 3:**
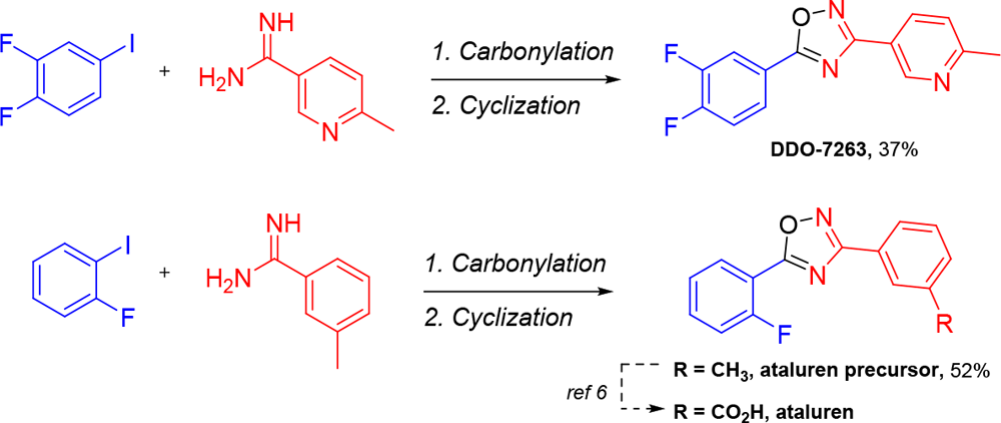
One-Pot, Two-Step Synthesis of Nrf2 Activator DDO-7263 and Ataluren Precursor (R = CH_3_) Isolated yield (>95% purity as determined by ^1^H NMR). Reaction conditions as described in Scheme 2.

To demonstrate the utility of this protocol for the synthesis of biologically active compounds, we opted to exemplify this with the synthesis of a Nrf2 activator called DDO-7263 and a precursor to ataluren, a drug used for treatment of Duchenne muscular dystrophy (Scheme 3). DDO-7263 is an Nrf2 activator, recently suggested to act on Rpn6 to regulate the Nrf2 signaling pathway.^23−25^ With our protocol, DDO-7263 could be synthesized in a one-pot, two-step fashion from commercially available starting materials. The yield of 37% is also higher than the overall yield of the first published literature procedure.^23^ In addition, an ataluren precursor was prepared from 2-fluoroiodobenzene and 3-methyl-benzamidine in 52% yield, which upon subsequent benzylic oxidation can give the Duchenne muscular dystrophy drug ataluren.^6^

## Radiochemistry

PET is a noninvasive imaging technique, widely used in cardiology, neurology, and oncology.^26−28^ PET has also found applications in drug development owing to the possibility to study the pharmacokinectics and the pharmacodynamics of labeled drug candidates in vivo.^29−31^ A radioisotope commonly incorporated in PET tracers is carbon-11 with a half-life of 20.4 min. With the possibility to produce carbon-11 in the form of [^11^C]CO, we sought to investigate the possibility of synthesizing ^11^C-labeled acyl amidines both for isolation and as a precursor for heterocycle synthesis.

To find conditions suitable for incorporation of carbon-11, a set of reactions was performed based on results from optimization of the Mo(CO)_6_ reaction using 4-iodoanisole (**1f**) and benzamidine (**2a**) as model compounds (Table 5). Starting with Pd(OAc)_2_ and Xantphos in DMF, the reaction was run at 120 °C for 10 min (entry 1). This resulted in 98% of the gaseous [^11^C]CO being trapped as nonvolatile ^11^C-labeled products ([^11^C]CO conversion, entry 1). The product selectivity, based on the crude HPLC chromatogram, was 49%, thereby giving a radiochemical yield (RCY, see SI for definitions and calculations) of 48% for ^**11**^**C-3f**. To improve the product selectivity, the ligand and solvent were changed to PPh_3_ in 1,4-dioxane, which gave a 74% isolated yield of **3f** using the conditions stated in Table 1. The product selectivity was improved, but a slight loss in [^11^C]CO conversion resulted in a similar RCY of 49% (entry 2). A further improvement in product selectivity was obtained with Pd(PPh_3_)_4_ (67%). However, Pd(PPh_3_)_4_ imposed solubility issues, and to simplify the subsequent HPLC purification step, the amount of Pd(PPh_3_)_4_ was reduced to 0.1 equiv. Although there was a loss in [^11^C]CO conversion, from 94% to 80%, the RCY was increased to 54% (entry 3). Running the reaction in DMF was very beneficial for the product selectivity (87%), but as the [^11^C]CO conversion dropped to 54%, the RCY was not improved compared to entry 3.

**Table 5:**
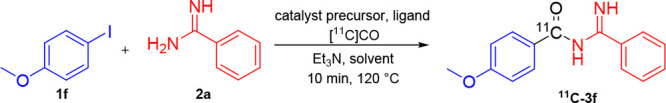
Screening Conditions for ^11^C Incorporation^a^

entry	catalyst precursor	ligand	solvent	[^11^ C]CO-conversion^b^ (%)	product selectivity (%)	RCY^c^ (%)	no. of exp
1	Pd(OAc)_2_	Xantphos	DMF	98 ± 0	49 ± 9	48 ± 9	3
2	Pd(OAc)_2_	PPh_3_	dioxane	91 ± 4	56 ± 7	49 ± 6	3
3	Pd(PPh_3_)_4_		dioxane	80 ± 5	67 ± 4	54 ± 6	3
4	Pd(PPh_3_)_4_		DMF	54 ± 4	87 ± 5	47 ± 6	3

a Reaction conditions: **1f** (9.0 μmol), **2a** (2.0 equiv), Pd(OAc)_2_ (0.5 equiv), Xantphos (1.0 equiv), PPh_3_ (1.0 equiv), Pd(PPh_3_)_4_ (0.1 equiv), Et_3_N (4 equiv). All dry regents were dissolved in 400 μL of solvent before addition of Et_3_N. See Supporting Information for definitions and calculations.

b Amount of [^11^C]CO converted to nonvolatile ^11^C-labeled products. Decay corrected.

c Radiochemical yield, RCY, estimated from the [^11^C]CO conversion and HPLC analysis of the crude reaction mixture.

Although the differences in the estimated RCY were small, the conditions from entry 3 were chosen for isolation of three ^11^C-labeled acyl amidine derivatives (Table 6). Electron-rich ^**11**^**C-3f** and electron-poor ^**11**^**C-3b** were gratifyingly isolated in 24% and 36% RCY, respectively. When shortening the reaction time to 5 min, the RCY dropped to 7% and 83 MBq ^**11**^**C-3f** was isolated after 37 min starting from 3.2 GBq [^11^C]CO. In comparison, with a 10 min reaction time, 190 MBq ^**11**^**C-3f** was isolated after 41 min starting from 3.5 GBq [^11^C]CO. Sterically hindered ^**11**^**C-3h**, however, was not formed under the conditions employed. No formation was seen even when changing the solvent to DMF, raising the reaction temperature to 150 °C, or changing the palladium source to Pd(OAc)_2_ and DPEphos as ligand.

**Table 6:**
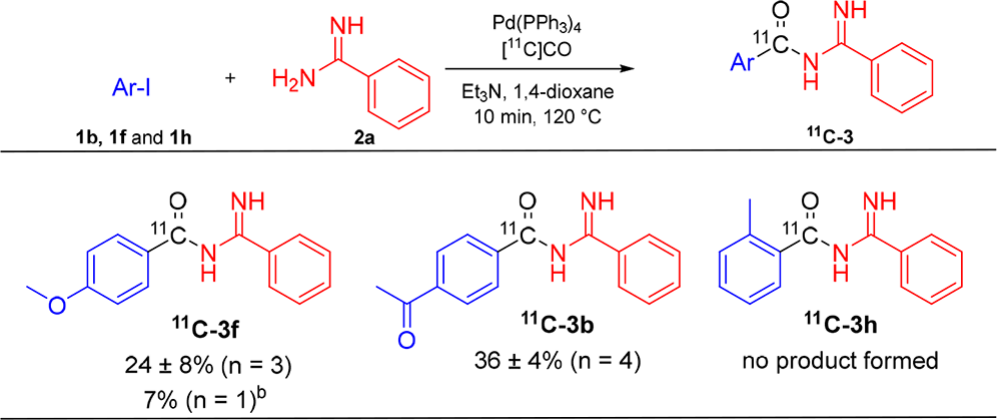
Synthesis of ^11^C-Labeled Acyl Amidines^a^

a Reaction conditions as in entry 2, Table 5. Radiochemical purity was >95% in all experiments, determined using two different HPLC columns. The reported radiochemical yields were calculated from the amount of [^11^C]CO collected in the reaction vial and the radioactivity of the isolated product (decay corrected). See Supporting Information for definitions and calculations.

b Five minute reaction time.

A principle of PET is the microdosing concept, i.e., only subpharmacological doses of the PET tracer should be injected.^32^ The concept of molar activity is therefore an important parameter for estimation of the amount of ^11^C-labeled tracer versus isotopically unmodified tracer in the isolated ^11^C-labeled product fraction. The molar activity was calculated for ^**11**^**C-3b** following two large irradiations. Starting from 14.3 and 15.4 GBq of [^11^C]CO and isolating 1.7 and 2.1 GBq, the molar activities of ^**11**^**C-3b** were at the end the purification, 488 and 650 GBq/μmol, respectively. The high molar activities are in line with previously reported results using [^11^C]CO.^33,34^

**Scheme 4:**
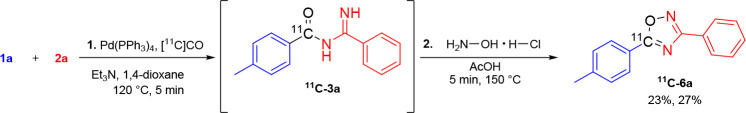
One-Pot, Two-Step Synthesis of 1,2,4-Oxadiazole **^11^C-6a** Reaction conditions for step 1 as described in Table 4, entry 2. Note that a 5 min reaction time was used in step 1. Step 2: Hydroxylamine hydrochloride (7 equiv) and 50% acetic acid (aq) were added to the reaction mixture. The reaction was heated at 150 °C for another 5 min.

Building on the successful one-pot carbonylation/cyclization sequence developed using Mo(CO)_6_, ^**11**^**C-6a** was synthesized from **1a** and **2a** (Scheme 4). The cyclization was tested with hydroxylamine hydrochloride and sodium hypochlorite, with only the former giving full consumption of the intermediate ^**11**^**C-3a** (HPLC analysis).^5,6^ Pleasingly, isotopically labeled 1,2,4-oxadiazole ^**11**^**C-6a** could be isolated in a decay-corrected RCY of 25% and in 99% radiochemical purity. The RCY was based on the starting amount of [^11^C]CO and the decay-corrected, isolated amount of ^**11**^**C-6a**.

Heterocyclic derivatives such as ^11^C-indole,^35^ substituted ^11^C-quinoxaline-2,3-diones,^36^ a substituted ^11^C-quinazoline-2,4(1*H*,3*H*)-dione,^37^ a substituted ^11^C-1,2,4-thiadiazolidine-3,5-dione,^37^ [*carbonyl*-^11^C]7-methyl-8-phenyl-3-trimethylsilyl-pyrazolo[5.1-c][1,2,4]triazine-(1*H*)4-one,^38^ and 2-[^11^C]thymidine^39^ have previously been synthesized from ^11^C precursors such as [^11^C]nitromethane, diethyl [^11^C]oxalate, [^11^C]carbon dioxide, [^11^C]lithium trimethylsilyl ynolate, and [^11^C]phosgene. This is, however, to the best of our knowledge, the first time that [^11^C]CO has been used in the synthesis of labeled acyl amidines (with ^11^C) and the first example of the synthesis of a ^11^C-labeled oxadiazole or a ring-atom-labeled heterocycle using [^11^C]CO. The method presented herein therefore complements other ^11^C-labeled precursors available for synthesis of ^11^C-labeled heterocyclic derivatives, thus opening up a significant new area of ^11^C chemical space.

## Conclusion

We have developed a protocol for the palladium-catalyzed carbonylative synthesis of acyl amidines from (hetero)aryl iodides or aryl bromides and amidines using a bridged two-vial system to generate CO gas ex situ from Mo(CO)_6_. Excellent yields can be achieved when using an appropriate ligand, with PPh_3_ generally working well for electron-rich and neutral (hetero)aryl iodides. DPEphos was shown to be a better choice for sterically hindered aryl iodides, whereas Xantphos was very productive for electron-poor aryl iodides. For less nucleophilic amidines, PPh_3_ and dppf were the ligands of choice. These results highlight the influence of subtle differences in substrate/ligand properties on the reaction outcome and serve as a reminder that a “one-ligand-for-all-substrates approach” is not always possible. In total, the scope and limitations of the reaction were demonstrated in over 25 diverse examples including more challenging aryl bromides. A new strategy for the one-pot, two-step synthesis of 1,2,4-oxadiazoles was also developed, allowing synthesis of unsymmetrically 3,5-substituted 1,2,4-oxadiazoles from (hetero)aryl iodides, amidines, CO, and hydroxylamine hydrochloride. These methods were also translated into a radiochemical setting and were successfully employed in a number of ^11^C-labeling examples with good radiochemical yields. Finally, the synthesis of a ^11^C-labeled 1,2,4-oxadiazole represents, to the best of our knowledge, the first incorporation of carbon-11 into a heterocyclic ring using [^11^C]CO, opening up a significant scope for new ^11^C chemistry development.

## Experimental Section

### General Chemistry Information

All substrates, reagents, and solvents were commercially available and used without further purification. Heating was carried out using a 17.4 mm DrySyn reaction vial insert compatible with the two-chamber system used for carbonylation. Microwave heating was performed using a Biotage Initiator 2.5 equipped with an IR sensor that is used to determine the temperature. Analytical reversed phase HPLC-MS was performed on a Dionex Ultimate 3000 system using 0.05% HCOOH in water and 0.05% HCOOH in acetonitrile as mobile phase with MS detection, equipped with a C18 (Phenomenex Kinetex SB-C18 (4.8 × 50 mm)) column using a UV diode array detector. Purifications were performed on an automated Biotage Isolera Flash Chromatography System using 25 or 10 g prepacked Biotage SNAP KP-SIL columns. Carbon-11 was prepared by the ^14^N(p,α)^11^C nuclear reaction using 17 MeV protons produced by a Scanditronix MC-17 Cyclotron at PET Centre, Uppsala University Hospital, and obtained as [^11^C]carbon dioxide. The target gas used was nitrogen (AGA Nitrogen 6.0) containing 0.05% oxygen (AGA Oxygen 4.8). Preparative purification of [^11^C] compounds was performed on a VWR La Prep Sigma system with a LP1200 pump, a 40D UV detector, a Bioscan flowcount radiodetector equipped with a Phenomenex Kinetex C18 (5 μm, 150 × 10.0 mm) column, and 0.1% trifluoroacetic acid (aq.) and acetonitrile as eluents. The identities, concentration, and radiochemical purities of the purified ^11^C-labeled compounds were determined with either (A) a VWR Hitachi Elite LaChrom system (L-2130 pump, L-2200 autosampler, L-2300 column oven, L-2450 diode array detector in series with a Bioscan β^+^-flowcount radiodetector) equipped with a Merck Chromolith Performance RP-18e column (4.6 × 100 mm) and ammonium formate buffer (pH 3.5) and acetonitrile as eluents or (B) an Elite LaChrom VWR International (LaPrep P206 pump, an Elite LaChrom L-2400 UV detector in series with a Bioscan β+-flowcount detector) equipped with a Reprosil-Pur Basic C18 (5 μm 4.6 × 100 mm) with 8.1 mM ammonium carbonate (aq.) and acetonitrile mobile phase and using isotopically unmodified compounds as references. Accurate mass values were determined on a mass spectrometer equipped with an electrospray ion source and TOF detector. NMR spectra were recorded on a Bruker Avance III HD at 25 °C and 400 MHz for ^1^H, 101 MHz for ^13^C, and ^19^F at 376.5 MHz using a SmartProbe BB/1H probe or on a Varian Mercury plus at 25 °C and 400 MHz for ^1^H, 101 MHz for ^13^C, and ^19^F at 376.5 MHz. Chemical shifts (δ) are reported in ppm, indirectly referenced to tetrametylsilane (TMS) via the residual solvent signal (^1^H: CHCl_3_ δ 7.26, CD_2_HOD δ 3.31, (CHD_2_)(CD_3_)SO δ 2.50, (CHD_2_)(CD_3_)CO δ 2.05. ^13^C: CDCl_3_ δ 77.2, CD_3_OD δ 49.0, (CHD_2_)(CD_3_)SO δ 39.5, (CD_3_)_2_CO δ 29.8, 206.3.)

### General Procedure for Synthesis of Acyl Amidines

The reaction was performed in a two-chamber system. Aryl halide (0.5 mmol) and amidine (1.5 equiv) were added to chamber 1 and dissolved in DMF (2 mL), followed by triethylamine (2.5 equiv) and Pd(OAc)_2_ (5 mol %). The reaction was briefly stirred before addition of monodentate ligand (10 mol %) or bidentate ligand (5 mol %) and remaining DMF (0.5 mL) followed by capping. To chamber 2 Mo(CO)_6_ (0.5 equiv) was added and dissolved in 1,4-dioxane (2.5 mL) followed by DBU (2.5 equiv) just before capping. The final concentration of the aryl halide in DMF was 0.2 M. Purification was done by direct injection on an automated Biotage Isolera Flash Chromatography System (silica gel, gradient elution using 0–100% EtOAc in isohexane, 22 CV).

#### *N*-(Imino(phenyl)methyl)-4-methylbenzamide, **3a** (CAS: 68167-55-5)

**3a** was synthesized according to the general procedure and isolated using automated flash chromatography (silica gel, gradient elution 0–100% EtOAc in isohexane over 22 CV) as an off-white amorphous solid (115 mg, 97% (PPh_3_) from **1a** and 106 mg, 89% (PPh_3_); 27 mg, 23% (DPEphos); 100 mg, 84% (Xantphos); 57 mg, 48% (Dppp); 86 mg, 72% (Dppf) and 51 mg, 43% ((*t*-Bu)_3_P BF_3_K) from **1l**). ^1^H NMR (400 MHz, CDCl_3_) δ 8.27 (d, *J* = 8.2 Hz, 2H), 8.06–8.01 (m, 2H), 7.61–7.55 (m, 1H), 7.54–7.48 (m, 2H), 7.28–7.23 (m, 2H), 2.42 (s, 3H). ^13^C{^1^H} NMR (101 MHz, CDCl_3_) δ 180.4 (carbonyl carbon, HMBC), 166.6, 142.8, 135.3, 135.1, 132.5 (2 × CH), 129.9 (2 × CH), 129.0 (4 × CH), 127.8 (2 × CH), 21.8. HRMS (ESI-TOF) *m*/*z*: [M + H]^+^ Calcd for C_15_H_15_N_2_O239.1184; Found 239.1193.

#### 4-Acetyl-*N*-(imino(phenyl)methyl)benzamide, **3b**

**3b** was synthesized according to the general procedure and isolated using automated flash chromatography (silica gel, gradient elution 0–100% EtOAc in isohexane over 22 CV) as an off-white amorphous solid (111 mg, 83% (PPh_3_) from **1b** and 25 mg, 19% (PPh_3_); 104 mg, 78% (Xantphos); 77 mg, 58% (Dppf) from **1n**). ^1^H NMR (400 MHz, (CD_3_)_2_CO) δ 10.85 (s, 1H), 8.66 (s, 1H), 8.48–8.45 (m, 2H), 8.30–8.26 (m, 2H), 8.10–8.06 (m, 2H), 7.68–7.63 (m, 1H), 7.60–7.54 (m, 2H), 2.64 (s, 3H). ^13^C{^1^H} NMR (101 MHz, (CD_3_)_2_CO) δ 197.9, 179.3, 168.3, 142.9, 140.4, 135.8, 133.2, 130.4, 129.5, 128.79, 128.75, 27.0. HRMS (ESI-TOF) *m*/*z*: [M + H]^+^ Calcd for C_16_H_15_N_2_O_2_ 267.1134; Found 267.1135.

#### *N*-(Imino(phenyl)methyl)-3-methylbenzamide, **3c**

**3c** was synthesized according to the general procedure and isolated using automated flash chromatography (silica gel, gradient elution 0–100% EtOAc in isohexane over 22 CV) as an off-white amorphous solid (108 mg, 91% (PPh_3_). ^1^H NMR (400 MHz, CDCl_3_) δ 10.74 (br s, 1H), 8.24–8.13 (m, 2H), 8.10–8.01 (m, 2H), 7.61–7.55 (m, 1H), 7.55–7.48 (m, 2H), 7.40–7.31 (m, 2H), 6.68 (br s, 1H), 2.44 (s, 3H). ^13^C{^1^H} NMR (101 MHz, CDCl_3_) δ 180.8, 166.7, 137.73, 137.68, 135.2, 132.9, 132.4, 130.2, 128.9, 128.0, 127.4, 126.9, 21.5. HRMS (ESI-TOF) *m*/*z*: [M + H]^+^ Calcd for C_15_H_15_N_2_O 239.1184; Found 239.1175.

#### 4-Bromo-*N*-(imino(phenyl)methyl)benzamide, **3d** (CAS: 68167-57-7)

**3d** was synthesized according to the general procedure and isolated using automated flash chromatography (silica gel, gradient elution 0–100% EtOAc in isohexane over 22 CV) as an off-white amorphous solid (137 mg, 90% (PPh_3_)). ^1^H NMR (400 MHz, CDCl_3_) δ 10.76 (br s, 1H), 8.27–8.21 (m, 2H), 8.06–8.00 (m, 2H), 7.63–7.56 (m, 3H), 7.56–7.49 (m, 2H), 6.68 (br s, 1H). ^13^C{^1^H} NMR (101 MHz, CDCl_3_) δ 179.6, 167.1, 136.8, 135.0, 132.7, 131.43, 131.42, 129.0, 127.5, 127.1. HRMS (ESI-TOF) *m*/*z*: [M + H]^+^ Calcd for C_14_H_12_BrN_2_O 303.0133; Found 303.0141.

#### *N*-(Imino(phenyl)methyl)-5-methylthiophene-2-carboxamide, **3e**

**3e** was synthesized according to the general procedure and isolated using automated flash chromatography (silica gel, gradient elution 0–100% EtOAc in isohexane over 22 CV) as an off-white amorphous solid (76 mg, 63% (PPh_3_)). ^1^H NMR (400 MHz, CDCl_3_) δ 10.50 (br s, 1H), 8.02–7.97 (m, 2H), 7.76 (d, *J* = 3.6 Hz, 1H), 7.60–7.54 (m, 1H), 7.53–7.47 (m, 2H), 6.81–6.77 (m, 1H), 6.57 (br s, 1H), 2.53 (d, *J* = 1.0 Hz, 3H). ^13^C{^1^H} NMR (101 MHz, CDCl_3_) δ 175.4, 166.2, 147.7, 141.4, 134.8, 132.5 (2 × CH), 128.9, 127.5, 126.7, 16.0. HRMS (ESI-TOF) *m*/*z*: [M + H]^+^ Calcd for C_13_H_13_N_2_OS 245.0749; Found 245.0760.

#### *N*-(Imino(phenyl)methyl)-4-methoxybenzamide, **3f** (CAS: 1445133-92-5)

**3f** was synthesized according to the general procedure and isolated using automated flash chromatography (silica gel, gradient elution 0–100% EtOAc in isohexane over 22 CV) as an off-white amorphous solid (72 mg, 57% (PPh_3_); 77 mg, 61% (DPEphos); 111 mg, 87% (Xantphos); 83 mg, 65% (dppp); 79 mg, 62% (dppf) from **1f** and 101 mg, 79% (PPh_3_); 105 mg, 82% (Xantphos); 37 mg, 29% (Dppf) from **1n**). **3f** was synthesized according to the general procedure, scaled up to 1 mmol scale, and isolated as an off-white amorphous solid (188 mg, 74% (Xantphos)). ^1^H NMR (400 MHz, CDCl_3_) δ 8.38–8.33 (m, 2H), 8.05–8.00 (m, 2H), 7.56 (ddt, *J* = 8.3, 6.5, 1.4 Hz, 1H), 7.51–7.46 (m, 2H), 6.97–6.92 (m, 2H), 3.86 (s, 3H). ^13^C{^1^H} NMR (101 MHz, CDCl_3_) δ 180.1, 166.3, 162.9, 135.4, 132.3, 131.9, 130.7, 128.9, 127.5, 113.4, 55.5. HRMS (ESI-TOF) *m*/*z*: [M + H]^+^ Calcd for C_15_H_15_N_2_O_2_ 255.1134; Found 255.1138.

#### *N*-(Imino(phenyl)methyl)-4-(trifluoromethyl)benzamide, **3g** (CAS: 2052280-77-8)

**3g** was synthesized according to the general procedure and isolated using automated flash chromatography (silica gel, gradient elution 0–100% EtOAc in isohexane over 22 CV) as an off-white amorphous solid (13 mg, 9% (PPh_3_); 53 mg, 36% (DPEphos); 124 mg, 86% (Xantphos); 36 mg, 25% (dppp); 36 mg, 25% (dppf)). ^1^H NMR (400 MHz, CDCl_3_) δ 10.83 (br s, 1H), 8.48 (d, *J* = 8.0 Hz, 2H), 8.07–8.02 (m, 2H), 7.71 (d, *J* = 8.2 Hz, 2H), 7.65–7.60 (m, 1H), 7.57–7.51 (m, 2H), 6.79 (br s, 1H). ^13^C{^1^H} NMR (101 MHz, CDCl_3_) δ 177.2 (carbonyl carbon, HMBC), 167.5, 140.9, 134.8, 133.3 (d, ^*2*^*J*_CF_ = 32.3 Hz), 132.9, 130.1, 129.1, 127.6, 125.2 (q, ^*3*^*J*_CF_ = 3.8 Hz), 122.8 (d, ^*1*^*J*_CF_ = 272.3 Hz).^19^F NMR (376 MHz, CDCl_3_) δ −62.8. HRMS (ESI-TOF) *m*/*z*: [M + H]^+^ Calcd for C_15_H_12_F_3_N_2_O 293.0902; Found 293.0898.

#### *N*-(Imino(phenyl)methyl)-2-methylbenzamide, **3h** (CAS: 872266-80-3)

**3h** was synthesized according to the general procedure and isolated using automated flash chromatography (silica gel, gradient elution 0–100% EtOAc in isohexane over 22 CV) as an off-white amorphous solid (24 mg, 20% (PPh_3_); 83 mg, 70% (DPEphos); 69 mg, 58% (Xantphos); 50 mg, 42% (dppp); 69 mg, 58% (dppf) from **1h** and 7 mg, 5% (PPh_3_); 14 mg, 11% (DPEphos); 38 mg, 32% (Dppf) from **1o**). ^1^H NMR (400 MHz, CDCl_3_) δ 8.24–8.12 (m, 1H), 8.00 (d, *J* = 7.9 Hz, 2H), 7.59–7.54 (m, 1H), 7.53–7.46 (m, 2H), 7.37–7.32 (m, 1H), 7.29–7.21 (m, 2H), 2.69 (s, 3H). ^13^C{^1^H} NMR (101 MHz, CDCl_3_) δ 166.2 (HMBC), 139.0, 137.9, 135.3, 132.4, 131.6, 130.8 (2 × CH), 129.0, 127.5, 125.6, 22.0. Carbonyl carbon missing. HRMS (ESI-TOF) *m*/*z*: [M + H]^+^ Calcd for C_15_H_15_N_2_O 239.1184; Found 239.1191.

#### *N*-(Imino(phenyl)methyl)-3-nitrobenzamide, **3i**

**3i** was synthesized according to the general procedure and isolated using automated flash chromatography (silica gel, gradient elution 0–100% EtOAc in isohexane over 22 CV) as an off-white amorphous solid (63 mg, 47% (PPh_3_); 69 mg, 51% (DPEphos); 116 mg, 86% (Xantphos); 15 mg, 11% (dppp); 16 mg, 12% (dppf)). ^1^H NMR (400 MHz, CDCl_3_) δ 10.85 (s, 1H), 9.23–9.19 (m, 1H), 8.71–8.65 (m, 1H), 8.37 (ddd, *J* = 8.2, 2.4, 1.2 Hz, 1H), 8.10–8.03 (m, 2H), 7.68–7.60 (m, 2H), 7.60–7.52 (m, 2H), 6.81 (s, 1H). ^13^C{^1^H} NMR (101 MHz, CDCl_3_) δ 177.9, 167.7, 148.3, 139.6, 135.4, 134.4, 132.9, 129.11, 129.05, 127.5, 126.3, 124.7. HRMS (ESI-TOF) *m*/*z*: [M + H]^+^ Calcd for C_14_H_12_N_3_O_3_ 270.0879; Found 270.0884.

#### *N*-(Imino(phenyl)methyl)-1-naphthamide, **3k** (CAS: 101716-52-3)

**3k** was synthesized according to the general procedure and isolated using automated flash chromatography (silica gel, gradient elution 0–100% EtOAc in isohexane over 22 CV) as an off-white amorphous liquid (54 mg, 39% (PPh_3_); 60 mg, 44% (DPEphos)). ^1^H NMR (400 MHz, (CD_3_)_2_CO δ 10.80 (br s, 1H), 9.20 (d, *J* = 8.5 Hz, 1H), 8.57 (d, *J* = 7.1 Hz, 1H), 8.27–8.23 (m, 2H), 8.05 (d, *J* = 8.1 Hz, 1H), 7.98–7.94 (m, 1H), 7.65–7.51 (m, 6H). ^13^C{^1^H} NMR (101 MHz, CDCl_3_) δ 183.2 (carbonyl carbon, HMBC), 166.5, 135.6, 135.2, 134.1, 132.4, 132.1, 131.5, 130.0, 129.0, 128.5, 127.5, 127.1, 126.7, 125.9, 124.9. HRMS (ESI-TOF) *m*/*z*: [M + H]^+^ Calcd for C_18_H_15_N_2_O 275.1184; Found 275.1178.

#### *N*-(Imino(*p*-tolyl)methyl)-4-methylbenzamide, **3l**

**3l** was synthesized according to the general procedure and isolated using automated flash chromatography (silica gel, gradient elution 0–100% EtOAc in isohexane over 22 CV) as an off-white amorphous solid (90 mg, 71% (DPEphos)). ^1^H NMR (400 MHz, CDCl_3_) δ 10.69 (br s, 1H), 8.27 (d, *J* = 8.2 Hz, 2H), 7.93 (d, *J* = 8.2 Hz, 2H), 7.27 (dd, *J* = 15.4, 8.0 Hz, 4H), 6.67 (br s, 1H), 2.44–2.40 (m, 6H). ^13^C{^1^H} NMR (101 MHz, CDCl_3_) δ 180.5, 166.5, 143.1, 142.6, 135.3, 132.4, 129.9, 129.6, 128.9, 127.5, 21.8, 21.7. HRMS (ESI-TOF) *m*/*z*: [M + H]^+^ Calcd for C_16_H_17_N_2_O 253.1341; Found 253.1344.

#### *N*-(Imino(4-methoxyphenyl)methyl)-4-methylbenzamide, **3m**

**3m** was synthesized according to the general procedure and isolated using automated flash chromatography (silica gel, gradient elution 0–100% EtOAc in isohexane over 22 CV) as an off-white amorphous solid (95 mg, 71% (DPEphos)). ^1^H NMR (400 MHz, CDCl_3_) δ 8.29–8.24 (m, 2H), 8.04–7.99 (m, 2H), 7.27–7.23 (m, 2H), 7.00–6.94 (m, 2H), 3.86 (s, 3H), 2.42 (s, 3H). ^13^C{^1^H} NMR (101 MHz, CDCl_3_ and (CD_3_)_2_CO) δ 180.3, 166.1, 163.1, 142.5, 135.4, 129.8, 129.4, 128.9, 127.3, 114.2, 55.6, 21.8. HRMS (ESI-TOF) *m*/*z*: [M + H]^+^ Calcd for C_16_H_17_N_2_O_2_ 269.1290; Found 269.1284.

#### *N*-(Adamantan-1-yl(imino)methyl)-4-methylbenzamide, **3n**

**3n** was synthesized according to the general procedure and isolated using automated flash chromatography (silica gel, gradient elution 0–100% EtOAc in isohexane over 22 CV) as an off-white amorphous solid (71 mg, 48% (DPEphos)). ^1^H NMR (400 MHz, CDCl_3_) δ 10.62 (br s, 1H), 8.18 (d, *J* = 8.1 Hz, 2H), 7.21 (d, *J* = 8.1 Hz, 2H), 6.26 (br s, 1H), 2.40 (s, 3H), 2.14–2.08 (m, 3H), 2.00–1.96 (m, 6H), 1.82–1.72 (m, 7H). ^13^C{^1^H} NMR (101 MHz, (CD_3_)_2_CO) δ 180.2, 180.0, 142.4, 137.0, 130.3, 129.3, 41.1, 40.4, 37.3, 29.3, 21.5. HRMS (ESI-TOF) *m*/*z*: [M + H]^+^ Calcd for C_19_H_24_N_2_O 297.1967; Found 297.1973.

#### *N*-(Cyclopentyl(imino)methyl)-4-methylbenzamide, **3o**

**3o** was synthesized according to the general procedure and isolated using automated flash chromatography (silica gel, gradient elution 0–100% EtOAc in isohexane over 22 CV) as an off-white amorphous solid (59 mg, 51%, 85% pure (DPEphos)). ^1^H NMR (400 MHz, CDCl_3_) δ 10.33 (br s, 1H), 8.17–8.12 (m, 2H), 7.23–7.18 (m, 2H), 6.12 (br s, 1H), 2.67 (quint, *J* = 8.0 Hz, 1H), 2.39 (s, 3H), 2.06–1.78 (m, 6H), 1.72–1.61 (m, 2H). ^13^C{^1^H} NMR (101 MHz, (CD_3_)_2_CO) δ 179.1, 165.3, 142.3, 129.8, 128.8, 127.8, 47.6, 31.6, 26.2. HRMS (ESI-TOF) *m*/*z*: [M + H]^+^ Calcd for C_14_H_19_N_2_O 231.1497; Found 231.1502.

#### *N*-(Cyclohexyl(imino)methyl)-4-methylbenzamide, **3p**

**3p** was synthesized according to the general procedure and isolated using automated flash chromatography (silica gel, gradient elution 0–100% EtOAc in isohexane over 22 CV) as an off-white amorphous solid (54 mg, 44% (DPEphos)). ^1^H NMR (400 MHz, CDCl_3_) δ 10.38 (br s, 1H), 8.15 (d, *J* = 8.1 Hz, 2H), 7.21 (d, *J* = 8.1 Hz, 2H), 6.16 (br s, 1H), 2.39 (s, 3H), 2.23 (tt, *J* = 11.8, 3.4 Hz, 1H), 2.03–1.96 (m, 2H), 1.89–1.81 (m, 2H), 1.77–1.70 (m, 1H), 1.58–1.46 (m, 2H), 1.40–1.19 (m, 3H). ^13^C{^1^H} NMR (101 MHz, CDCl_3_) δ 180.6, 176.5, 142.3, 135.3, 129.7, 128.8, 46.7, 30.7, 26.0, 25.9, 21.7. HRMS (ESI-TOF) *m*/*z*: [M + H]^+^ Calcd for C_15_H_21_N_2_O245.1654; Found 245.1661.

#### *N*-(Cyclopropyl(imino)methyl)-4-methylbenzamide, **3q**

**3q** was synthesized according to the general procedure and isolated using automated flash chromatography (silica gel, gradient elution 0–100% EtOAc in isohexane over 22 CV) as an off-white amorphous solid (74 mg, 73%, >90% pure (DPEphos)). ^1^H NMR (400 MHz, CDCl_3_) δ 10.40 (br s, 1H), 8.12–8.06 (m, 2H), 7.22–7.16 (m, 2H), 6.49 (br s, 1H), 2.39 (s, 3H), 1.43–1.36 (m, 1H), 1.34–1.28 (m, 2H), 0.99–0.92 (m, 2H). ^13^C{^1^H} NMR (101 MHz, CDCl_3_) δ 180.0, 174.7, 142.2, 135.4, 129.7, 128.8, 21.7, 17.1, 9.5. HRMS (ESI-TOF) *m*/*z*: [M + H]^+^ Calcd for C_12_H_15_N_2_O 203.1184; Found 203.1190.

#### *N*-(Imino(pyridin-3-yl)methyl)-4-methylbenzamide, **3r**

**3r** was synthesized according to the general procedure and isolated using automated flash chromatography (silica gel, gradient elution 0–100% EtOAc in isohexane over 22 CV) as an off-white amorphous solid (41 mg, 34% (DPEphos)). ^1^H NMR (400 MHz, CDCl_3_) δ 9.24 (s, 1H), 8.81–8.75 (m, 1H), 8.40–8.34 (m, 1H), 8.28–8.20 (m, 2H), 7.47–7.42 (m, 1H), 7.27–7.23 (m, 2H), 2.42 (s, 3H). ^13^C{^1^H} NMR (101 MHz, CDCl_3_) δ 180.6, 164.4, 152.9, 148.7, 143.1, 135.5, 134.8, 131.3, 129.9, 129.1, 123.7, 21.8. HRMS (ESI-TOF) *m*/*z*: [M + H]^+^ Calcd for C_14_H_14_N_3_O 240.1137 Found; 240.1132.

#### *N*-(Imino(thiophen-2-yl)methyl)-4-methylbenzamide, **3s** (CAS: 883041-84-7)

**3s** was synthesized according to the general procedure and isolated using automated flash chromatography (silica gel, gradient elution 0–100% EtOAc in isohexane over 22 CV) as an off-white amorphous solid (52 mg, 43% (DPEphos)). ^1^H NMR (400 MHz, CDCl_3_) δ 8.25–8.21 (m, 2H), 7.65 (dd, *J* = 3.8, 1.1 Hz, 1H), 7.57 (dd, *J* = 5.1, 1.0 Hz, 1H), 7.27–7.23 (m, 2H), 7.13 (dd, *J* = 5.0, 3.8 Hz, 1H), 2.41 (s, 3H). ^13^C{^1^H} NMR (101 MHz, CDCl_3_) δ 180.3, 160.9, 142.8, 140.3, 134.9, 132.2, 129.8, 129.0, 128.32, 128.25, 21.8. HRMS (ESI-TOF) *m*/*z*: [M + H]^+^ Calcd for C_13_H_13_N_2_OS 245.0749; Found 245.0759.

#### *N*-(Imino(2-methyl-1*H*-indol-3-yl)methyl)-4-methylbenzamide, **3t**

**3t** was synthesized according to the general procedure and isolated using automated flash chromatography (silica gel, gradient elution 0–100% EtOAc in isohexane over 22 CV) as an off-white amorphous solid (103 mg, 71% (DPEphos)). ^1^H NMR (400 MHz, (CD_3_)_2_SO) δ 11.77 (s, 1H), 8.11 (d, *J* = 8.2 Hz, 2H), 8.01–7.97 (m, 1H), 7.40–7.37 (m, 1H), 7.27 (d, *J* = 7.9 Hz, 2H), 7.15–7.12 (m, 2H), 2.79 (s, 3H), 2.37 (s, 3H). ^13^C{^1^H} NMR (101 MHz, (CD_3_)_2_SO) δ 177.9, 165.7, 141.5, 141.1, 136.2, 135.0, 128.9, 128.7, 126.4, 121.5, 120.5, 119.9, 111.2, 107.8, 21.1, 14.4. HRMS (ESI-TOF) *m*/*z*: [M + H]^+^ Calcd for C_18_H_18_N_3_O 292.1450; Found 292.1453.

#### *N*-((4-Chlorophenyl)(imino)methyl)-4-methylbenzamide, **3u**

**3u** was synthesized according to the general procedure and isolated using automated flash chromatography (silica gel, gradient elution 0–100% EtOAc in isohexane over 22 CV) as an off-white amorphous solid (61 mg, 45% (DPEphos); 78 mg, 57% (Xantphos); 27 mg, 20% (dppp); 103 mg, 76% (dppf); 106 mg, 78% (PPh_3_)). ^1^H NMR (400 MHz, CDCl_3_) δ 8.26–8.21 (m, 2H), 8.00–7.95 (m, 2H), 7.48–7.43 (m, 2H), 7.28–7.23 (m, 2H), 2.42 (s, 3H). ^13^C{^1^H} NMR (101 MHz, CDCl_3_) δ 180.6, 165.4, 142.9, 138.7, 135.0, 133.7, 129.9, 129.2, 129.01, 128.94, 21.8. HRMS (ESI-TOF) *m*/*z*: [M + H]^+^ Calcd for C_15_H_14_ClN_2_O 273.0795; Found 273.0793.

#### *N*-(Imino(4-(trifluoromethyl)phenyl)methyl)-4-methylbenzamide, **3v**

**3v** was synthesized according to the general procedure and isolated using automated flash chromatography (silica gel, gradient elution 0–100% EtOAc in isohexane over 22 CV) as an off-white amorphous solid (63 mg, 41% (DPEphos), 92 mg, 60% (dppf), 103 mg, 67% (PPh_3_)). ^1^H NMR (400 MHz, CDCl_3_) δ 8.25 (d, *J* = 8.0 Hz, 2H), 8.13 (d, *J* = 8.2 Hz, 2H), 7.75 (d, *J* = 8.2 Hz, 2H), 7.26 (d, *J* = 8.0 Hz, 2H), 2.42 (s, 3H). ^13^C{^1^H} NMR (101 MHz, CDCl_3_) δ 180.6, 165.0, 143.0, 138.7, 134.7, 133.6 (q, *J* = 32.6 Hz), 129.8, 128.9, 127.9, 125.8 (q, *J* = 3.7 Hz), 123.7 (q, *J* = 272.7 Hz), 21.7. ^19^F NMR (376 MHz, CDCl_3_) δ −63.0. HRMS (ESI-TOF) *m*/*z*: [M + H]^+^ Calcd for C_16_H_14_F_3_N_2_O 307.1058; Found 307.1051.

### Synthesis of Compound **5**

#### 1-(4-Methoxyphenyl)-3-phenyl-5-(*p*-tolyl)-1*H*-1,2,4-triazole, **5**

Step 1: The reaction was performed in a two-chamber system. 4-Iodotoluene (109 mg, 0.5 mmol) and benzamidine (90 mg, 0.75 mmol) were added to chamber 1 and dissolved in DMF (2 mL), followed by triethylamine (0.174 mL, 1.25 mmol) and Pd(OAc)_2_ (5.6 mg, 0.025 mmol). The reaction was swiftly stirred before addition of triphenylphosphine (13.1 mg, 0.05 mmol) and DMF (0.5 mL) followed by capping. To chamber 2 Mo(CO)_6_ (66 mg, 0.25 mmol) was added and dissolved in 1,4-dioxane (2.5 mL) followed by DBU (0.187 mL, 1.25 mmol) just before capping. The two-chamber system was heated at 80 °C for 2 h. After completion, the reaction was cooled and vented. Step 2: The reaction mixture was transferred to another reaction vial, and 4-methoxyphenylhydrazine (415 mg, 3 mmol) was added together with acetic acid (2 mL). The vial was sealed and heated at 80 °C for 1 h. After cooling to room temperature, the reaction mixture was diluted with 50 mL of ethyl acetate and washed with 5% NaOH (2 × 10 mL) and brine (10 mL). The organic layer was dried over MgSO_4_ and concentrated on a rotatory evaporator. The residue was purified by flash chromatography (silica gel, 5–50% EtOAc in isohexane) to give the desired product as colorless solid.

It was isolated by flash chromatography (silica gel, 5–50% EtOAc in isohexane) as an off-white amorphous solid (111 mg, 65%). ^1^H NMR (400 MHz, CDCl_3_) δ 8.32–7.99 (m, 2H), 7.49–7.39 (m, 5H), 7.38–7.30 (m, 2H), 7.16 (d, *J* = 7.9 Hz, 2H), 7.01–6.90 (m, 2H), 3.86 (s, 3H), 2.36 (s, 3H). ^13^C{^1^H} NMR (101 MHz, CDCl_3_) δ 161.6, 159.8, 154.8, 140.1, 131.5, 130.9, 129.3, 129.2, 128.8, 128.5, 127.0, 126.6, 125.2, 114.5, 55.6, 21.4. HRMS (ESI-TOF) *m*/*z*: [M + H]^+^ Calcd for C_22_H_20_N_3_O 342.1606; Found 342.1616.

### General Procedure for the Synthesis of 1,2,4-Oxadiazoles

Step 1: Performed according to the general procedure for synthesis of aryl amidines. Step 2: The reaction mixture was transferred to another reaction vial. Hydroxylamine hydrochloride (5 mmol) and acetic acid (1 mL) were added to the reaction mixture. The reaction vial was sealed and heated in a microwave at 120 °C for 20 min. After cooling to room temperature, the reaction mixture was diluted with 50 mL of ethyl acetate and washed with 5% NaOH (2 × 10 mL) and brine (10 mL). The organic layer was dried over MgSO_4_ and concentrated on a rotatory evaporator. The residue was purified by flash chromatography (silica gel, 0–30% EtOAc in isohexane) to give the desired product as colorless solid.

#### 3-Phenyl-5-(*p*-tolyl)-1,2,4-oxadiazole,^40^**6a** (CAS: 16112-24-6)

**6a** was synthesized according to the general procedure, with PPh_3_ as ligand, and isolated by flash chromatography (silica gel, 0–30% EtOAc in isohexane) as an off-white amorphous solid (71 mg, 60%). ^1^H NMR (400 MHz, CDCl_3_) δ 8.21–8.15 (m, 2H), 8.14–8.05 (m, 2H), 7.51 (dd, *J* = 5.1, 2.0 Hz, 3H), 7.36 (d, *J* = 8.0 Hz, 2H), 2.46 (s, 3H). ^13^C{^1^H} NMR (101 MHz, CDCl_3_) δ 175.9, 168.9, 143.5, 131.1, 129.8, 128.8, 128.2, 127.5, 127.1, 121.6, 21.8. HRMS (ESI-TOF) *m*/*z*: [M + H]^+^ Calcd for C_15_H_13_N_2_O 237.1028; Found 237.1027.

#### 3-Phenyl-5-(*o*-tolyl)-1,2,4-oxadiazole, **6b** (CAS: 54494-15-4)

**6b** was synthesized according to the general procedure, with DPEphos as ligand, and isolated by flash chromatography (silica gel, 0–30% EtOAc in isohexane) as an off-white amorphous solid (85 mg, 73%). ^1^H NMR (400 MHz, CDCl_3_) δ 8.21–8.15 (m, 3H), 7.55–7.50 (m, 3H), 7.50–7.46 (m, 1H), 7.40–7.35 (m, 2H), 2.79 (s, 3H). ^13^C{^1^H} NMR (101 MHz, CDCl_3_) δ 176.5, 168.7, 139.3, 132.3, 132.1, 131.3, 130.4, 129.0, 127.7, 127.3, 126.4, 123.6, 22.1. HRMS (ESI-TOF) *m*/*z*: [M + H]^+^ Calcd for C_15_H_13_N_2_O 237.1028; Found 237.1034

#### 5-(4-Methoxyphenyl)-3-phenyl-1,2,4-oxadiazole,^41^**6c** (CAS: 36364-17-7)

**6c** was synthesized according to the general procedure, with Xantphos as ligand, and isolated by flash chromatography (silica gel, 0–30% EtOAc in isohexane) as an off-white amorphous solid (88 mg, 70%). ^1^H NMR (400 MHz, CDCl_3_) δ 8.19–8.13 (m, 4H), 7.53–7.49 (m, 3H), 7.07–7.02 (m, 2H), 3.91 (s, 3H). ^13^C{^1^H} NMR (101 MHz, CDCl_3_) δ 175.7, 169.0, 163.3, 131.2, 130.2, 129.0, 127.7, 127.3, 117.0, 114.7, 55.7. HRMS (ESI-TOF) *m*/*z*: [M + H]^+^ Calcd for C_15_H_13_N_2_O_2_ 253.0977; Found 253.0981.

#### 3-Phenyl-5-(4-(trifluoromethyl)phenyl)-1,2,4-oxadiazole, **6d** (CAS: 89804-66-0)

**6d** was synthesized according to the general procedure, with Xantphos as ligand, and isolated by flash chromatography (silica gel, 0–30% EtOAc in isohexane) as an off-white amorphous solid (58 mg, 40%). ^1^H NMR (400 MHz, CDCl_3_) δ 8.36 (d, *J* = 8.2 Hz, 2H), 8.21–8.16 (m, 2H), 7.84 (d, *J* = 8.2 Hz, 2H), 7.56–7.50 (m, 3H). ^13^C{^1^H} NMR (101 MHz, CDCl_3_) δ 174.5, 169.4, 134.4 (d, *J* = 33.2 Hz), 131.6, 129.1, 128.7, 127.7, 127.6, 126.7, 126.3 (q, *J* = 3.8 Hz), 125.0 (d, *J* = 271.6 Hz). ^19^F NMR (376 MHz, CDCl_3_) δ −63.2. HRMS (ESI-TOF) *m*/*z*: [M + H]^+^ Calcd for C_15_H_10_F_3_N_2_O 354.0830; Found 354.0841.

#### 5-(3,4-Difluorophenyl)-3-(6-methylpyridin-3-yl)-1,2,4-oxadiazol,^23^ DDO-7263

DDO-7263 was synthesized according to the general procedure, with Xantphos as ligand, and isolated by flash chromatography (silica gel, 0–30% EtOAc in isohexane) as an off-white amorphous solid (51 mg, 37%). ^1^H NMR (400 MHz, CDCl_3_) δ 9.24 (dd, *J* = 2.2, 0.9 Hz, 1H), 8.29 (dd, *J* = 8.1, 2.2 Hz, 1H), 8.10–7.97 (m, 2H), 7.36 (ddd, *J* = 9.7, 8.6, 7.7 Hz, 1H), 7.33–7.29 (m, 1H), 2.65 (s, 3H). ^13^C{^1^H} NMR (101 MHz, CDCl_3_) δ 174.3–174.2 (m), 167.5, 161.9, 153.6 (dd, *J*_CF_ = 257.5, 12.7 Hz), 150.4 (dd, *J*_CF_ = 251.5, 13.1 Hz), 148.3, 135.1, 125.3 (dd, *J*_CF_ = 7.3, 3.9 Hz), 123.5, 121.2–121.1 (m), 120.2, 118.7 (d, *J*_CF_ = 18.2 Hz), 117.8 (dd, *J*_CF_ = 19.5, 1.6 Hz), 24.8. One carbon missing. ^19^F NMR (376 MHz, CDCl_3_) δ −128.7 to −129.7 (m), −134.4 to −135.2 (m). HRMS (ESI-TOF) *m*/*z*: [M + H]^+^ Calcd for C_14_H_10_F_2_N_3_O 274.0792; Found 274.0791.

#### 5-(2-Fluorophenyl)-3-(*m*-tolyl)-1,2,4-oxadiazole^6^ Ataluren Precursor

Ataluren precursor was synthesized according to the general procedure, with Xantphos as ligand, and isolated by flash chromatography (silica gel, 0–30% EtOAc in isohexane) as an off-white amorphous solid (66 mg, 52%). ^1^H NMR (400 MHz, CDCl_3_) δ 8.25–8.20 (m, 1H), 8.01 (s, 1H), 8.01–7.96 (m, 1H), 7.64–7.57 (m, 1H), 7.44–7.37 (m, 1H), 7.37–7.32 (m, 2H), 7.32–7.26 (m, 1H), 2.45 (s, 3H). ^13^C{^1^H} NMR (101 MHz, CDCl_3_) δ 172.8 (d, *J*_CF_ = 4.5 Hz), 169.0, 160.9 (d, *J*_CF_ = 260.5 Hz), 138.8, 134.7 (d, *J*_CF_ = 8.6), 132.2, 131.1, 128.9, 128.3, 126.7, 124.9, 124.8 (d, *J*_CF_ = 3.7 Hz), 117.3 (d, *J*_CF_ = 21.0 Hz), 113.1 (d, *J*_CF_ = 11.4 Hz), 21.5. ^19^F NMR (376 MHz, CDCl_3_) δ −108.3 to −108.4 (m). HRMS (ESI-TOF) *m*/*z*: [M + H]^+^ Calcd for C_15_H_12_FN_2_O 255.0934; Found 255.0938.

### General Procedure for Synthesis of [Carbonyl-^11^C]acyl Amidines

Aryl iodide (9 μmol), benzamidine (2 equiv), and Pd(PPh_3_)_4_ (0.1 equiv) were transferred to a 950 μL oven-dried conical vial and dissolved in 1,4-dioxane (400 μL). After capping the vial, the reaction mixture was sonicated before addition of triethylamine (4.0 equiv). Following purging with N_2_, the vial was placed in the xenon system. The [^11^C]CO_2_ produced in the cyclotron was transferred to the xenon system in a stream of helium gas and concentrated on a CO_2_ trap immersed in liquid nitrogen.^42,43^ Heating of the trap released [^11^C]CO_2_, which was reduced to [^11^C]CO over zinc heated to 400 °C. Residual [^11^C]CO_2_ was trapped on an Ascarite column, and [^11^C]CO was concentrated on a CO trap immersed in liquid nitrogen. Before heating the trap, the carrier gas was changed from helium to xenon (>99.9%, 1.5 mL/min). The concentrated [^11^C]CO was transferred to the capped reaction vial through a transfer needle.

After collection of [^11^C]CO, the radioactivity was measured to determine the starting amount of [^11^C]CO (A1). The reaction was heated at 120 °C for 10 min. Upon reaction completion, the radioactivity was measured a second time (A2) before the reaction vial was vented and purged with N_2_ to remove unreacted [^11^C]CO and any volatile ^11^C-labeled compounds formed under the course of the reaction. A third radioactivity measurement (A3) was performed before semipreparative HPLC purification. Lastly, a final radioactivity measurement of the isolated ^11^C product fraction was performed (A4).

The [^11^C]CO conversion was based on the radioactivity measurements A2 and A3, with A3 decay corrected to the time of A2 measurement. After the isolation and final radioactivity measurement (A4), an aliquot was analyzed to determine the radiochemical purity and the identity of the ^11^C-labeled product. The isotopically unmodified product was used as reference. The reported isolated radiochemical yields are decay corrected to time of A1 measurement and based on the ^11^C-labeled product activity (A4) and the amount of [^11^C]CO collected in the reaction vial (A1).

#### [Carbonyl-^11^C]*N*-(imino(phenyl)methyl)-4-methoxybenzamide, ^**11**^**C-3f** (CAS: 1445133-92-5)

^**11**^**C-3f** was synthesized according to the general procedure (4 experiments). Purification method: 0–50% acetonitrile followed by 100% acetonitrile, total run time 25 min, flow 5 mL/min. Analytical method: (system A) 10–90% acetonitrile (10 min) followed by 100% acetonitrile, total run time 15 min, flow 2 mL/min (system B) 35% acetonitrile (10 min) followed by 100% acetonitrile, total run time 15 min, flow 2 mL/min. Exp 1: Starting from 5.0 GBq, 0.45 GBq was isolated at 45 min from EOB. Exp 2: Starting from 3.5 GBq, 0.19 GBq was isolated at 41 min from EOB. Exp 3: Starting from 7.4 GBq, 0.40 GBq was isolated at 44 min from EOB. Exp 4: 5 min reaction time. Starting from 3.2 GBq, 0.083 GBq was isolated at 37 min from EOB. Analytical HPLC *R*_t_ = 3.5 min (system A); *R*_t_ = 8.2 min (system B).

#### [Carbonyl-^11^C]4-acetyl-*N*-(imino(phenyl)methyl)benzamide, ^**11**^**C-3b**

^**11**^**C-3b** was synthesized according to the general procedure (4 experiments). Purification method: 5–50% acetonitrile followed by 100% acetonitrile, total run time 25 min, flow 5 mL/min. Analytical method: same as above. Exp 1: Starting from 5.0 GBq, 0.56 GBq was isolated at 38 min from EOB. Exp 2: Starting from 4.3 GBq, 0.38 GBq was isolated at 40 min from EOB. Exp 3: Starting from 15.4 GBq, 2.1 GBq was isolated at 38 min from EOB. Exp 4: Starting from 14.3 GBq, 1.7 GBq was isolated at 38 min from EOB. Analytical HPLC *R*_t_ = 4.4 min (system A); *R*_t_ = 4.6 min (system B).

#### Molar Activity Determination of ^**11**^**C-3b**

The molar activity was determined in experiments 3 and 4. From an aliquot of the isolated ^11^C-product fraction, 50 μL was injected and analyzed with system B at 254 nm. The molar activity was calculated with the equation derived from the calibration curve (see Supporting Information).

#### Synthesis of [Carbonyl-^11^C]3-phenyl-5-(*p*-tolyl)-1,2,4-oxadiazole, ^**11**^**C-6a**

Hydroxylamine hydrochloride (7.0 equiv) was dissolved in 150 μL of 50% acetic acid (aq). Intermediate [carbonyl-^11^C]*N*-(imino(phenyl)methyl)-4-methylbenzamide was synthesized as above except for a 5 min reaction time. The hydroxylamine hydrochloride solution was added to the reaction mixture and heated for another 5 min at 150 °C. Purification method: 60% acetonitrile followed by 100% acetonitrile, total run time 25 min, flow 5 mL/min. Analytical method: (system A) 10–90% acetonitrile (10 min) followed by 100% acetonitrile, total run time 15 min, flow 2 mL/min (system B) 10–90% acetonitrile (10 min) followed by 100% acetonitrile, total run time 15 min, flow 2 mL/min. Exp 1: Starting from 5.1 GBq, 0.34 GBq was isolated at 43 min from EOB. Exp 2: Starting from 3.6 GBq, 0.32 GBq was isolated at 39 min from EOB. Analytical HPLC *R*_t_ = 9.7 min (system A); *R*_t_ = 8.9 min (system B).

## Supporting Information Available

Calculations and definitions regarding the ^11^C chemistry, NMR spectra (^1^H NMR, ^13^C{^1^H} NMR) of compounds **3a**–**v**, and HPLC chromatograms of ^11^C-labeled compounds **3b**, **3f**, and **6a** (PDF)
