# Supplementary material for: Acyl Amidines by Pd-Catalyzed Aminocarbonylation: One-Pot Cyclizations and 11C Labeling
Source: J Org Chem. 2022 Dec 15;88(8):5078–89. doi: 10.1021/acs.joc.2c02115 (PMC10127271; doi:10.1021/acs.joc.2c02115)
Supplement: Supplementary file 1 — jo2c02115_si_001.pdf [file jo2c02115_si_001.pdf]

Supporting information

Acyl amidines by Pd-catalyzed aminocarbonylation: one-pot cyclizations and ^{11}C-labeling

*Jonas Rydfjord, Sara Roslin, Tamal Roy, Alaa Abbas, Marc Y. Stevens, Luke R. Odell**

Department of Medicinal Chemistry, Uppsala University, Box-574, SE-751 23 Uppsala, Sweden

*Corresponding author: luke.odell@ilk.uu.se Telephone: +46 018-471 4297

Content

- Calculations and definitions for the ^{11}C-radiochemistry S2
- Calibration curve and molar activity determination S3
- Double chamber system, and CO releasing system S4
- NMR spectra S5
- HPLC-chromatogram S38

Calculations and definitions for the ^{11}C-radiochemistry

[^{11}C]CO-conversion

The [^{11}C]CO-conversion is the measurement of [^{11}C]CO incorporated into non-volatile compounds. It was calculated from the radioactivity measurements A2 and A3. The A3 measurement has been decay-corrected (d.c.) to the time-point of A2 measurement.

$$\text{Conversion} = \frac{\text{A3 (d.c.)}}{A2}$$

Product selectivity

The product selectivity is the percentage of formed ^{11}C-labeled product and is based on a HPLC analysis of the reaction mixture.

Radiochemical yield

The radiochemical yield (RCY) is based on the activity of the isolated ^{11}C-labeled (A4) and the starting radioactivity measurement (starting amount of [^{11}C]CO in the vial, A1). The A4 measurement has been decay-corrected to the time-point of A1 measurement.

$$RCY = \frac{A4\ (d.c.)}{A1}$$

An estimation of the radiochemical yield is calculated from the [^{11}C]CO-conversion and the product selectivity.

$$Estimated\ RCY = Conversion\ \times Product\ selectivity$$

Radiochemical purity

The radiochemical purity is analyzed with HPLC of an aliquot from the isolated ^{11}C-labeled product fraction.

Calibration curve and molar activity determination

A calibration curve for 4-Acetyl-*N*-(imino(phenyl)methyl)benzamide was prepared using five concentrations; 0.25, 0.5, 1.0, 2.0 and 5.0 µg/mL. 50 µL was injected on system B. Analysis was performed at 254 nm and a calibration curve was constructed (Figure S1). A blank sample consisting of acetonitrile was injected between every run to avoid carry-over.

The molar activity was determined with the results from experiment 3 and 4 and the equation derived from the calibration curve (Table S1).

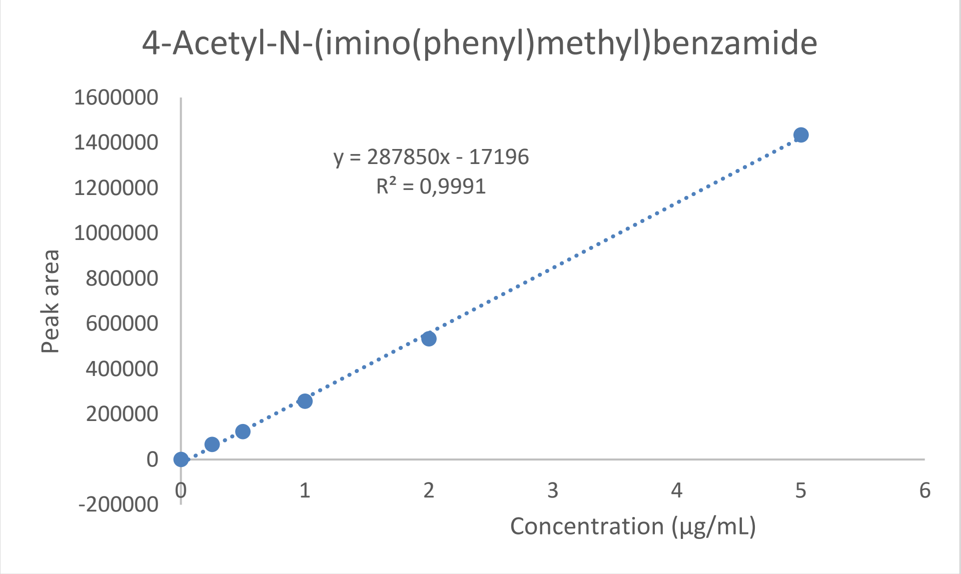

Figure S1. Calibration curve for 4-Acetyl-*N*-(imino(phenyl)methyl)benzamide.

Table S1. Determination of molar activity.

Experiment	Area	Concentration (µg/mL)	Volume (mL)	Mass (µg)	Amount (µmol)	Activity (GBq)	Molar activity (GBq/µmol)
**1**	75335	0.32	2.7	0.87	0.0033	2.1	650
**2**	89132	0.37	2.5	0.91	0.0034	1.7	488

Double chamber system and CO releasing system

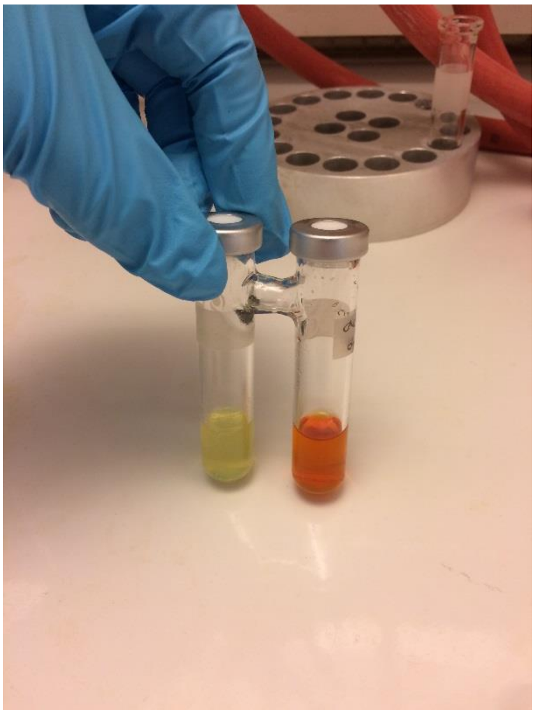

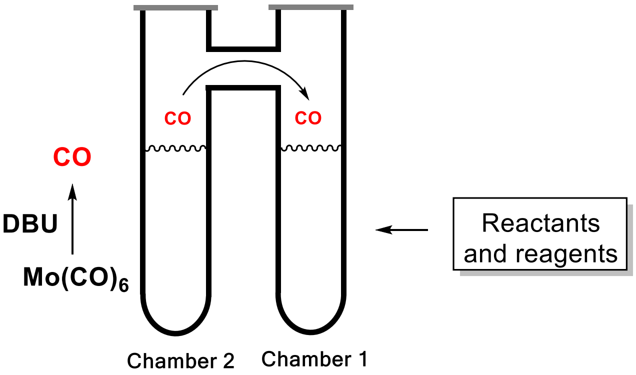

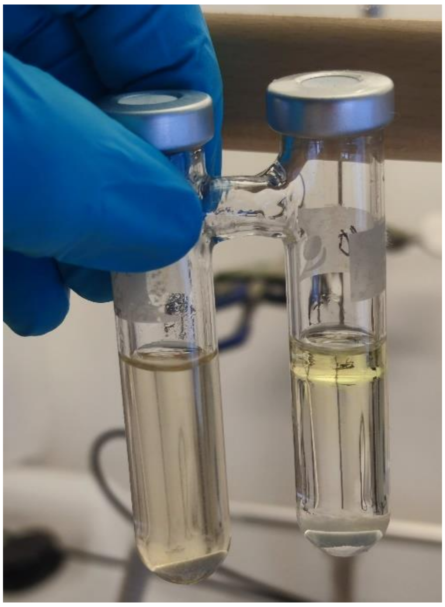

Slow mixing of DBU and 1,4-dioxane allows for capping before release of carbon monoxide.

NMR spectra

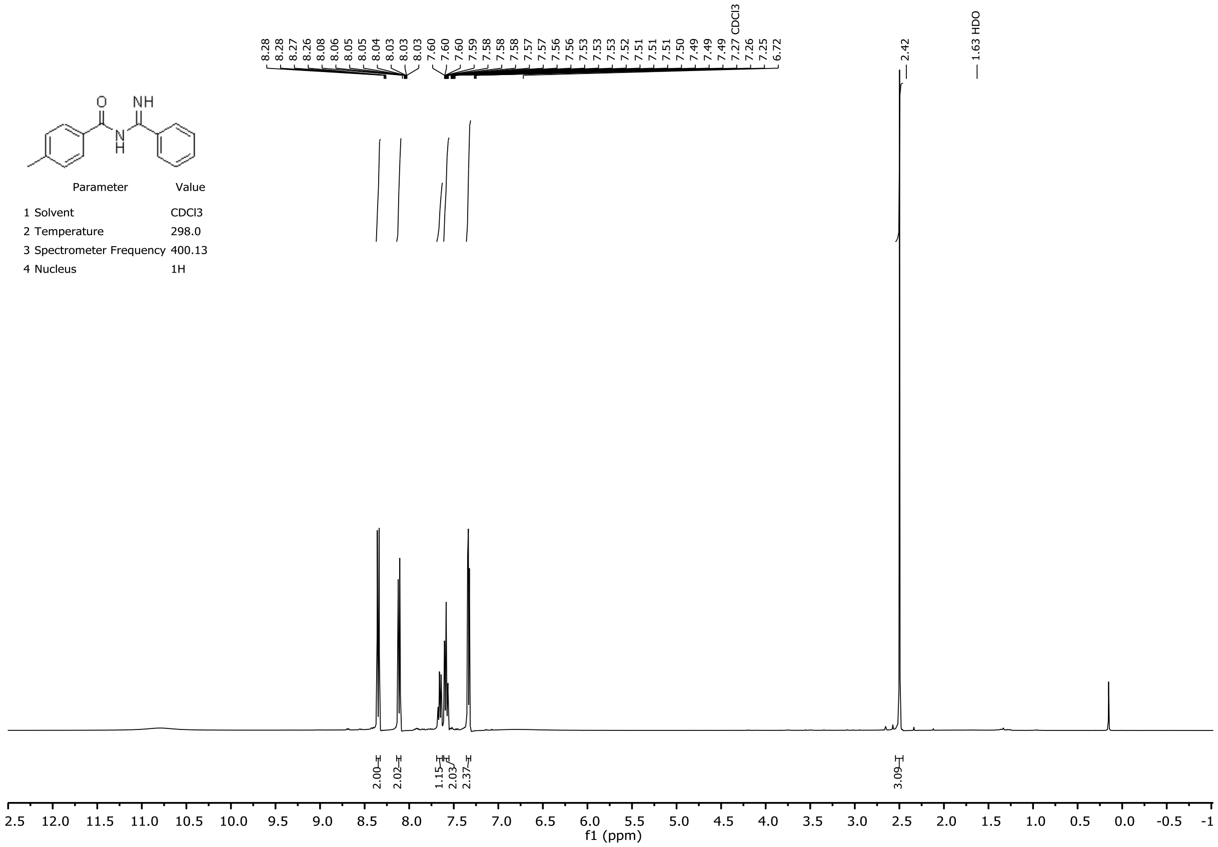

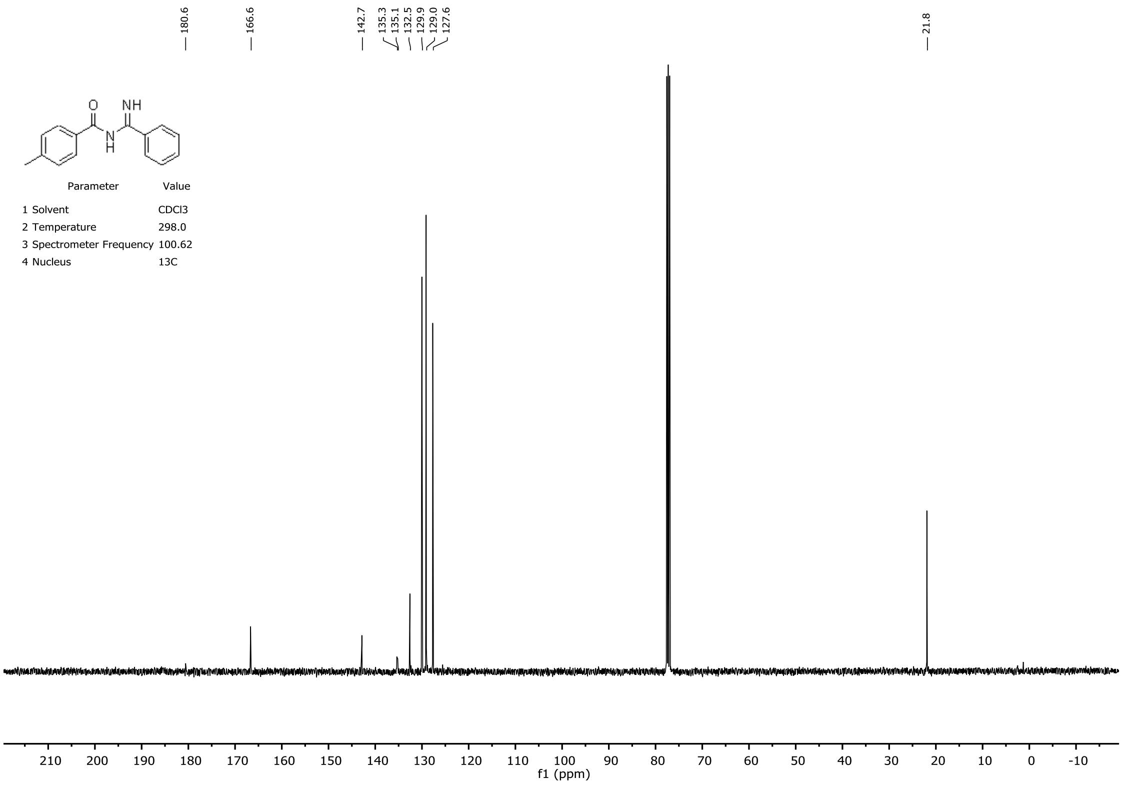

N-(Imino(phenyl)methyl)-4-methylbenzamide ***3a*** *CAS: 68167-55-5*

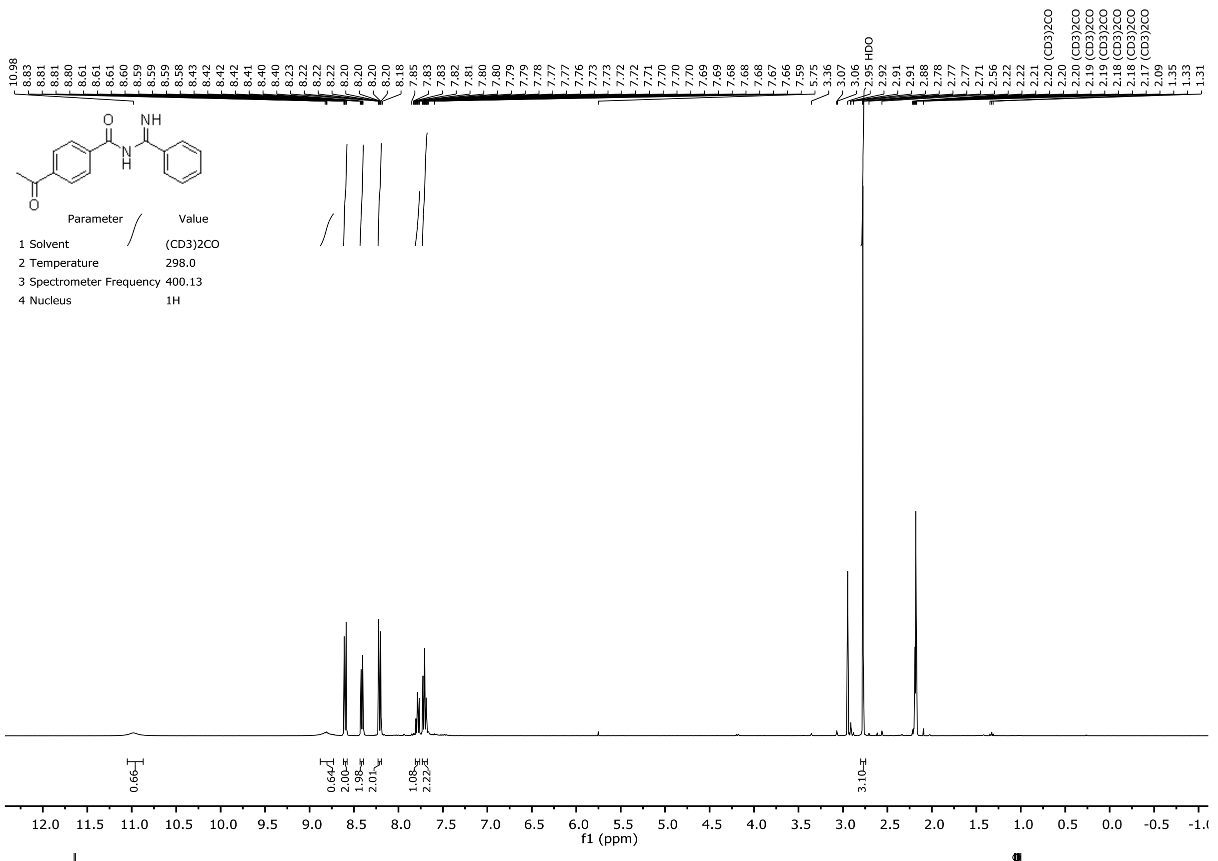

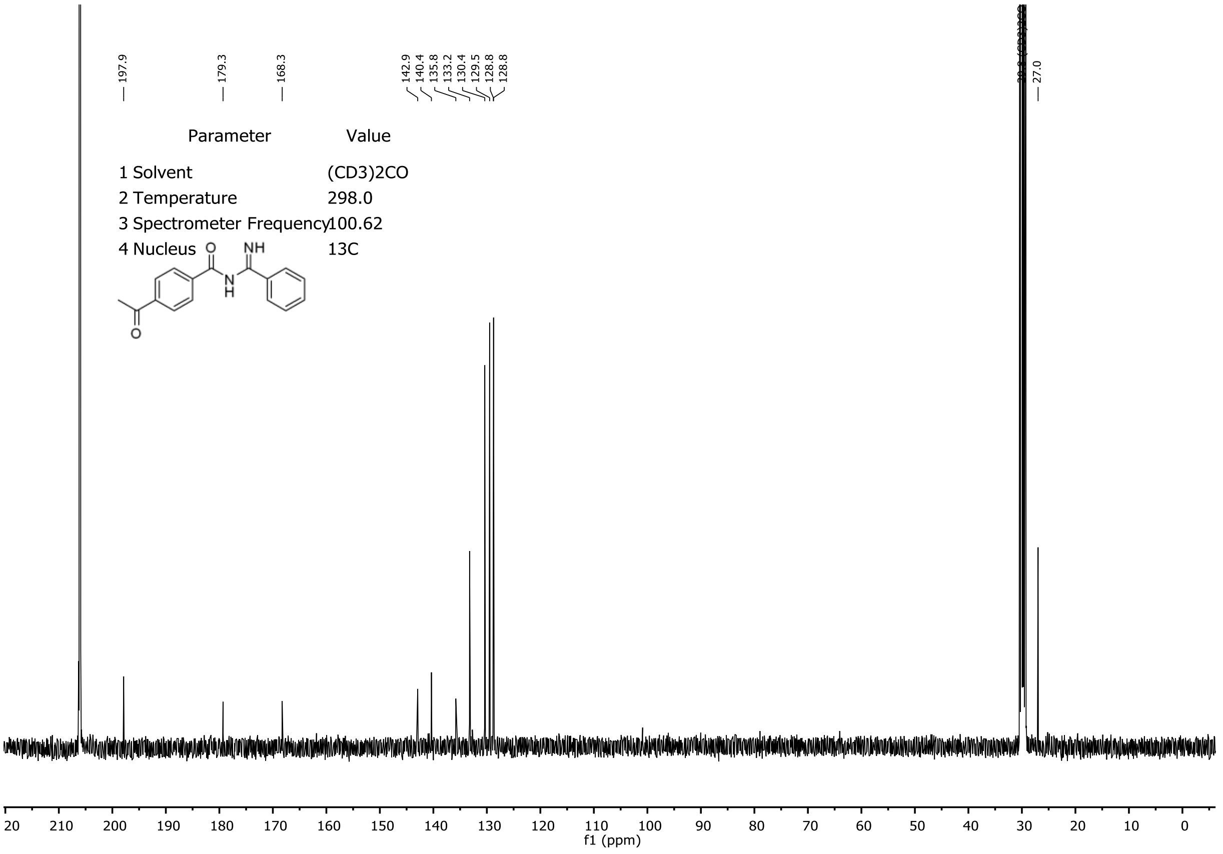

4-Acetyl-N-(imino(phenyl)methyl)benzamide ***3b*** *CAS: -*

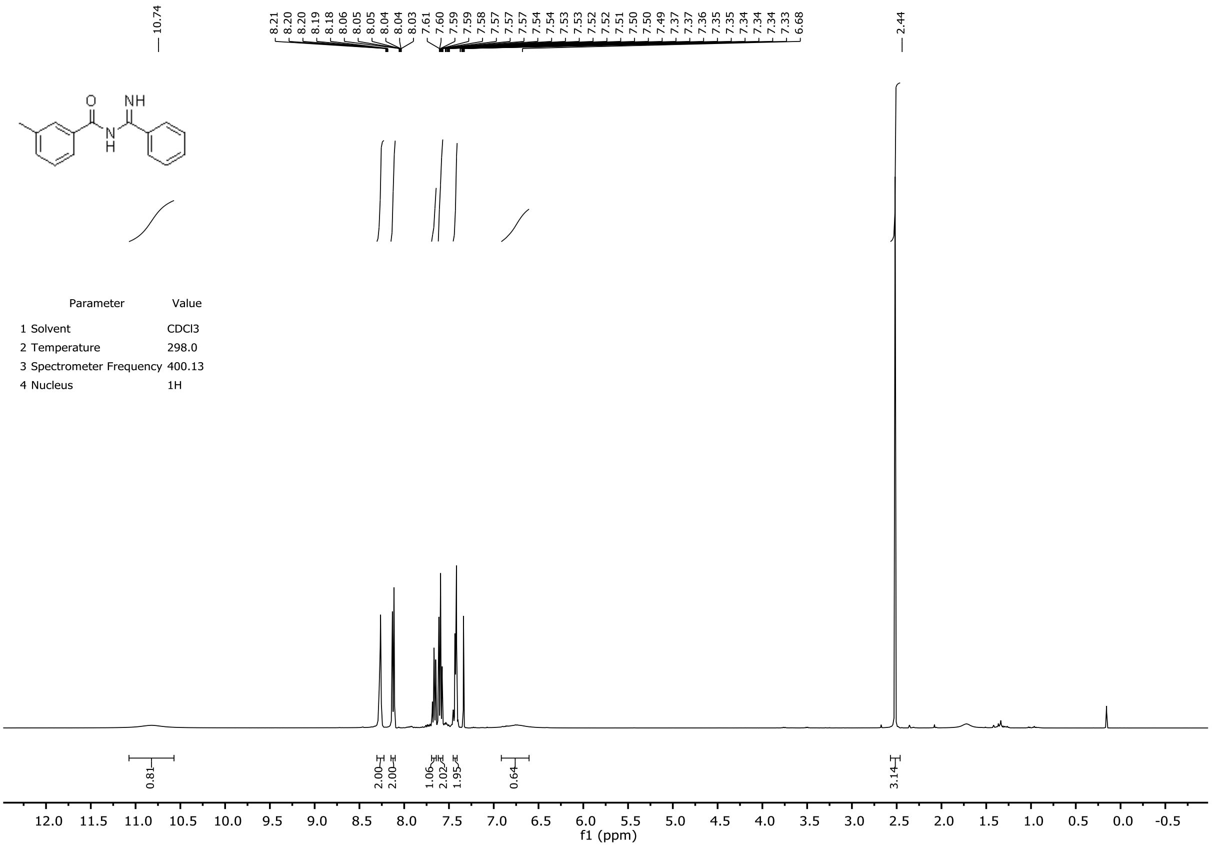

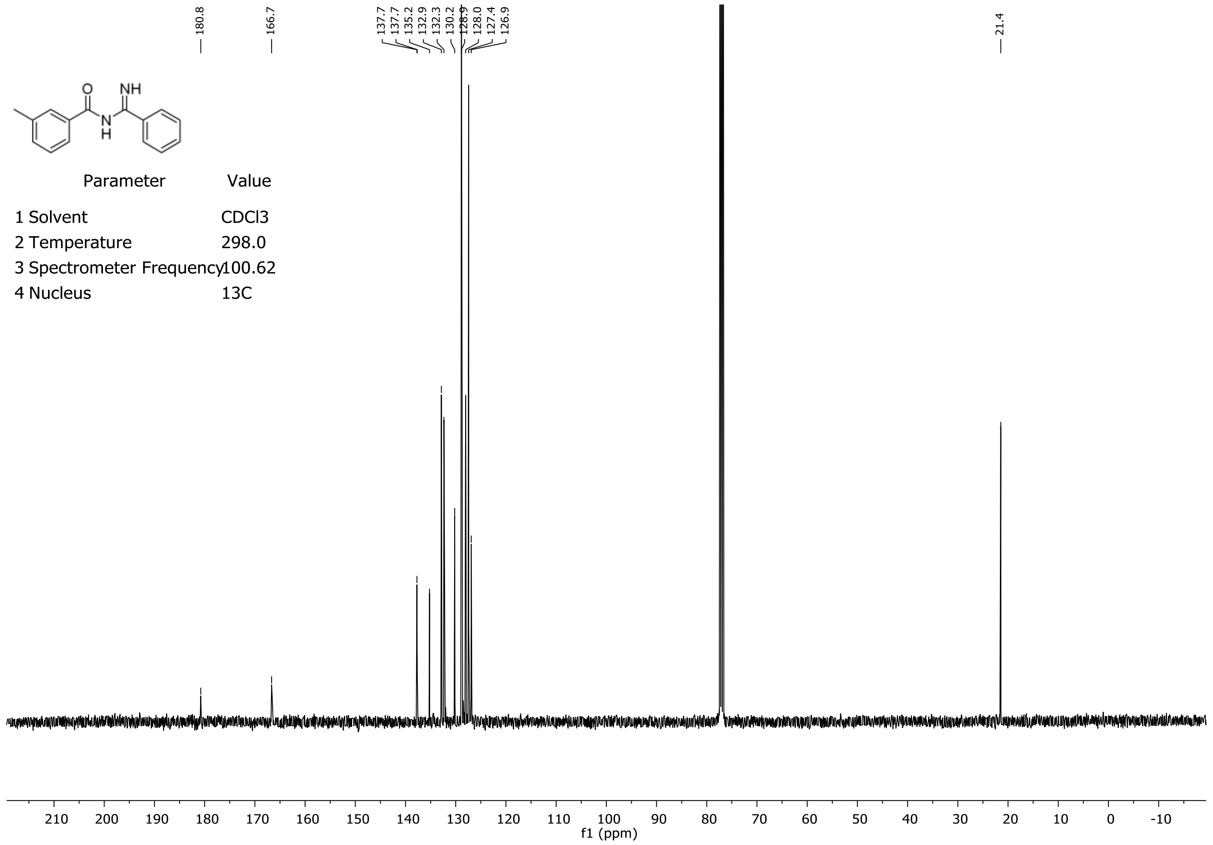

N-(imino(phenyl)methyl)-3-methylbenzamide ***3c*** *CAS: -*

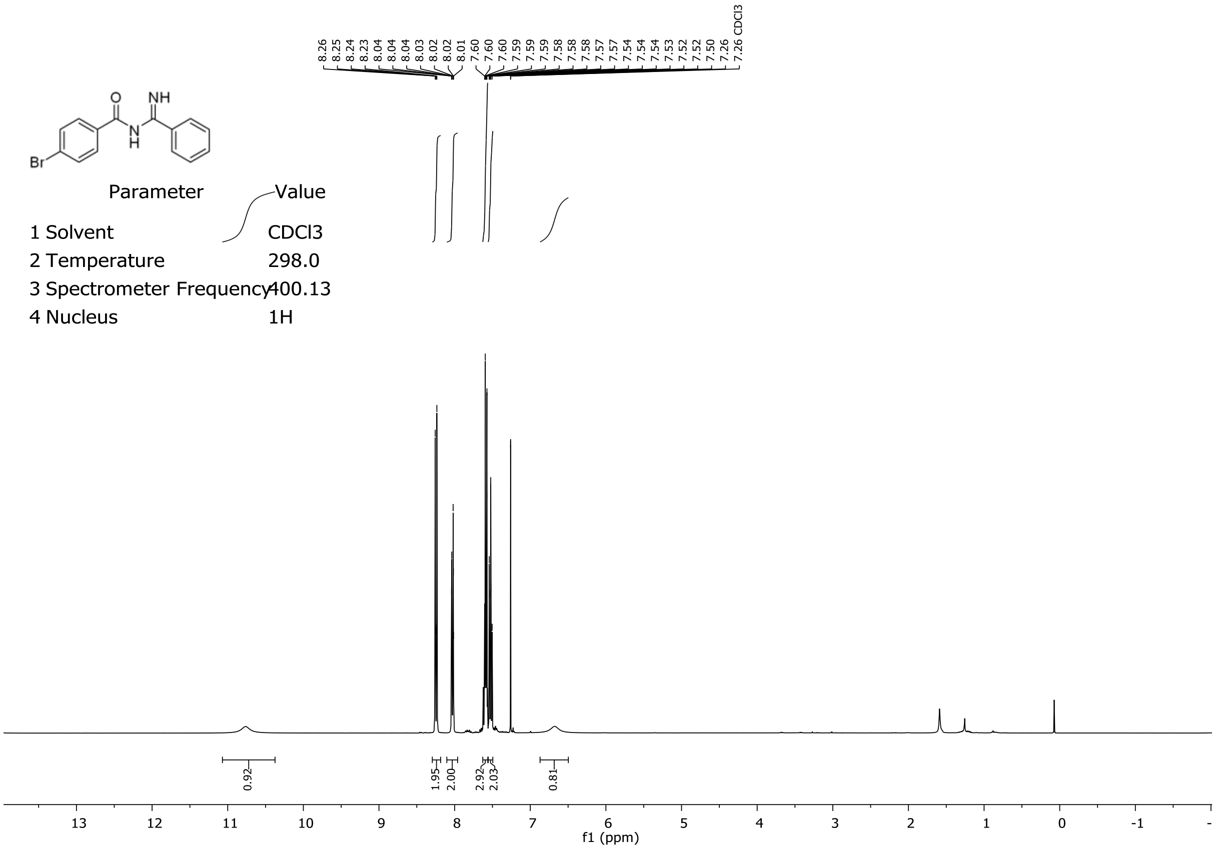

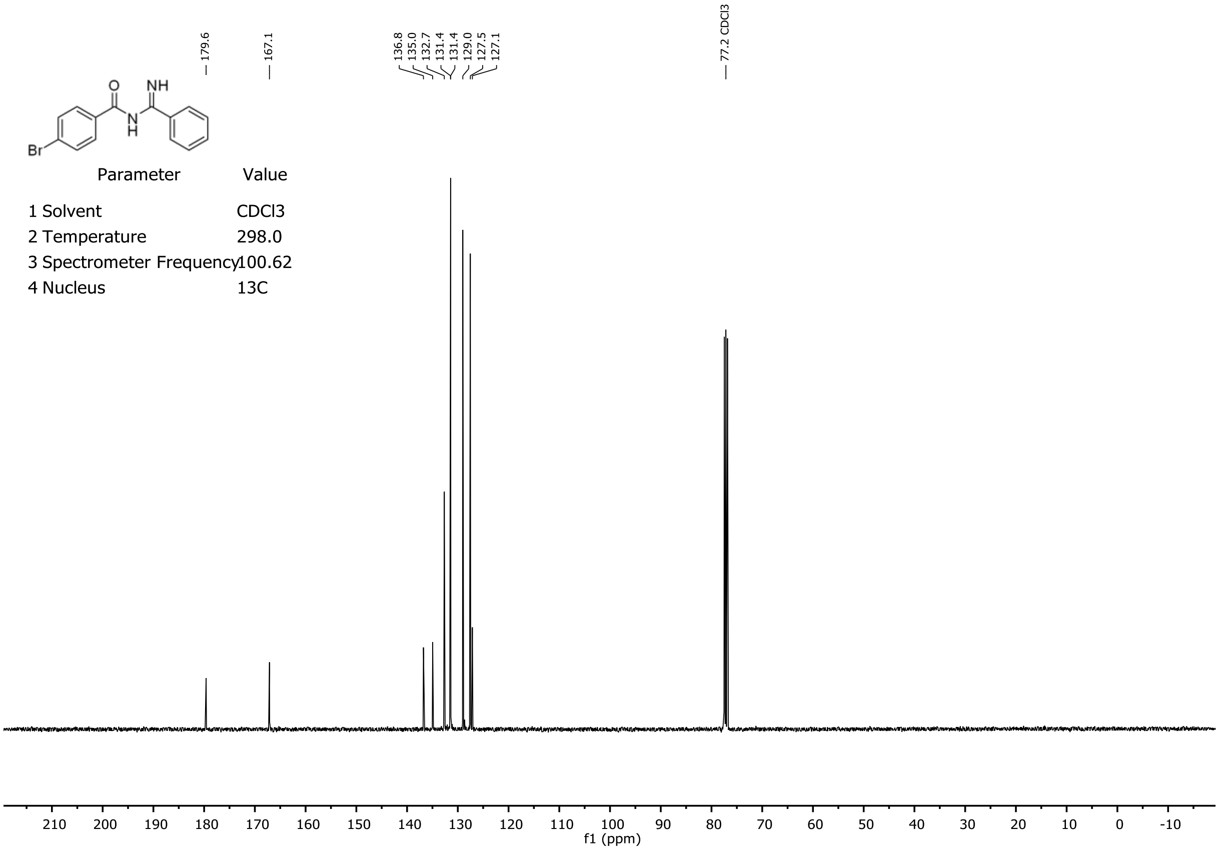

4-Bromo-N-(imino(phenyl)methyl)benzamide ***3d*** *CAS: 68167-57-7*

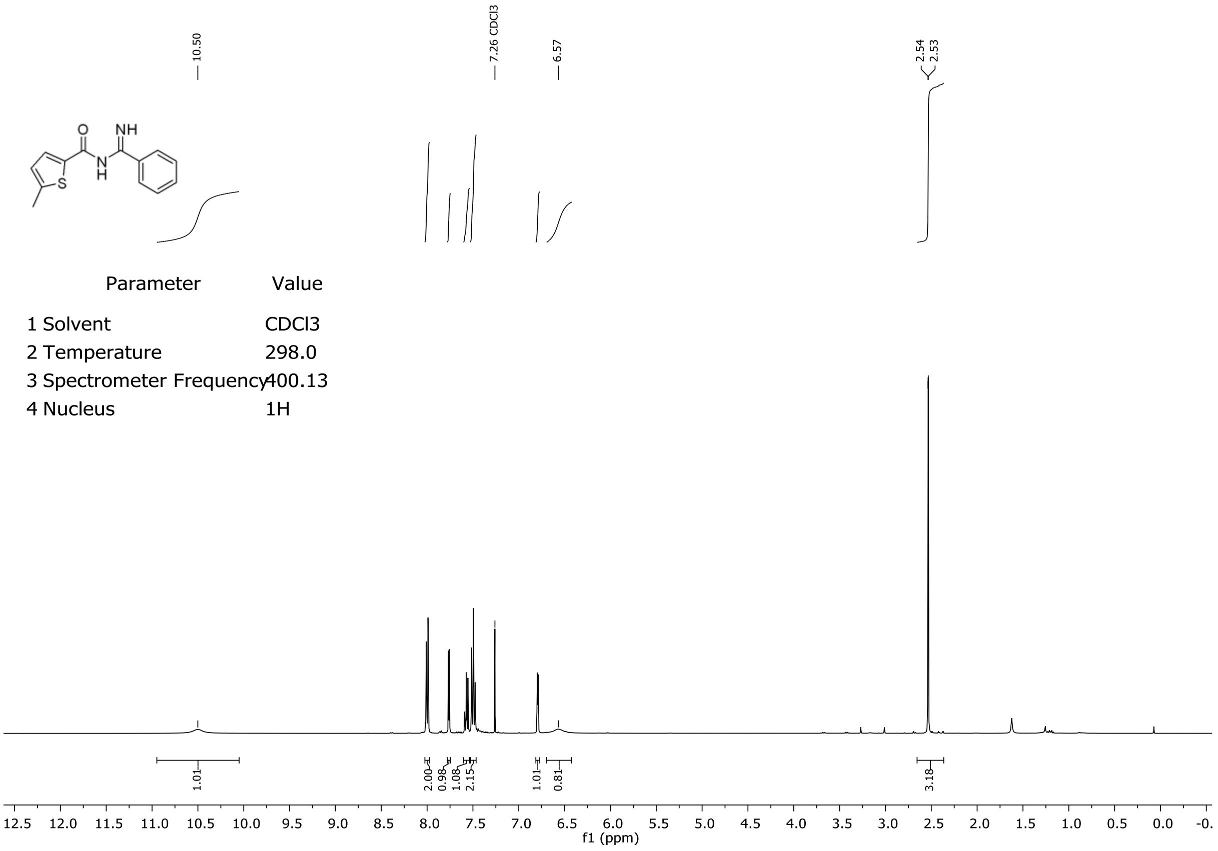

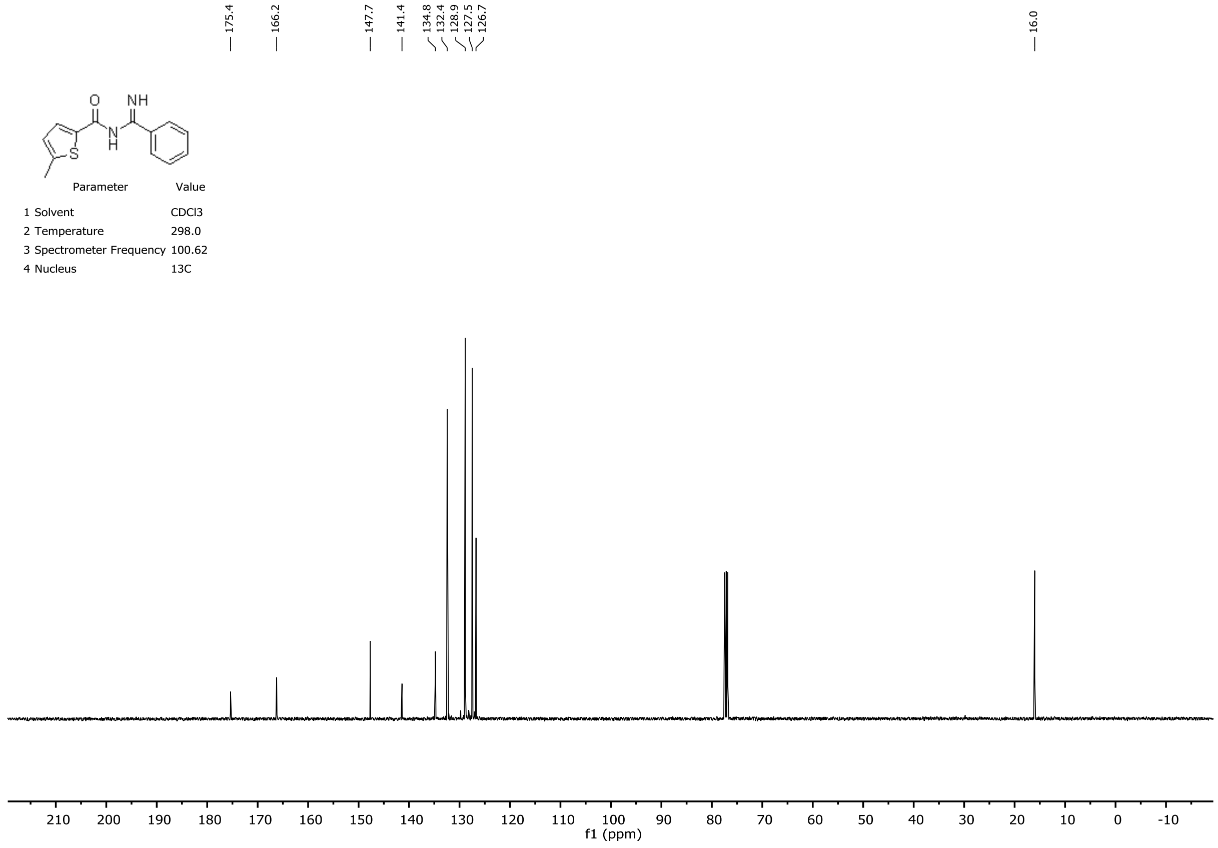

N-(Imino(phenyl)methyl)-5-methylthiophene-2-carboxamide ***3e*** *CAS: -*

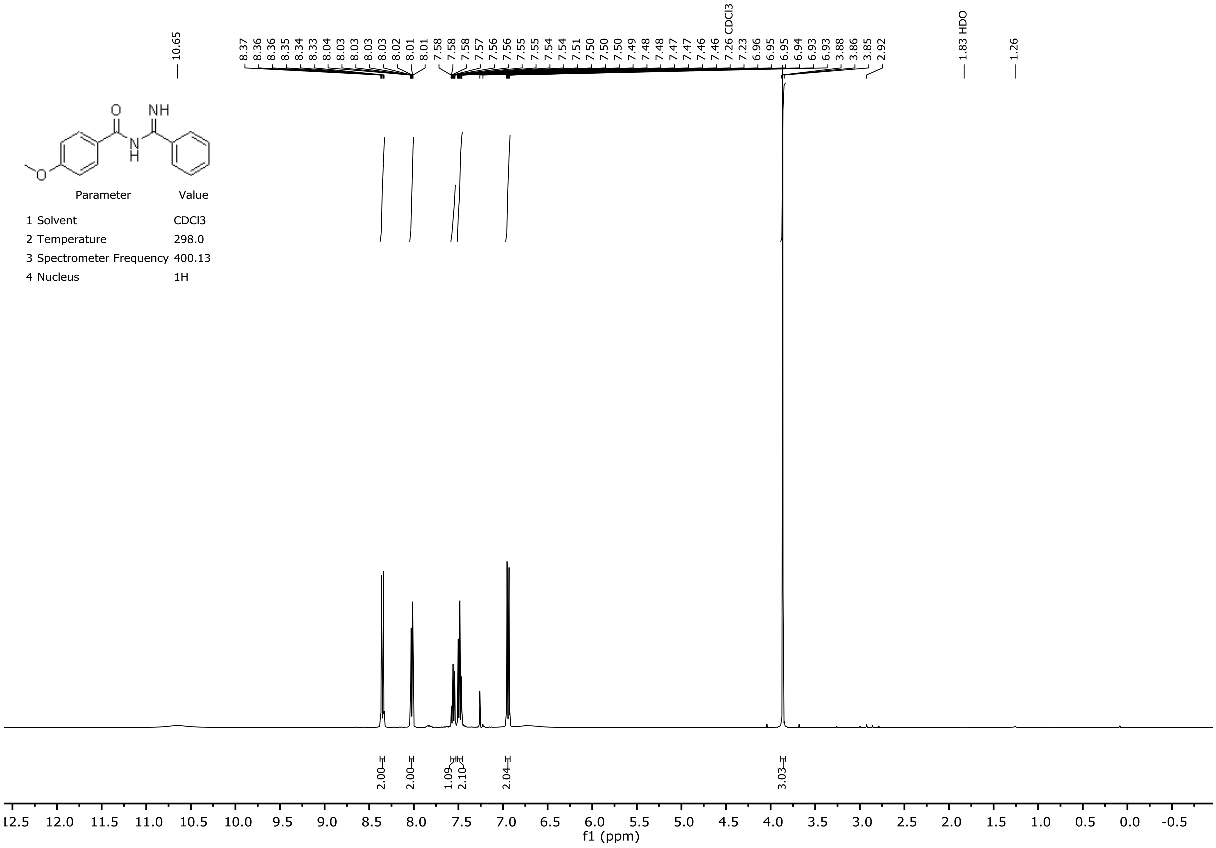

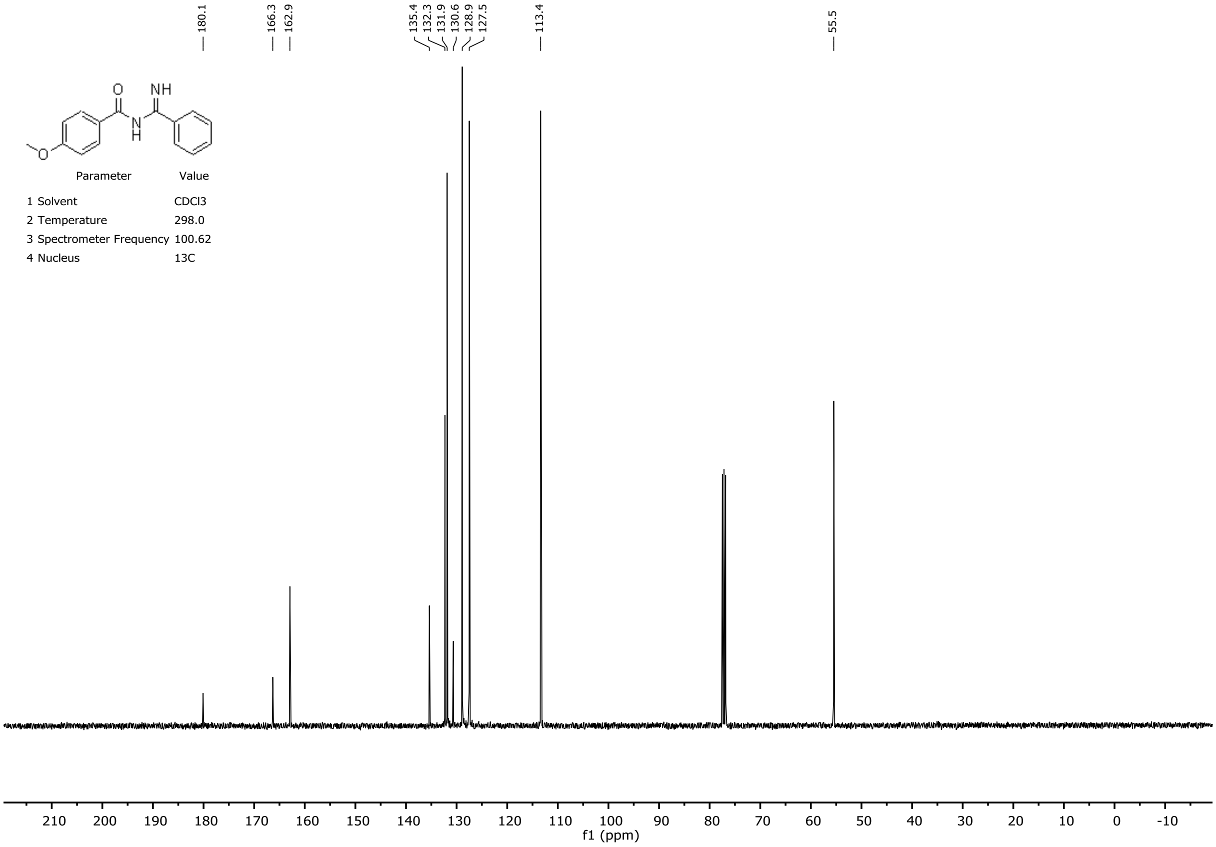

*N-(Imino(phenyl)methyl)-4-methoxybenzamide **3f** CAS: 1445133-92-5*

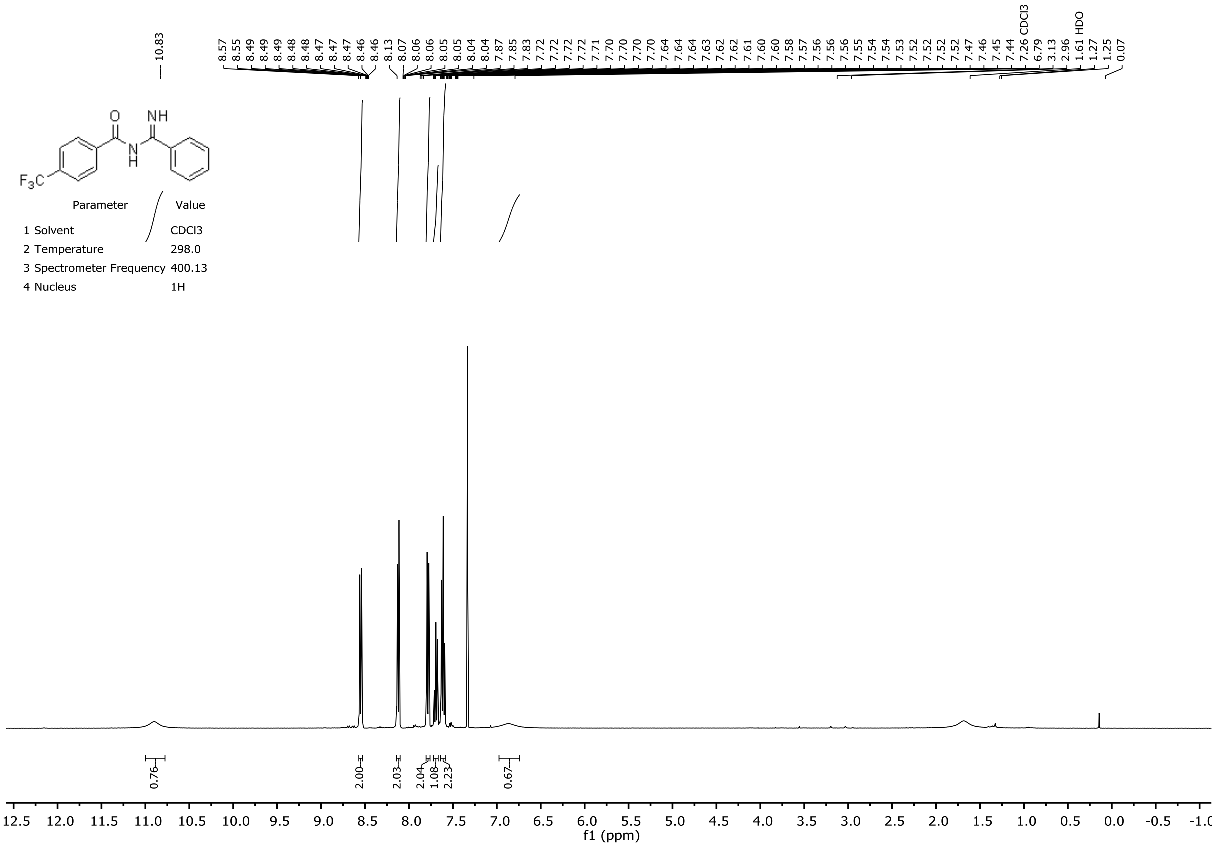

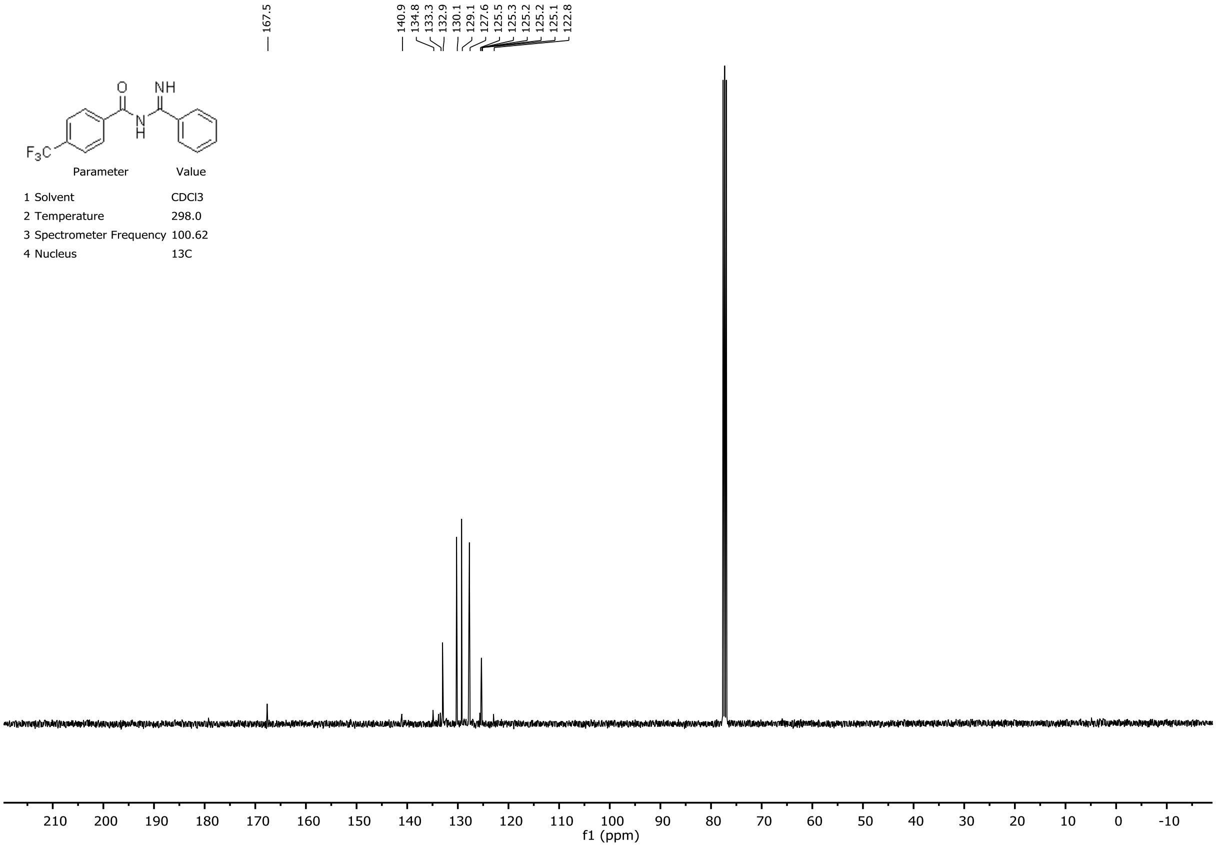

*N-(Imino(phenyl)methyl)-4-(trifluoromethyl)benzamide **3g** CAS: 2052280-77-8*

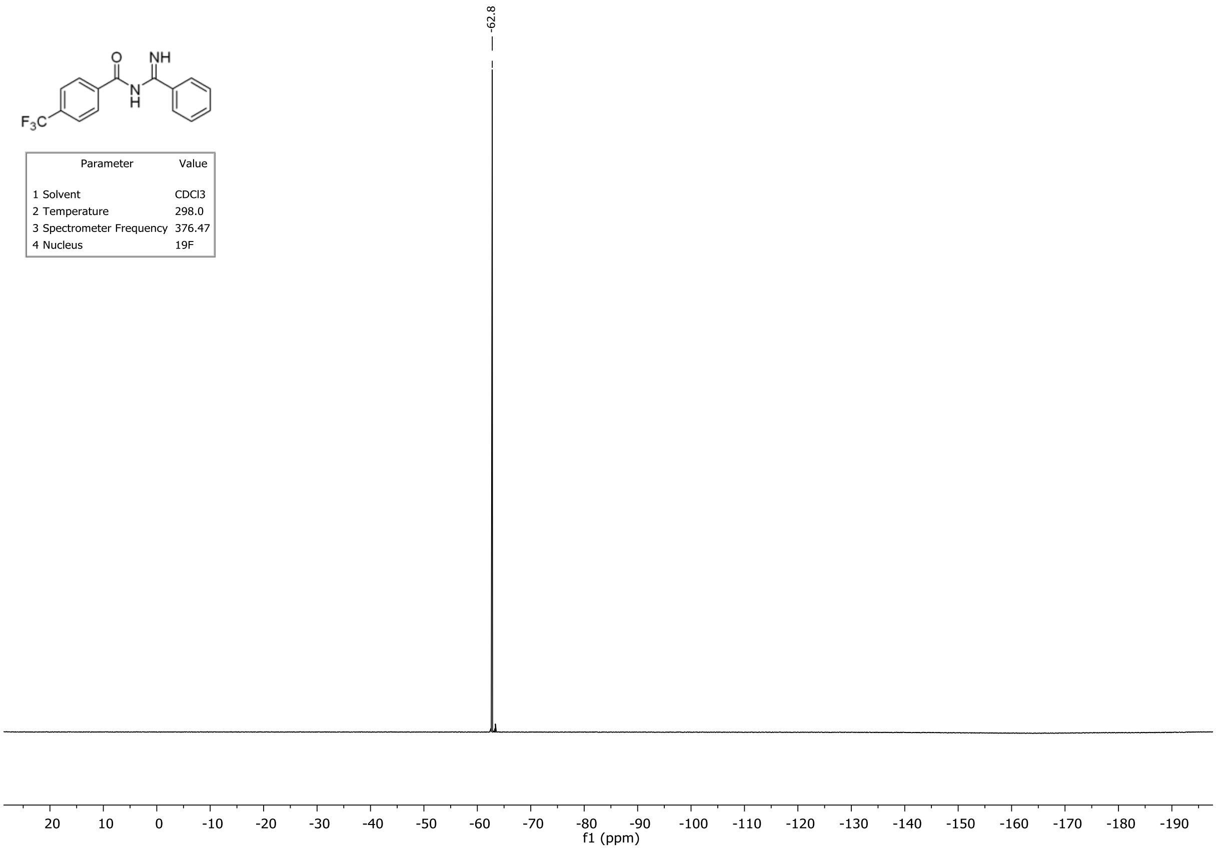

N-(Imino(phenyl)methyl)-4-(trifluoromethyl)benzamide ***3g*** *CAS: 2052280-77-8*

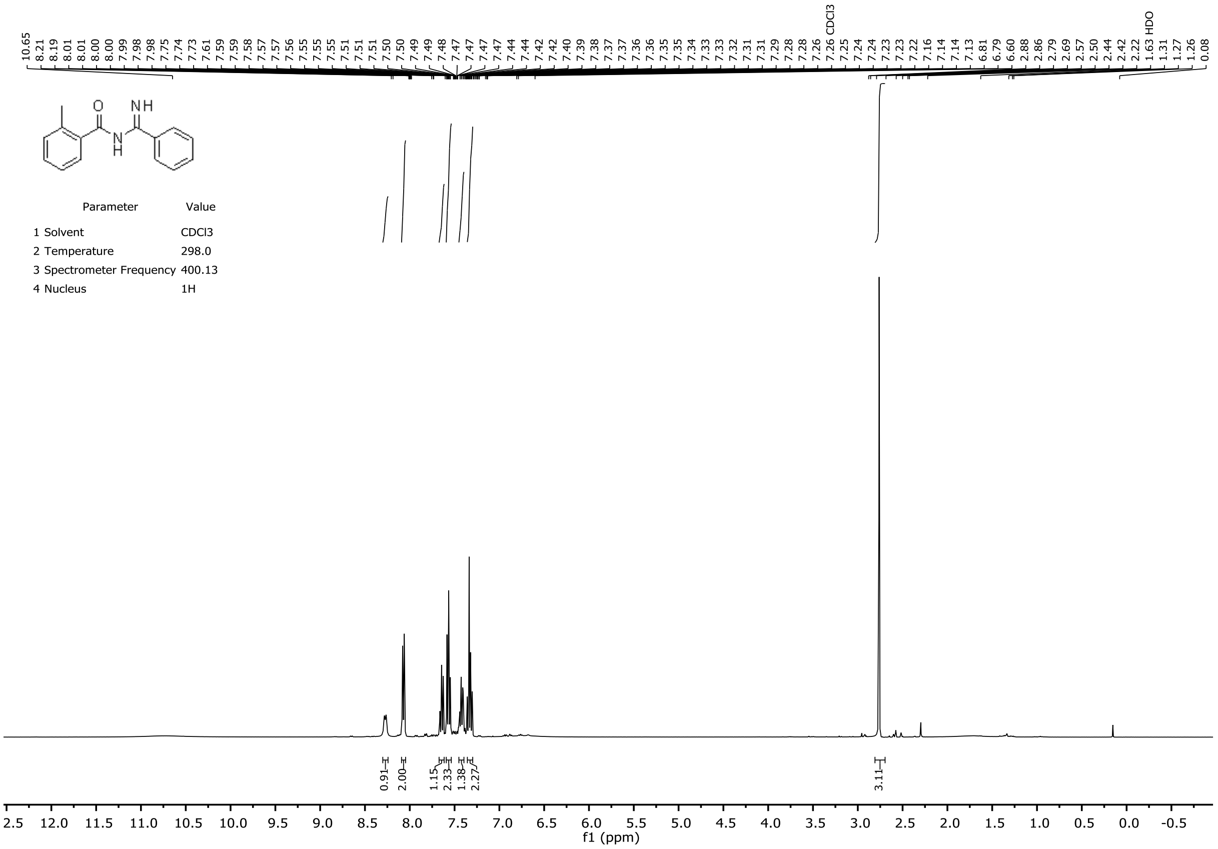

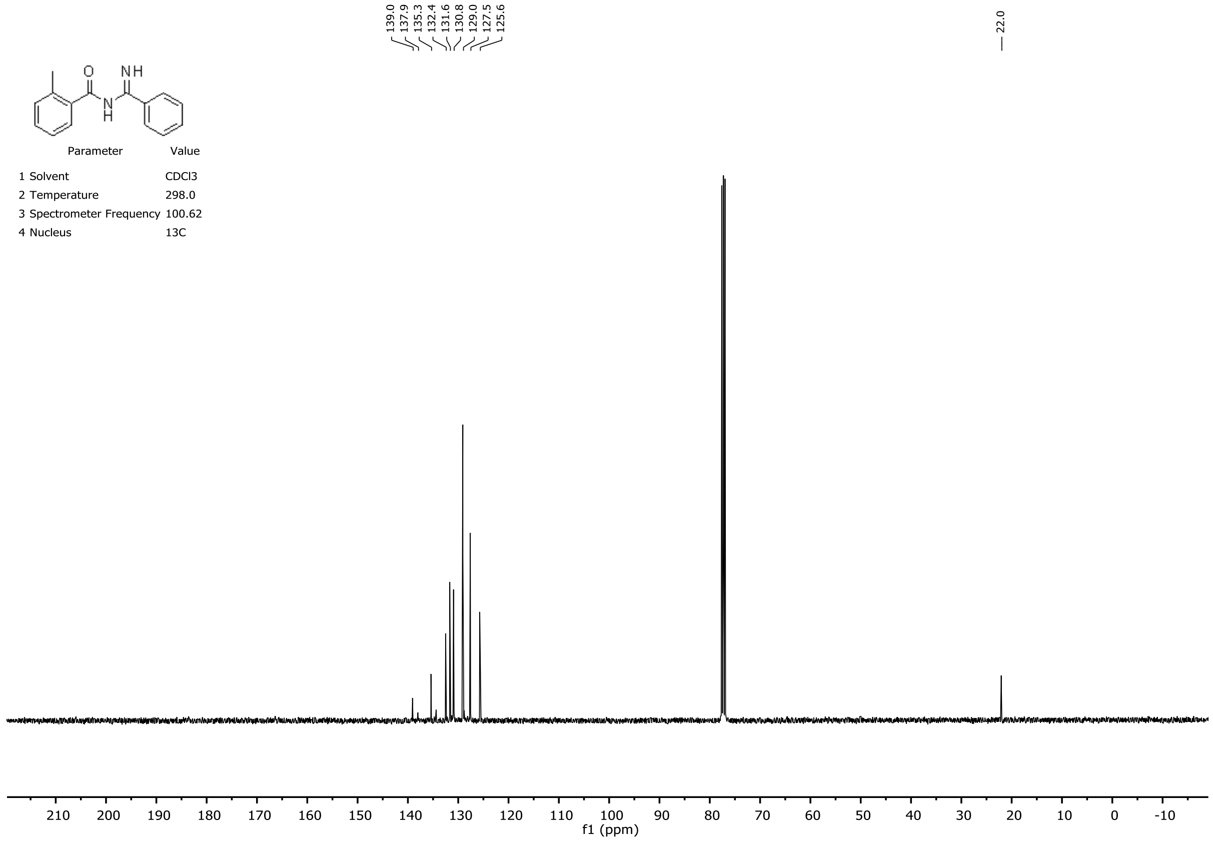

N-(Imino(phenyl)methyl)-2-methylbenzamide ***3h*** *CAS: 872266-80-3*

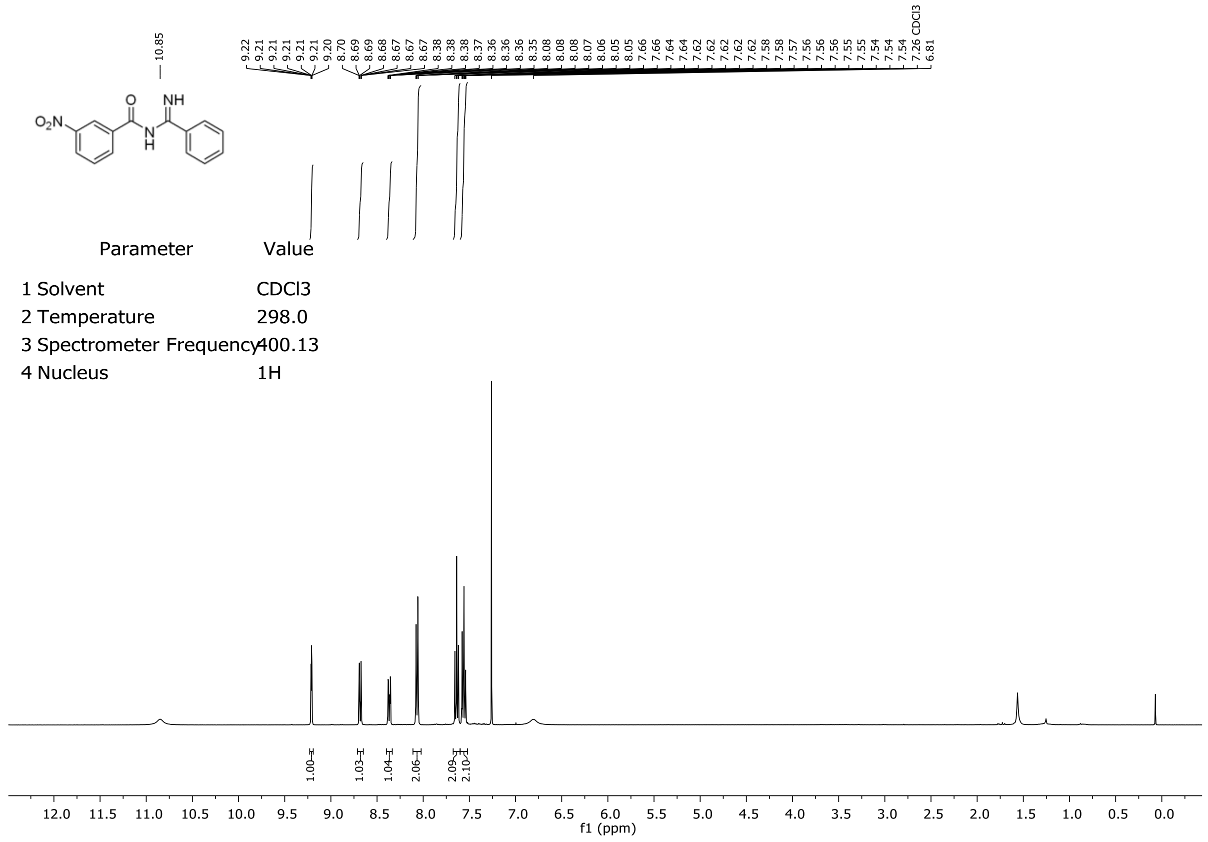

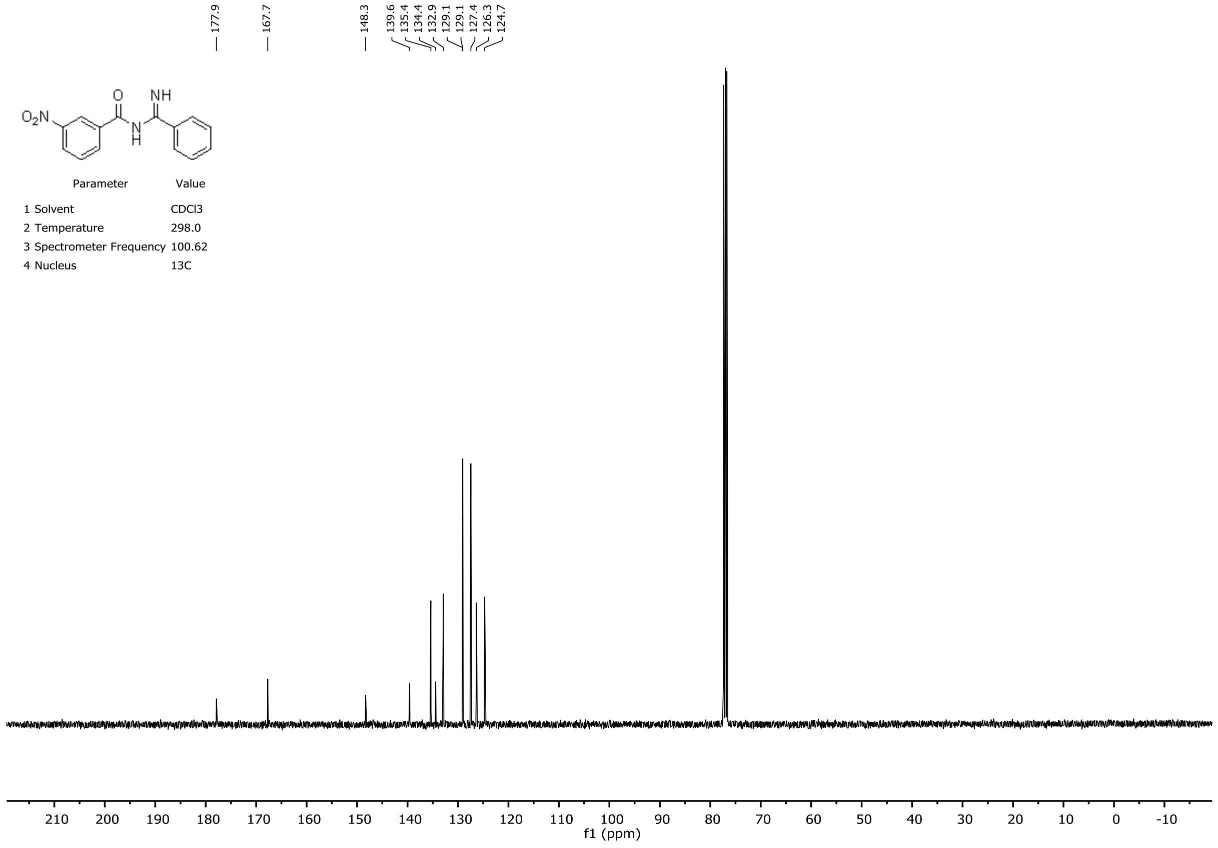

*N-(Imino(phenyl)methyl)-3-nitrobenzamide **3i** CAS: -*

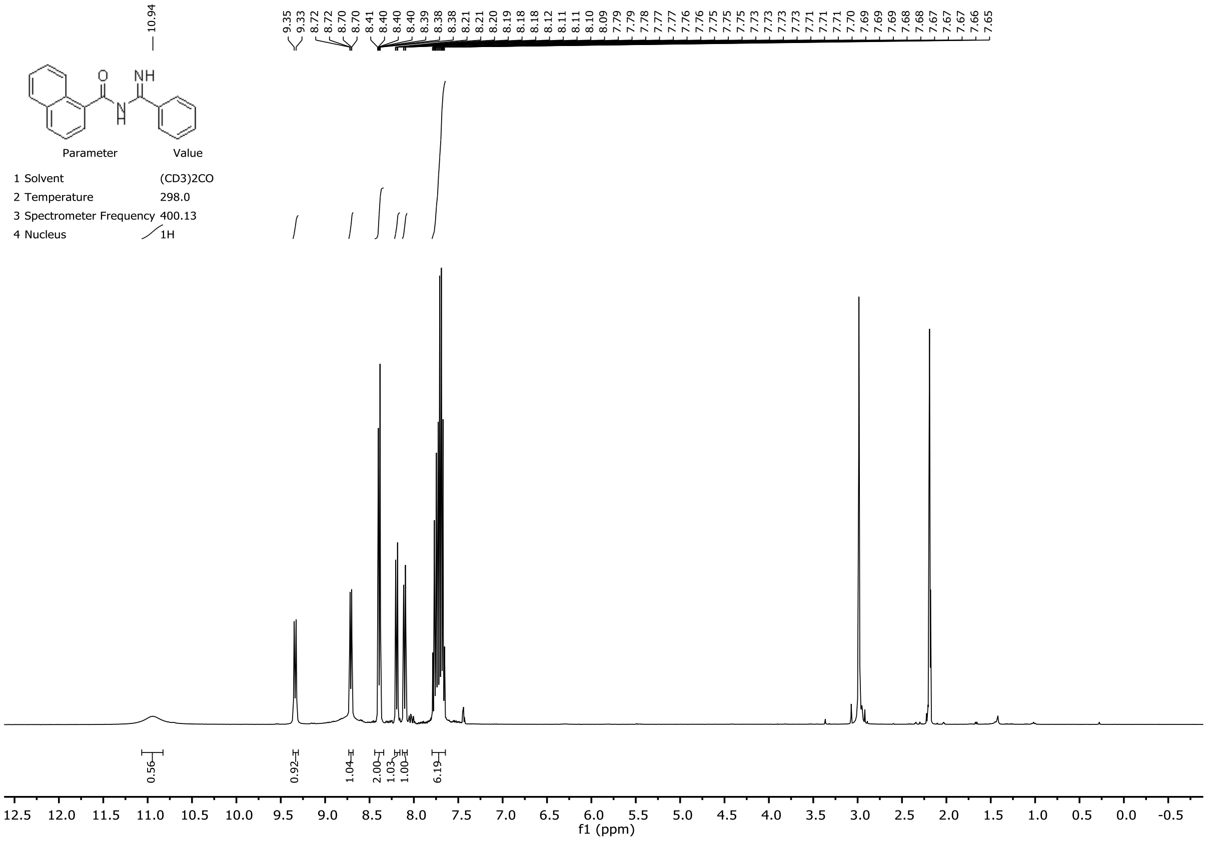

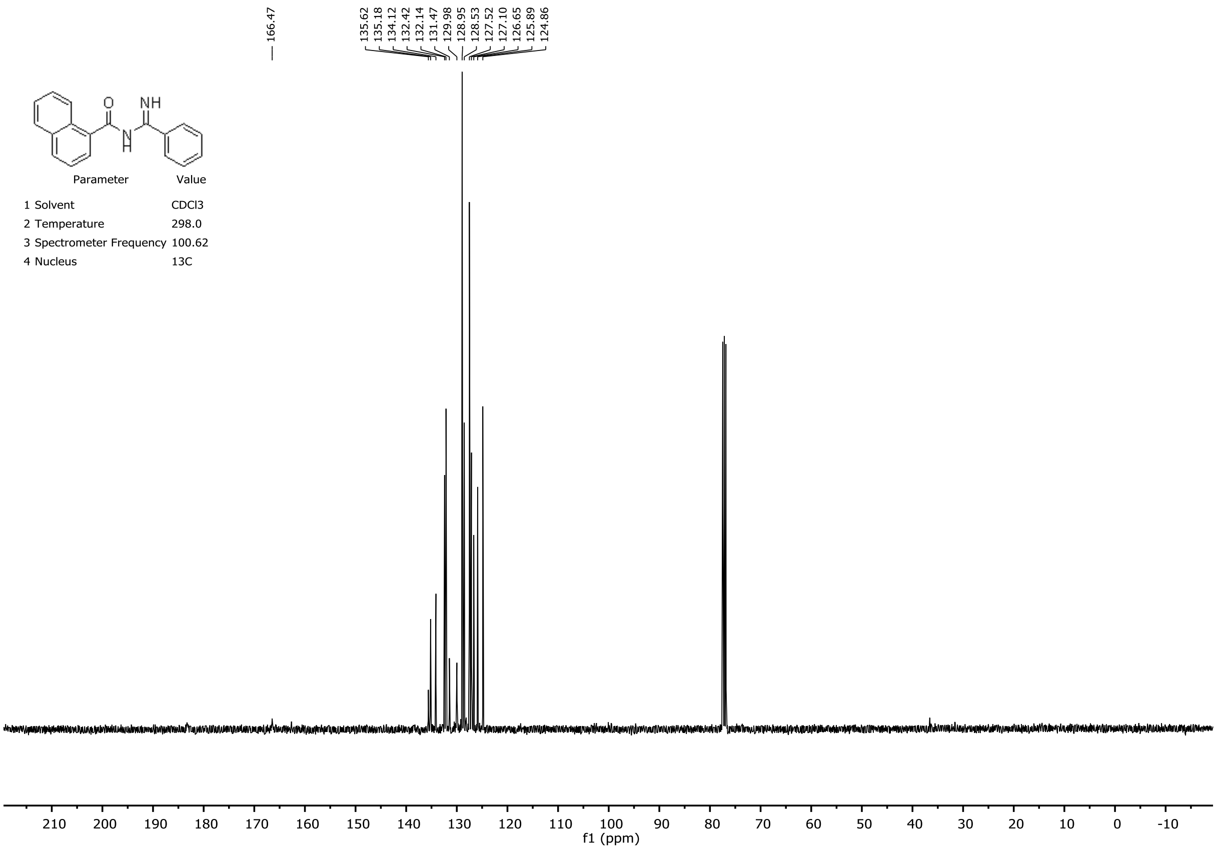

*N-(Imino(phenyl)methyl)-1-naphthamide **3k** CAS: 101716-52-3*

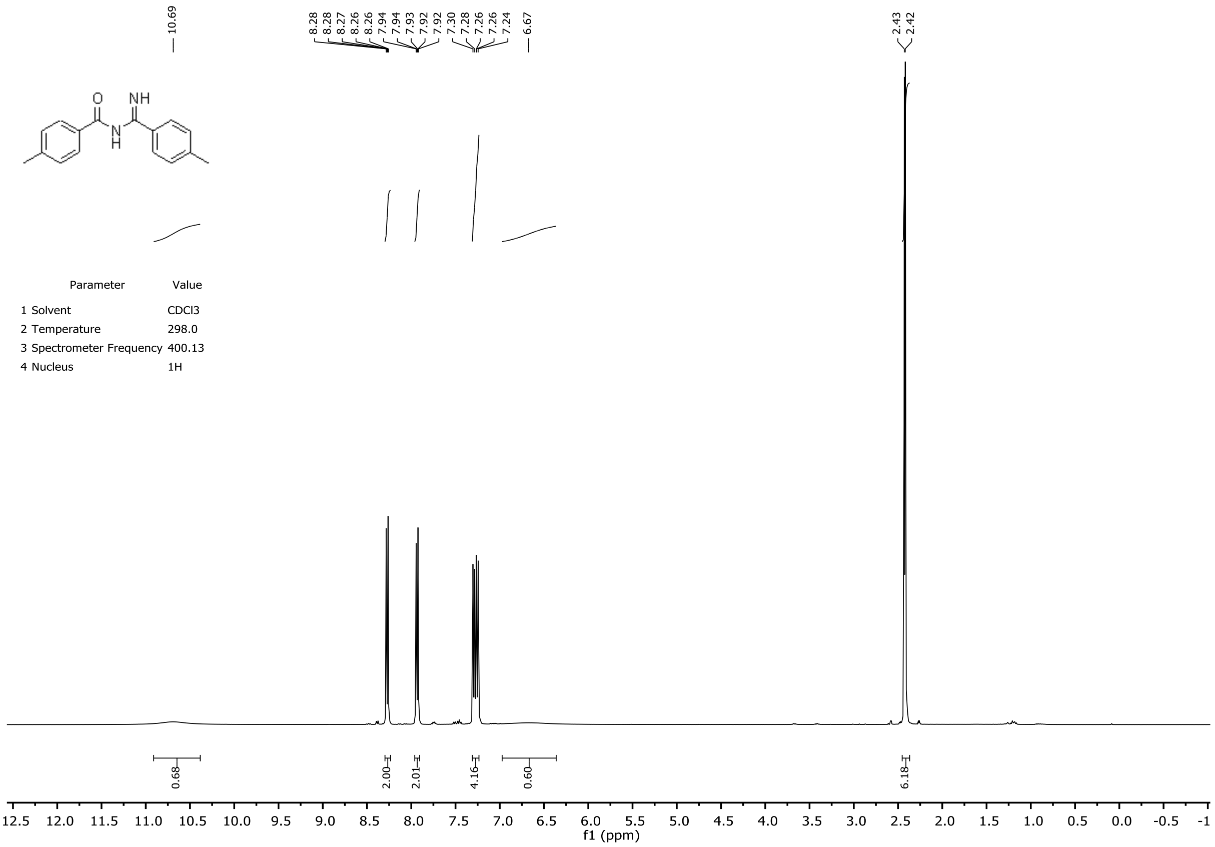

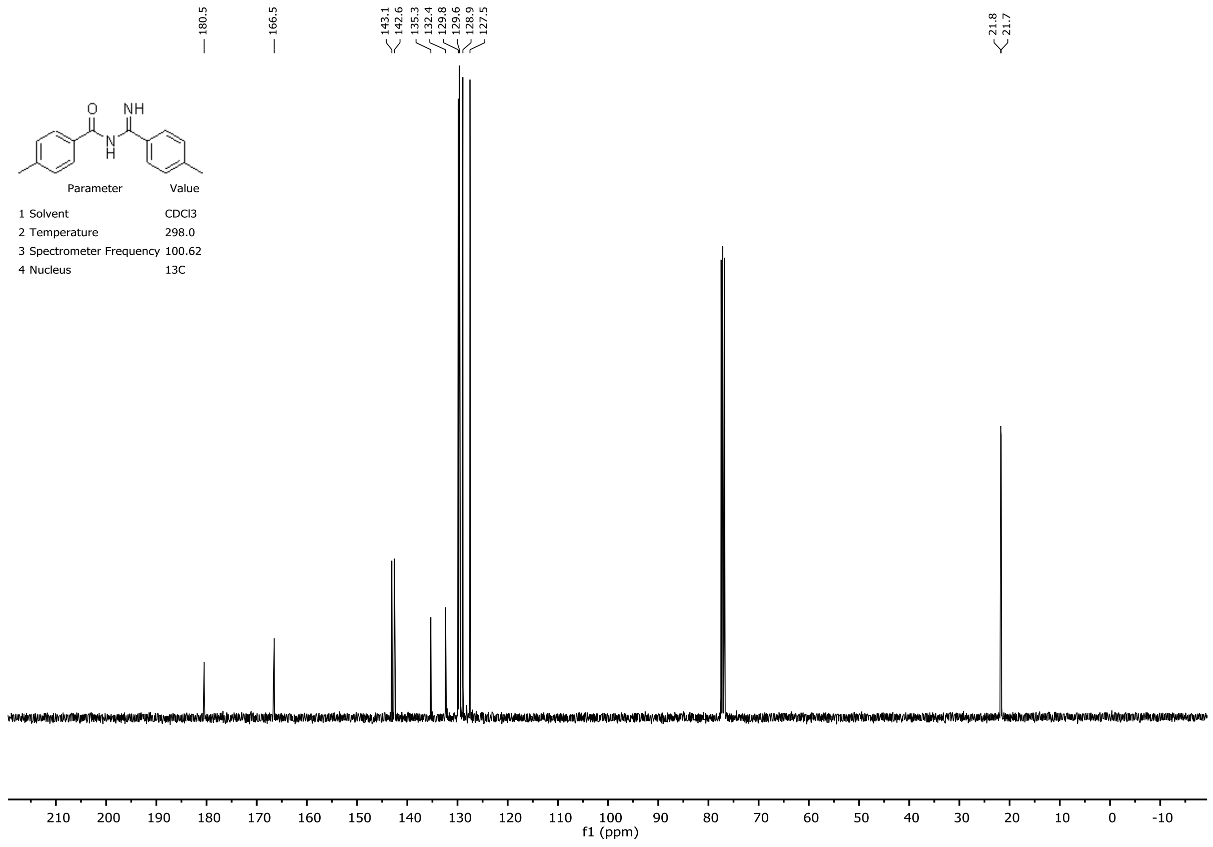

*N-(imino(p-tolyl)methyl)-4-methylbenzamide **3l** CAS: -*

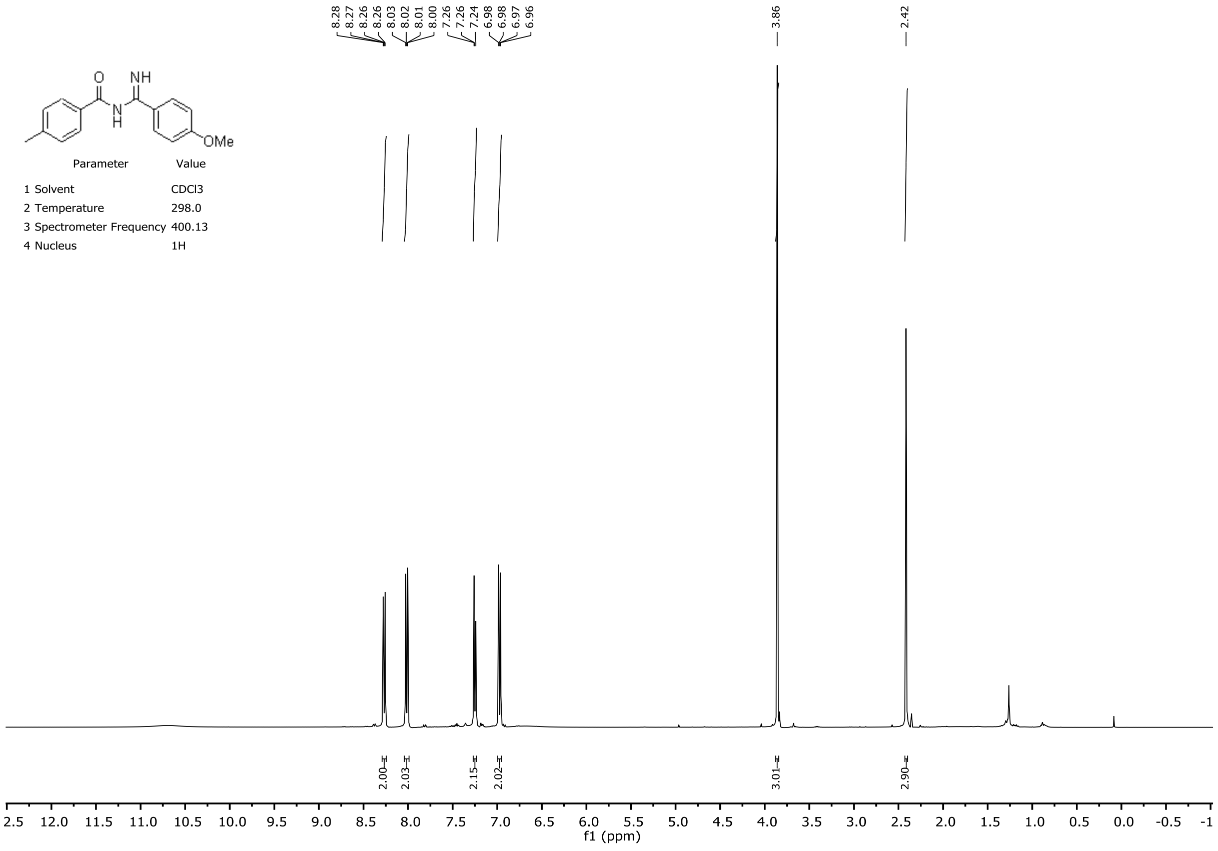

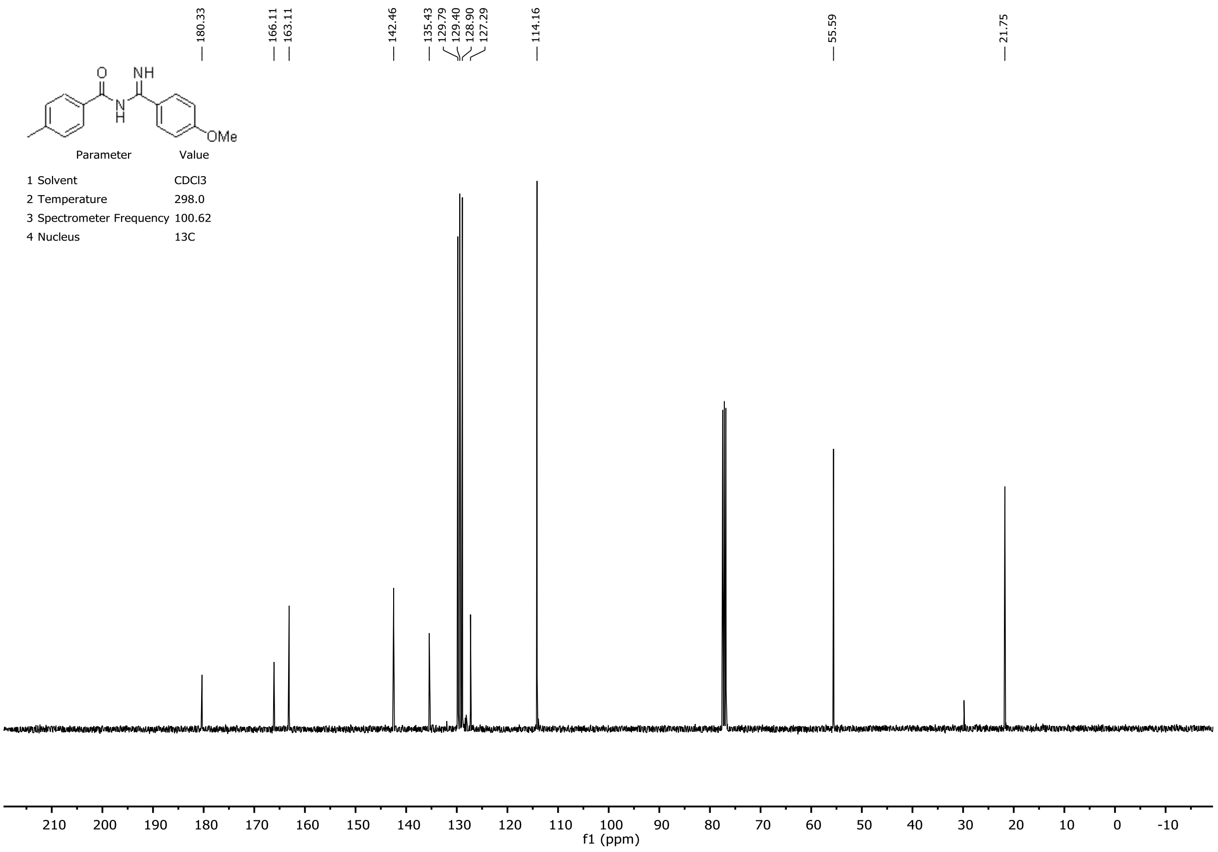

N-(imino(4-methoxyphenyl)methyl)-4-methylbenzamide ***3m*** *CAS: -*

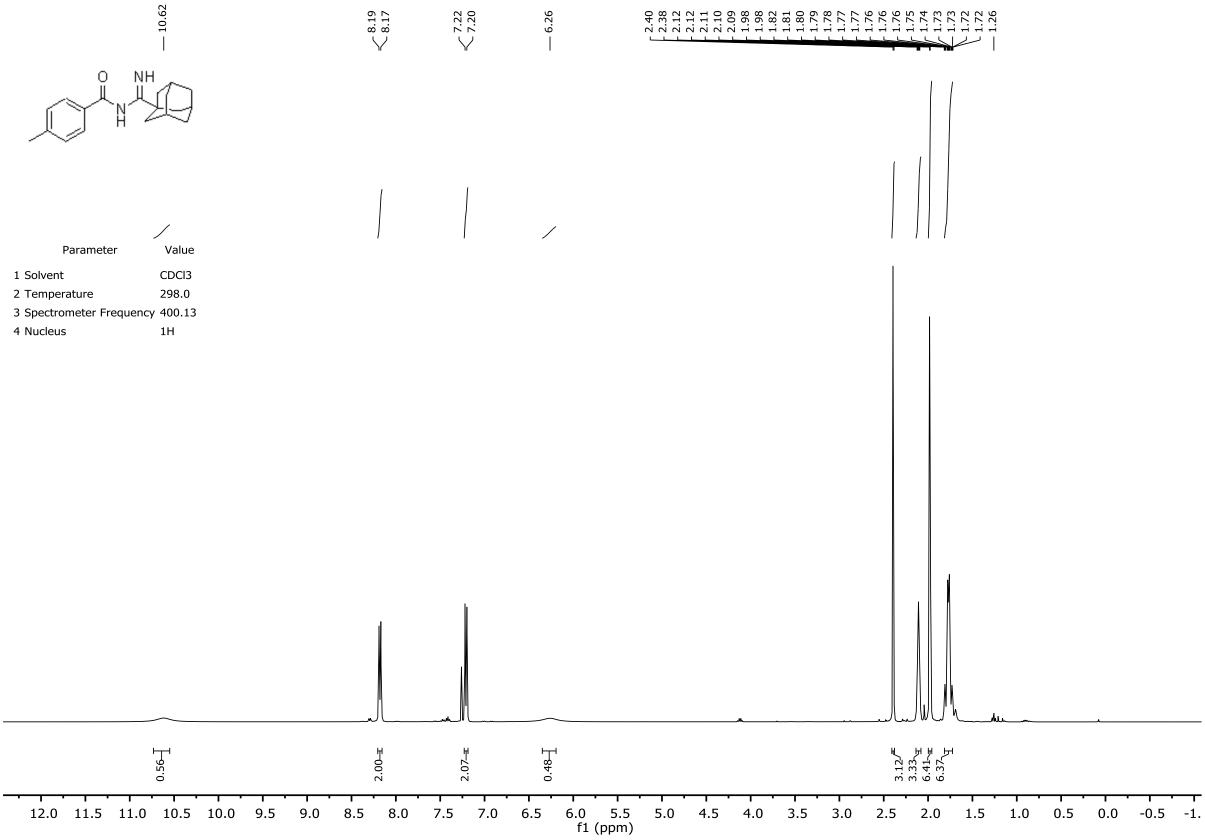

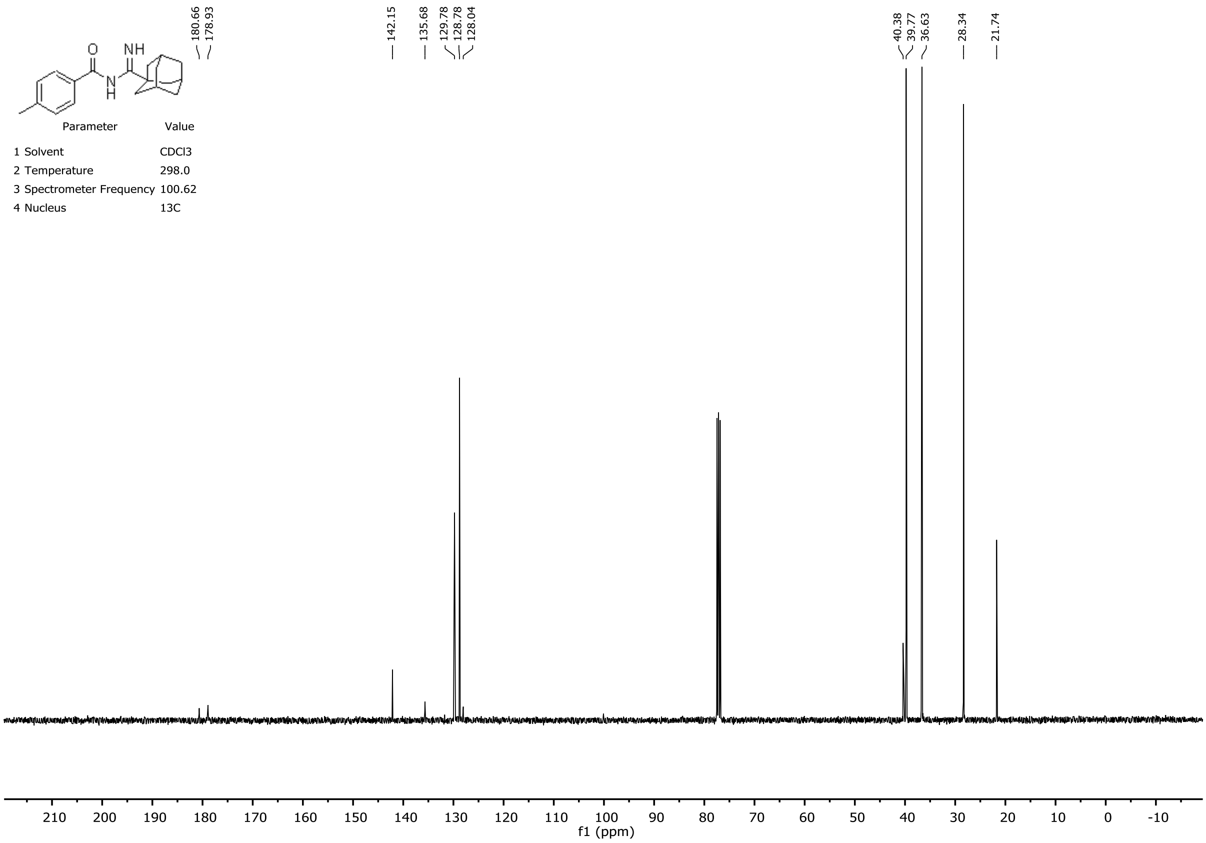

N-(adamantan-1-yl(imino)methyl)-4-methylbenzamide ***3n*** *CAS: -*

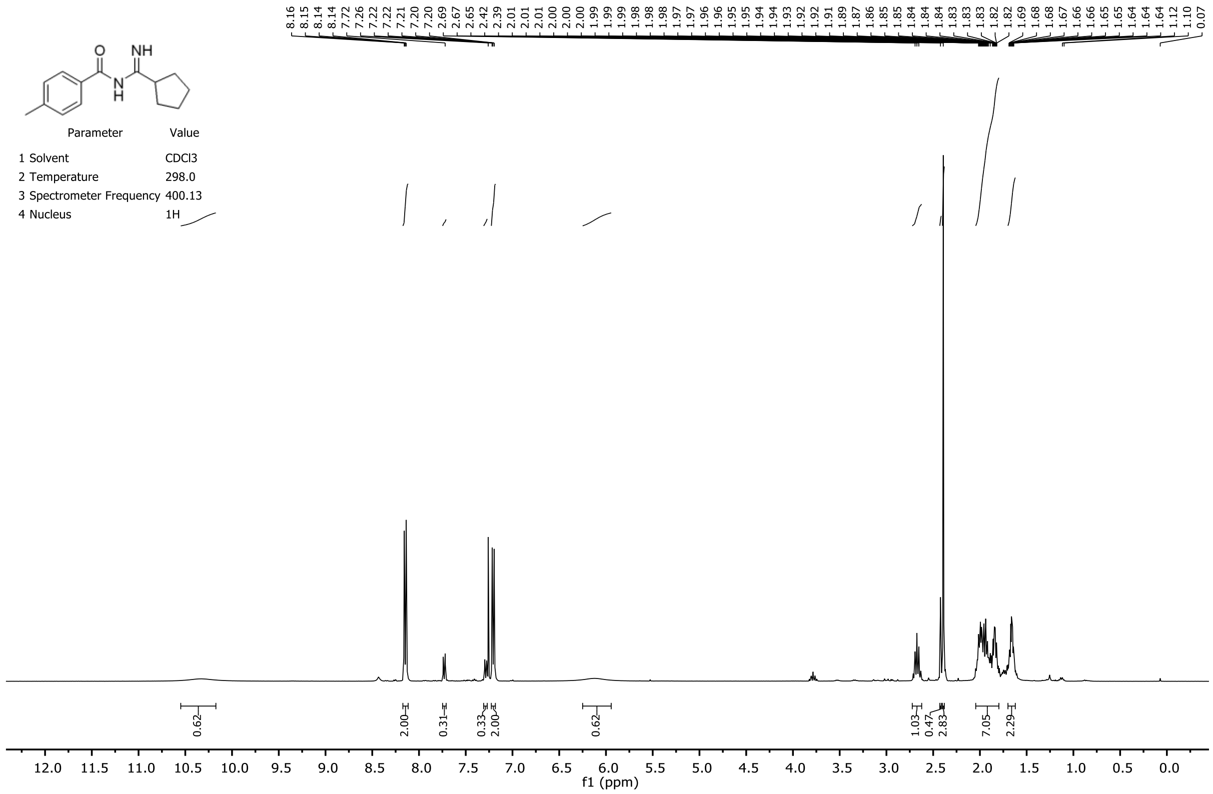

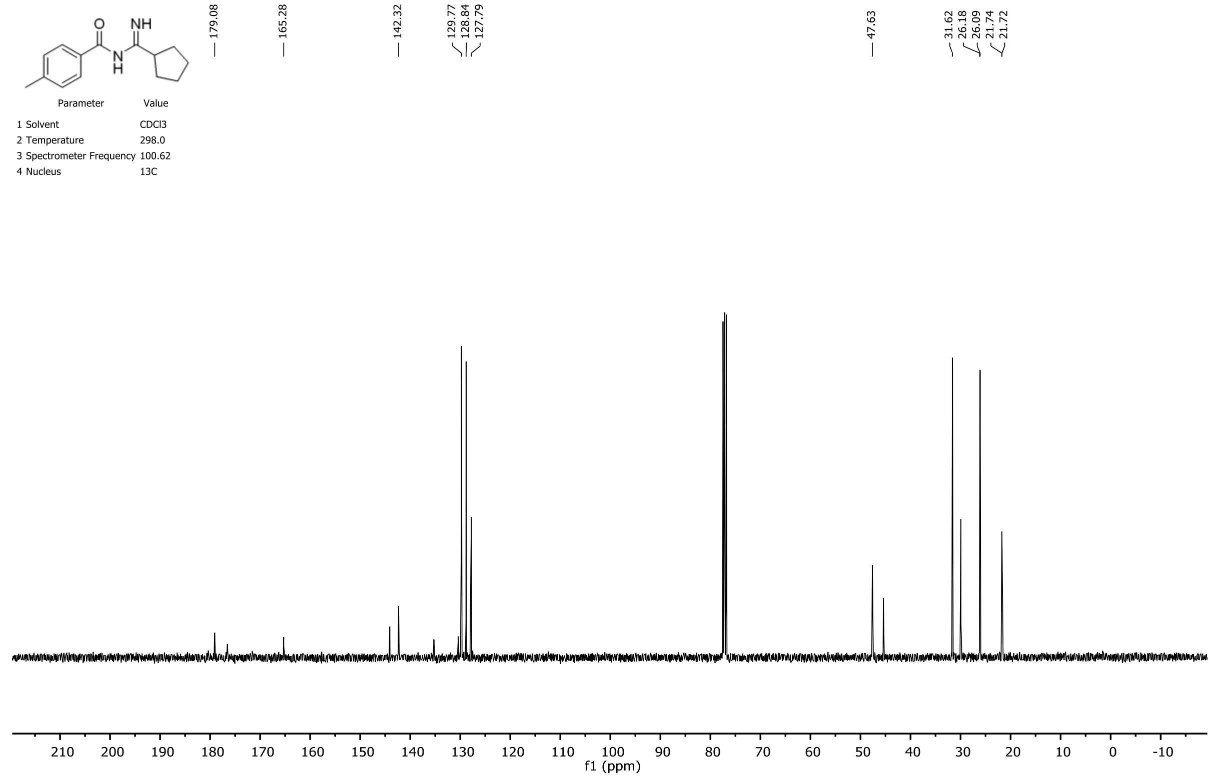

N-(cyclopentyl(imino)methyl)-4-methylbenzamide ***3o*** *CAS:-*

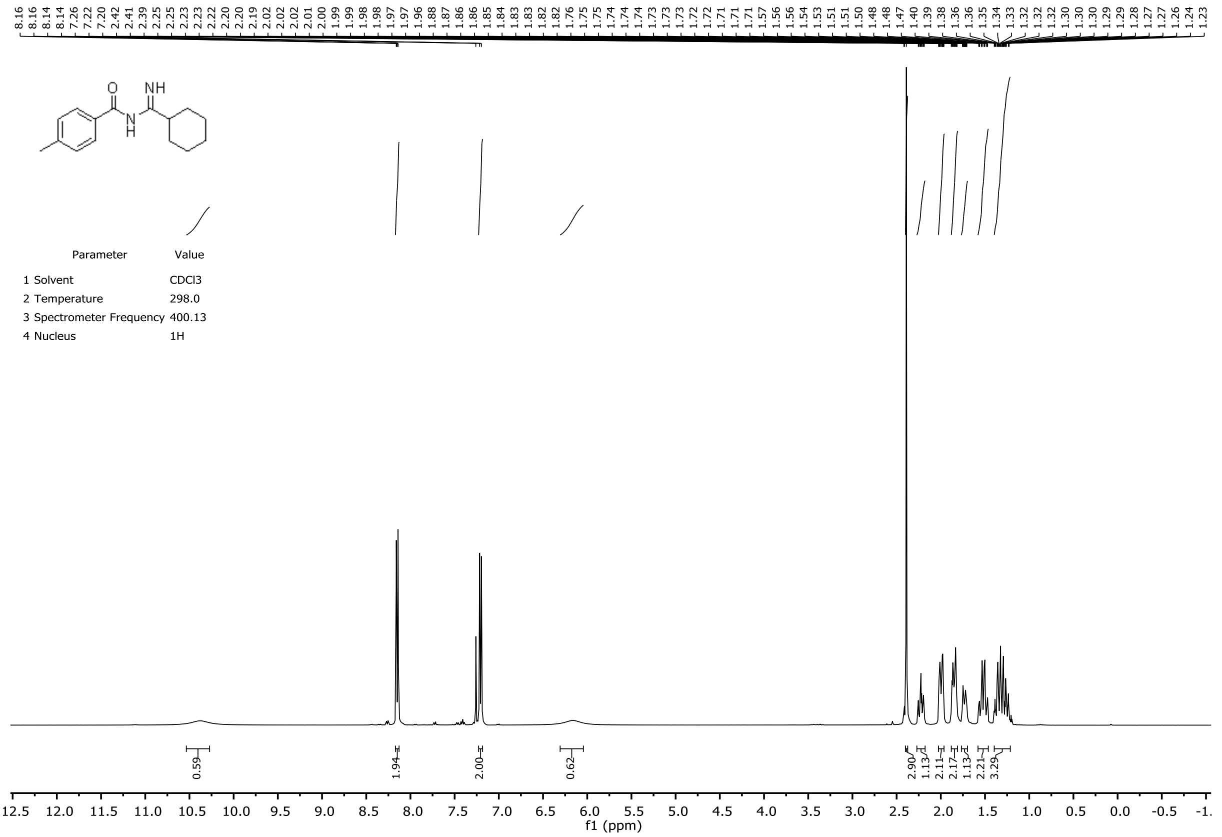

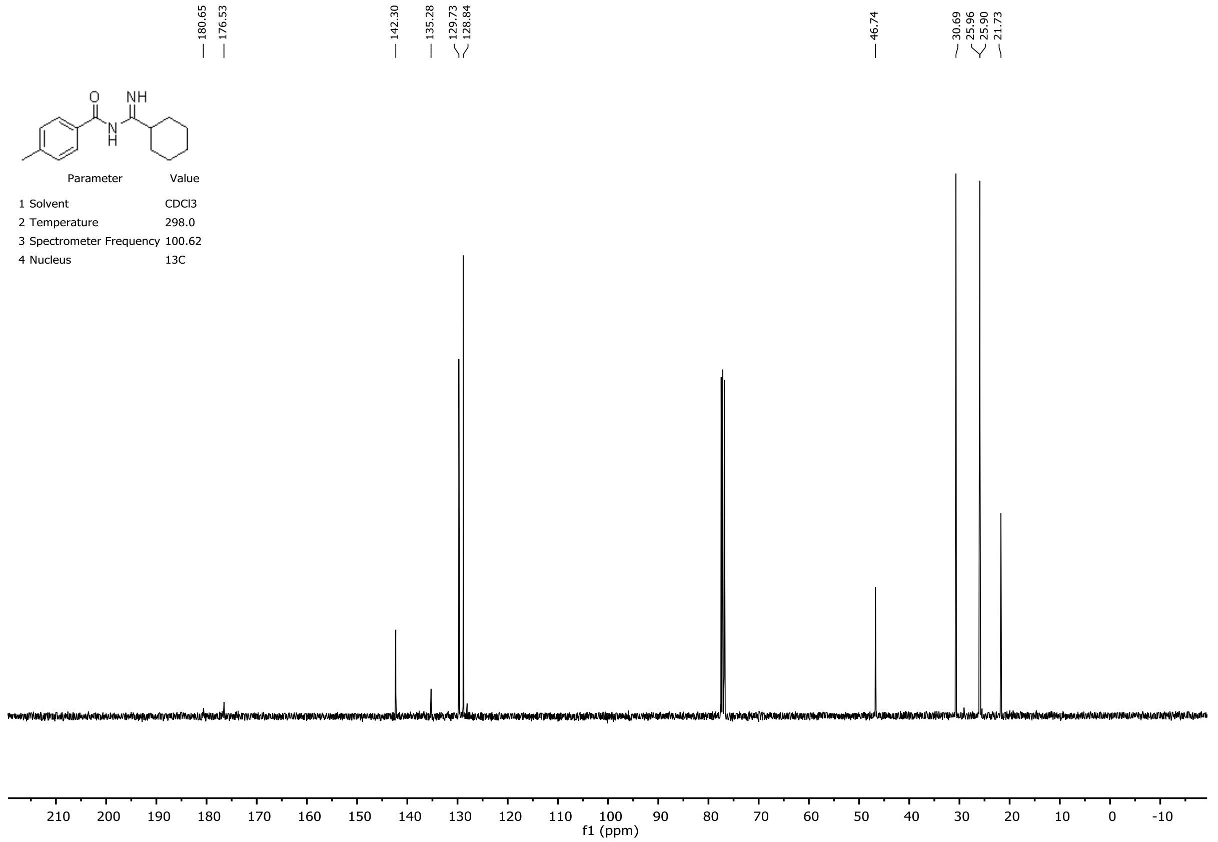

N-(cyclohexyl(imino)methyl)-4-methylbenzamide ***3p*** *CAS: -*

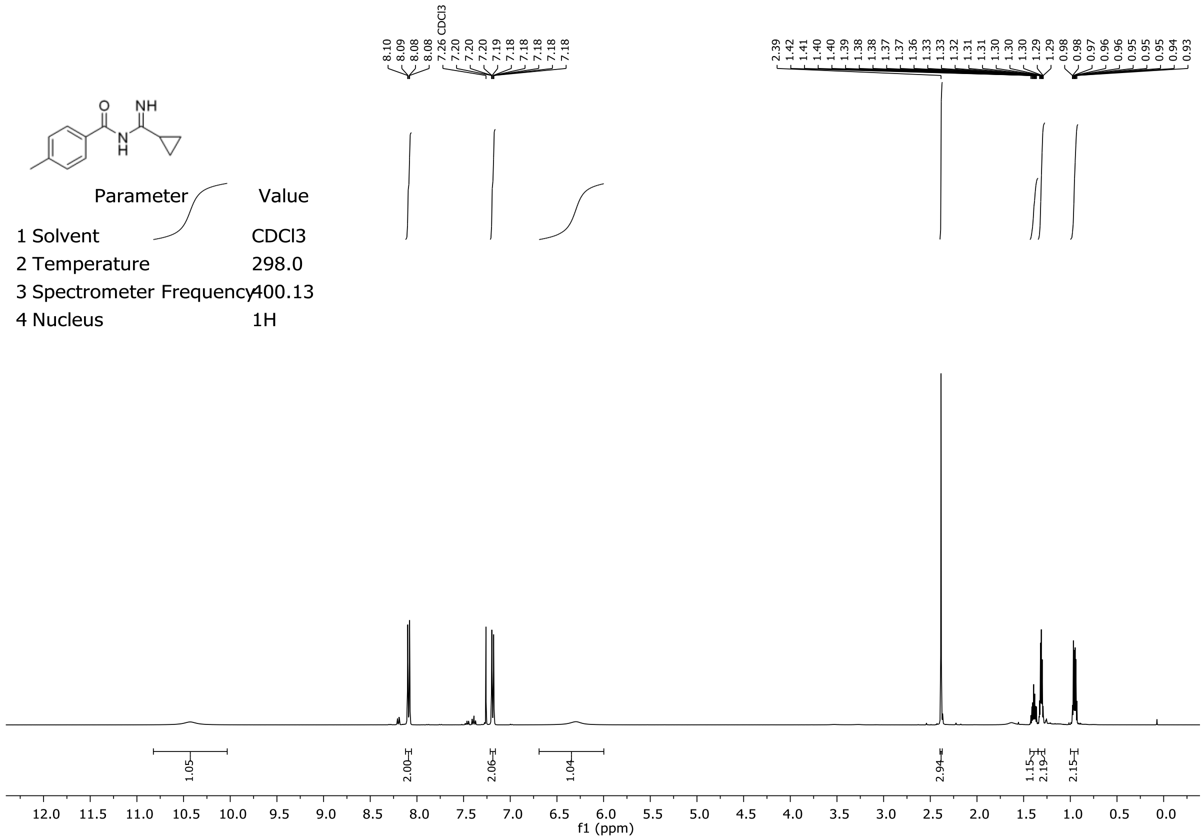

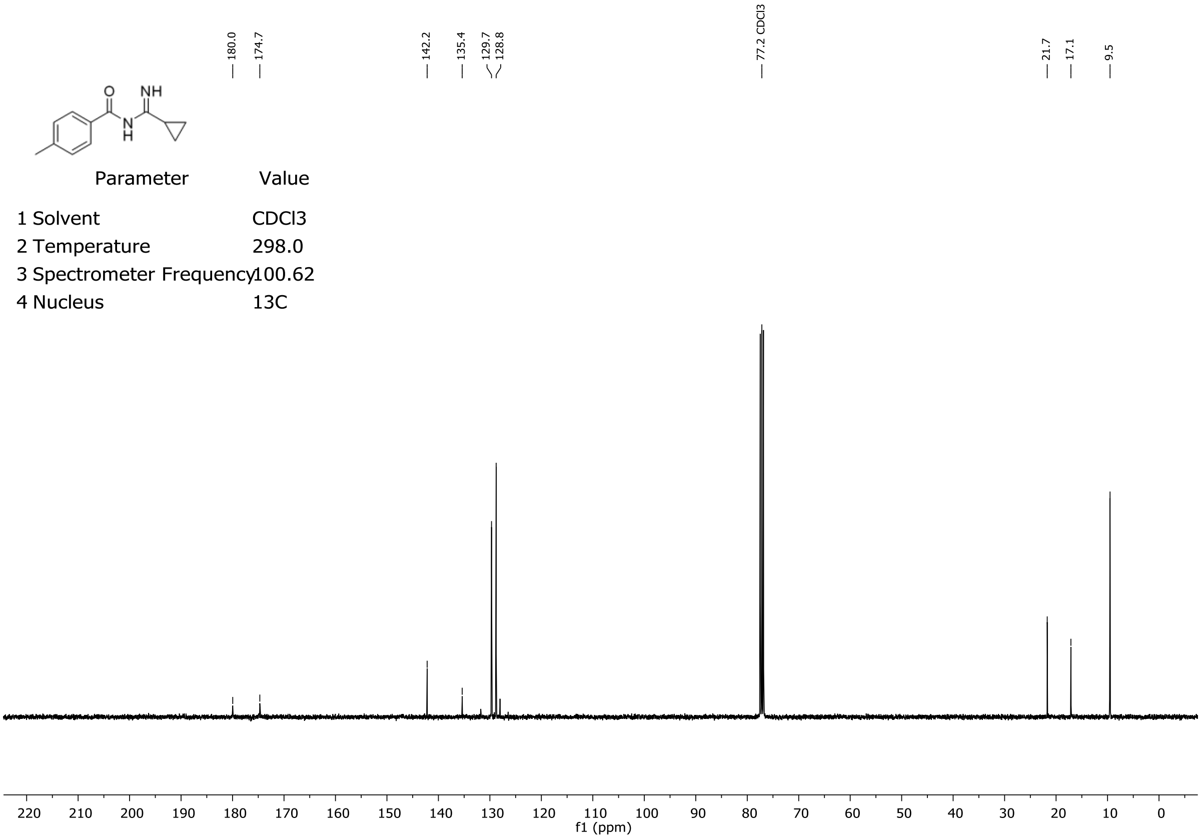

*N-(cyclopropyl(imino)methyl)-4-methylbenzamide **3q** CAS:-*

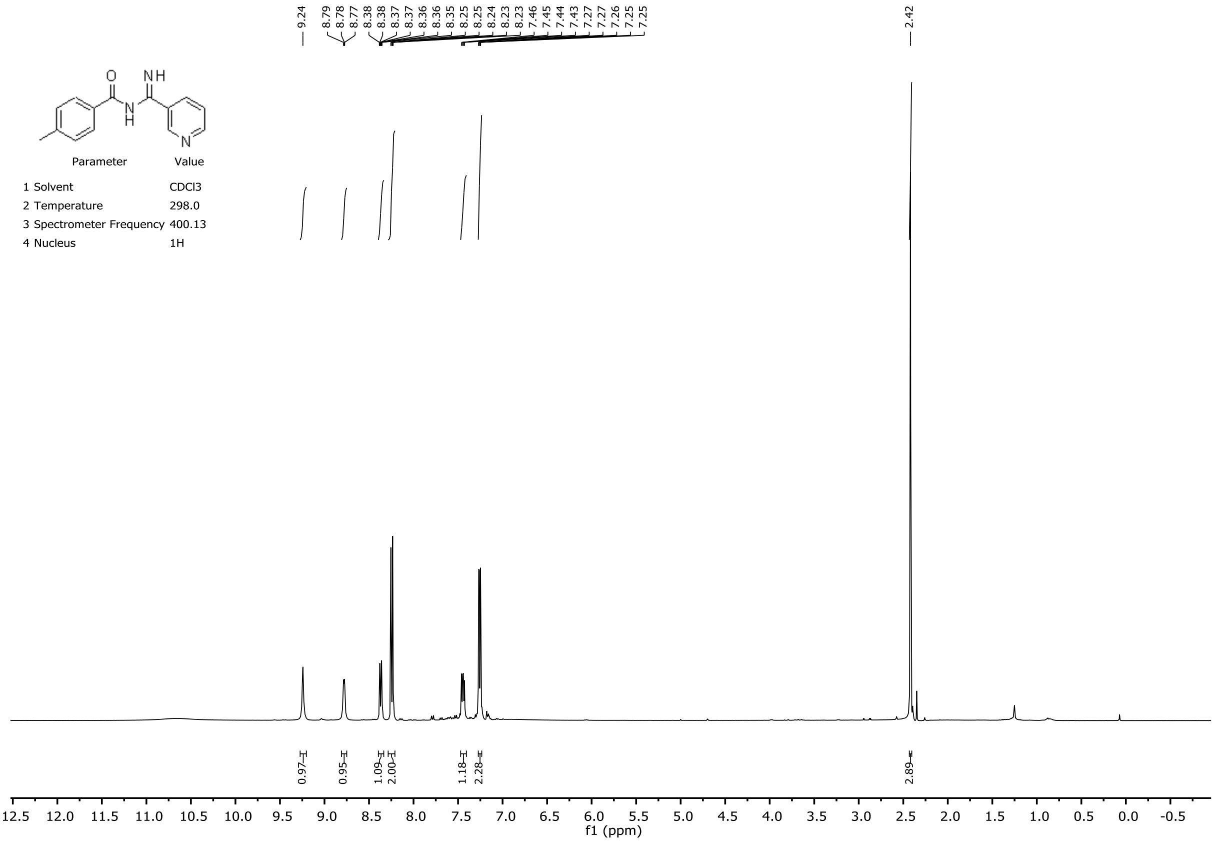

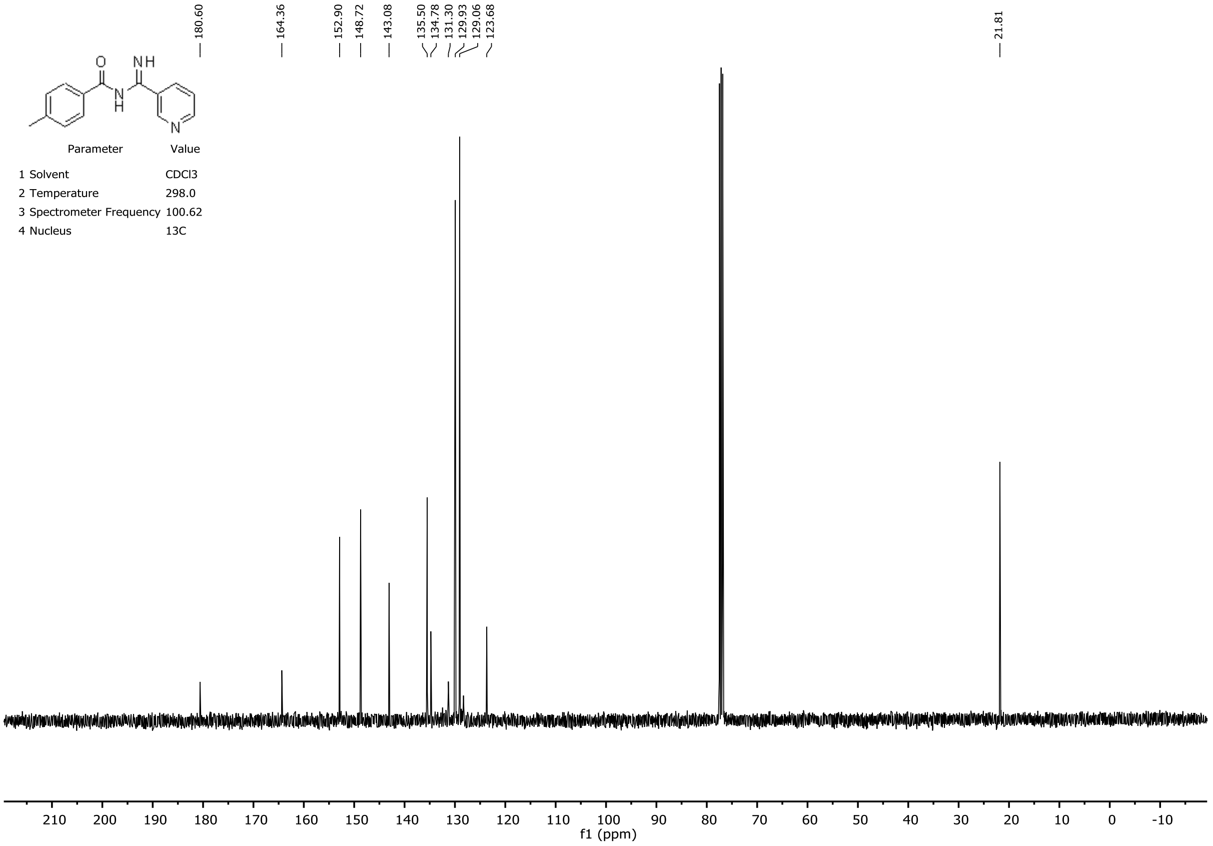

*N-(imino(pyridin-3-yl)methyl)-4-methylbenzamide **3r** CAS: -*

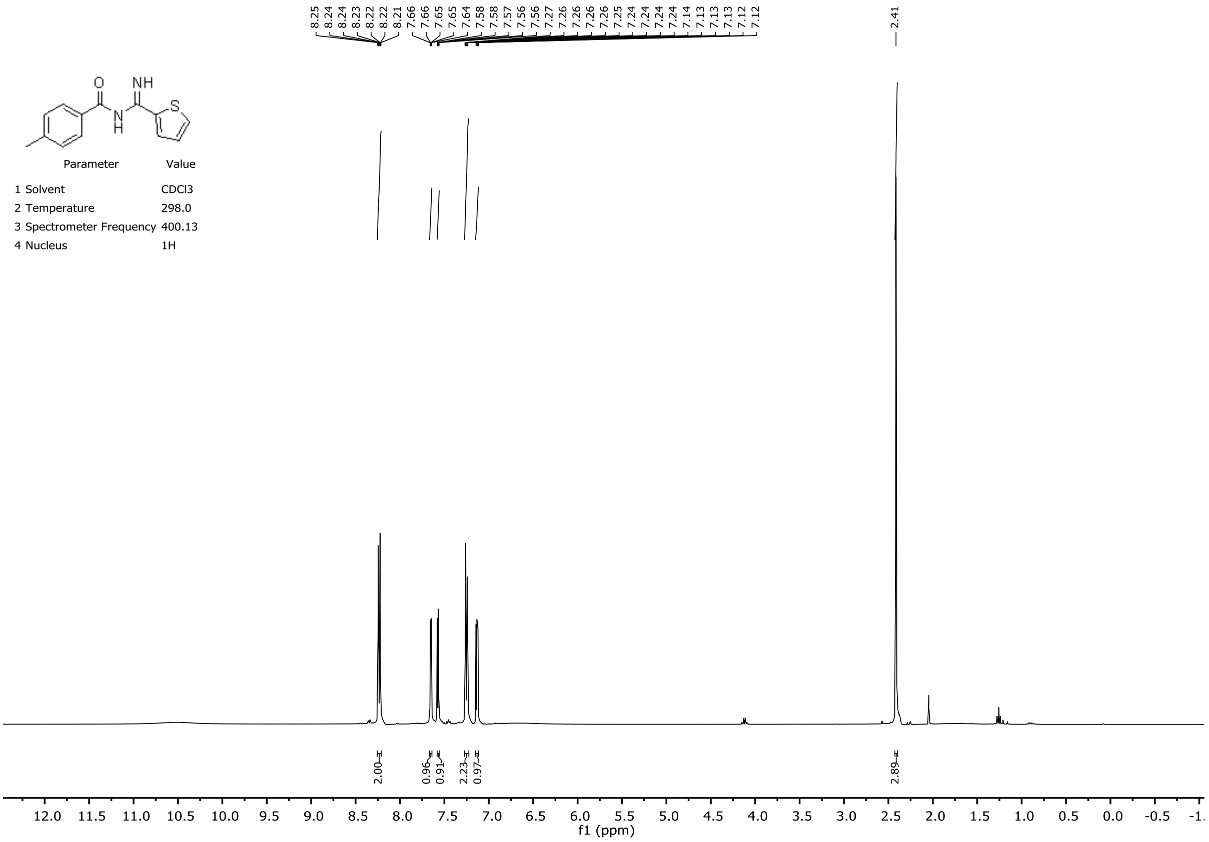

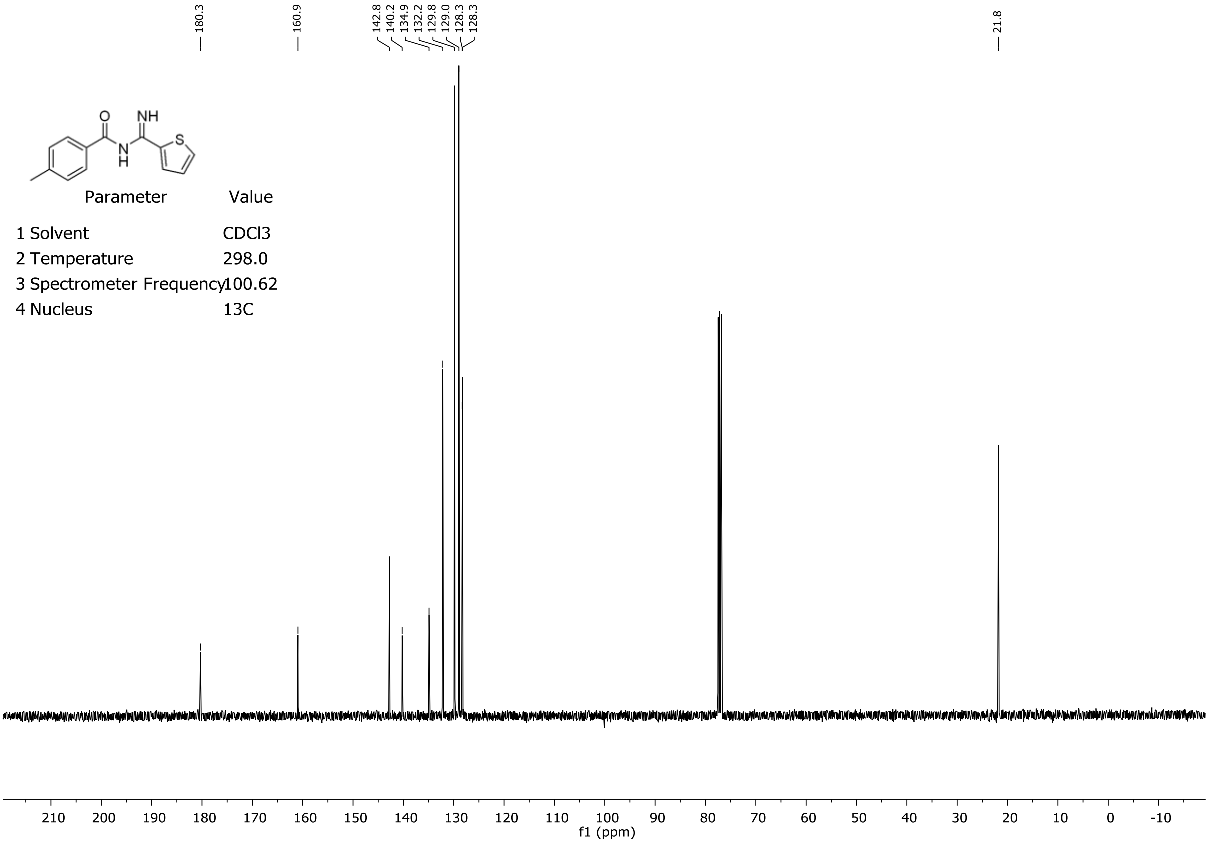

*N-(imino(thiophen-2-yl)methyl)-4-methylbenzamide **3s** CAS: 883041-84-7*

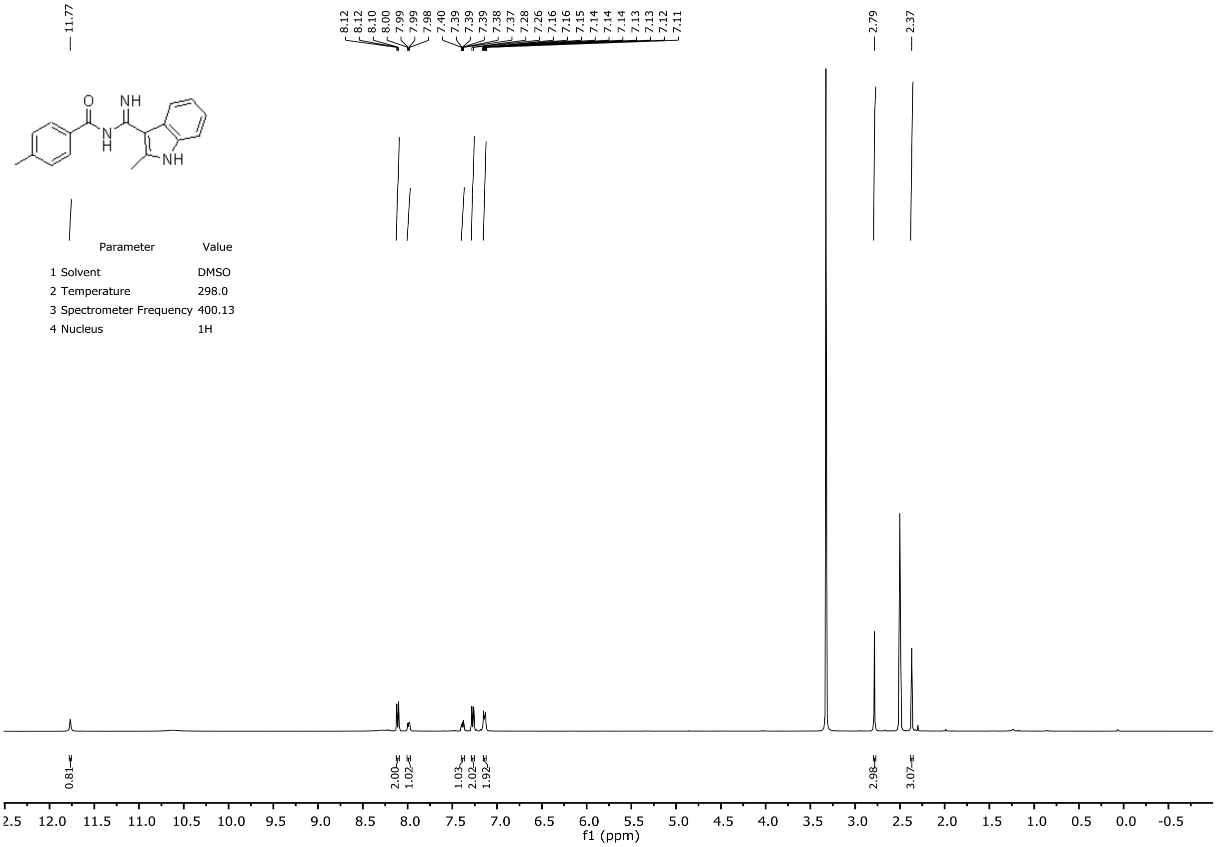

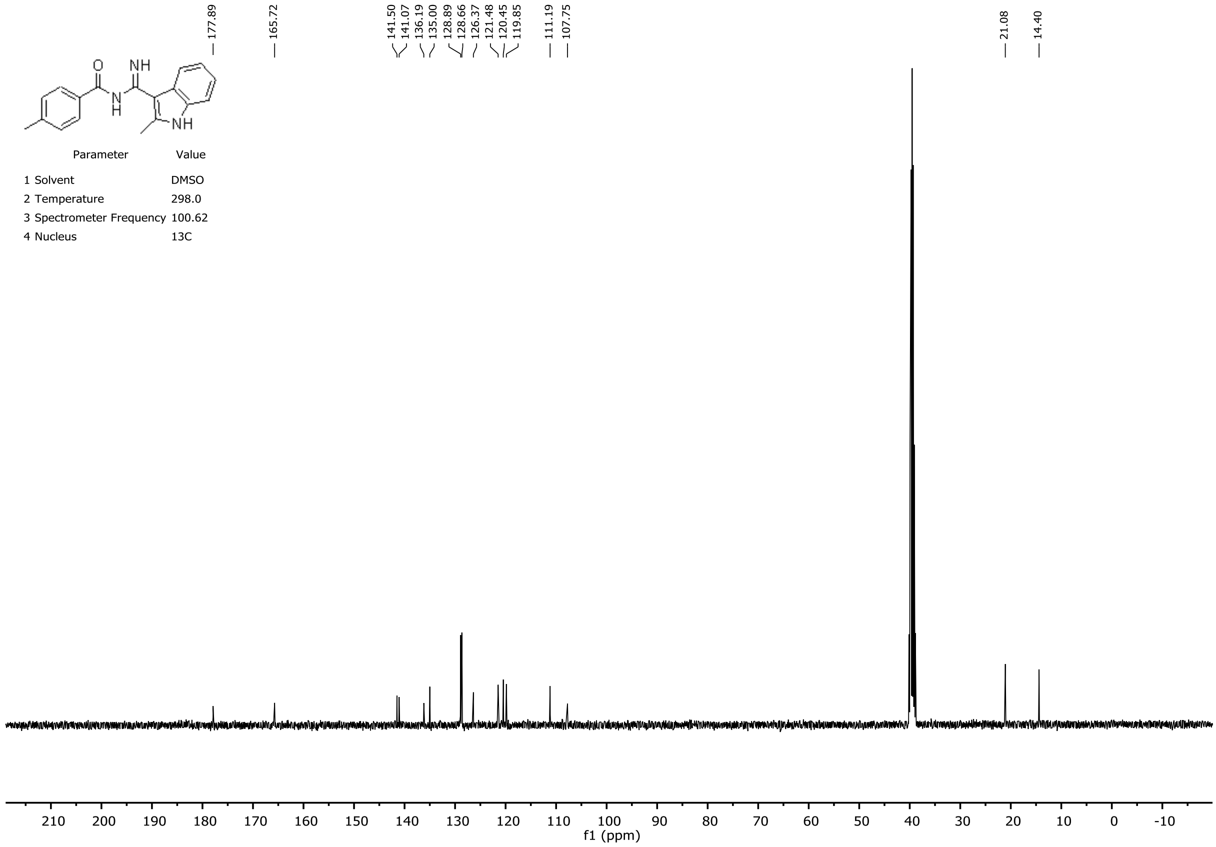

*N-(imino(2-methyl-1H-indol-3-yl)methyl)-4-methylbenzamide **3t** CAS: -*

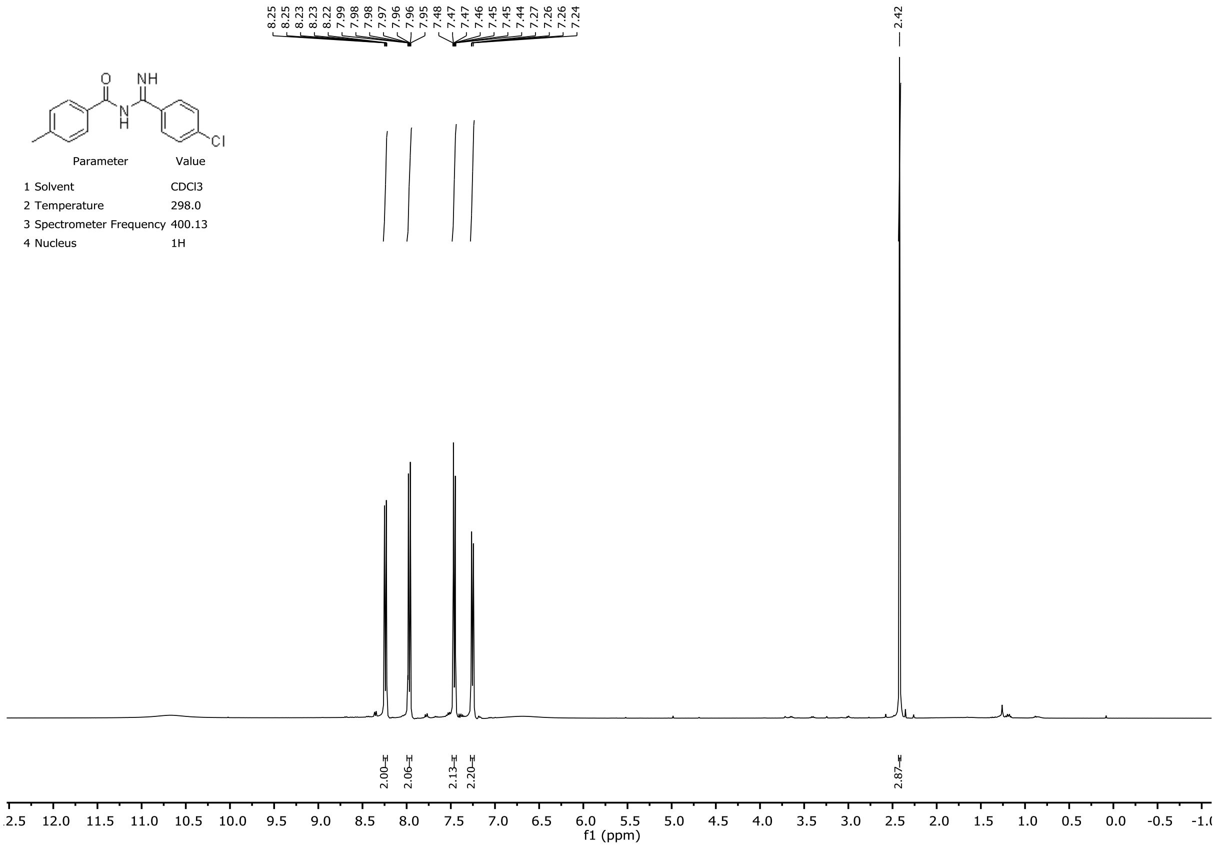

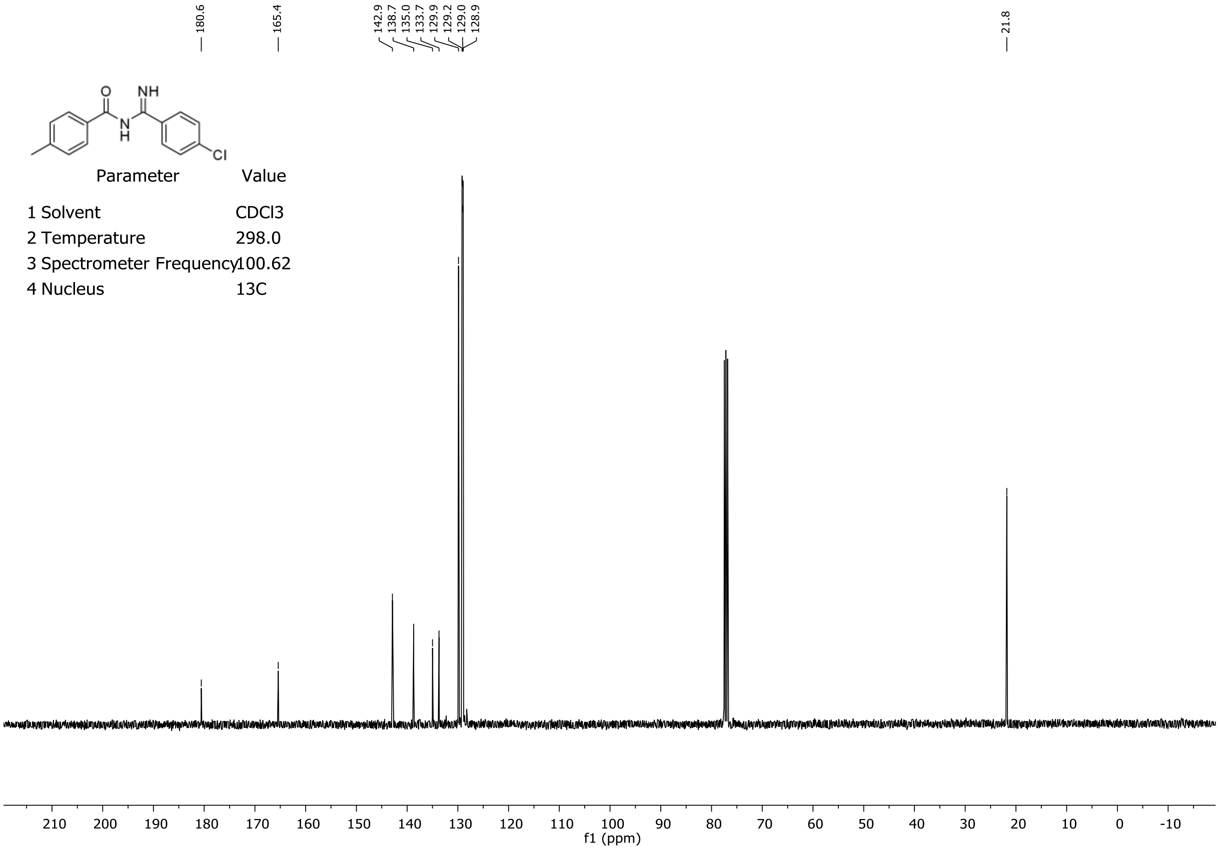

*N-((4-chlorophenyl)(imino)methyl)-4-methylbenzamide **3u** CAS: -*

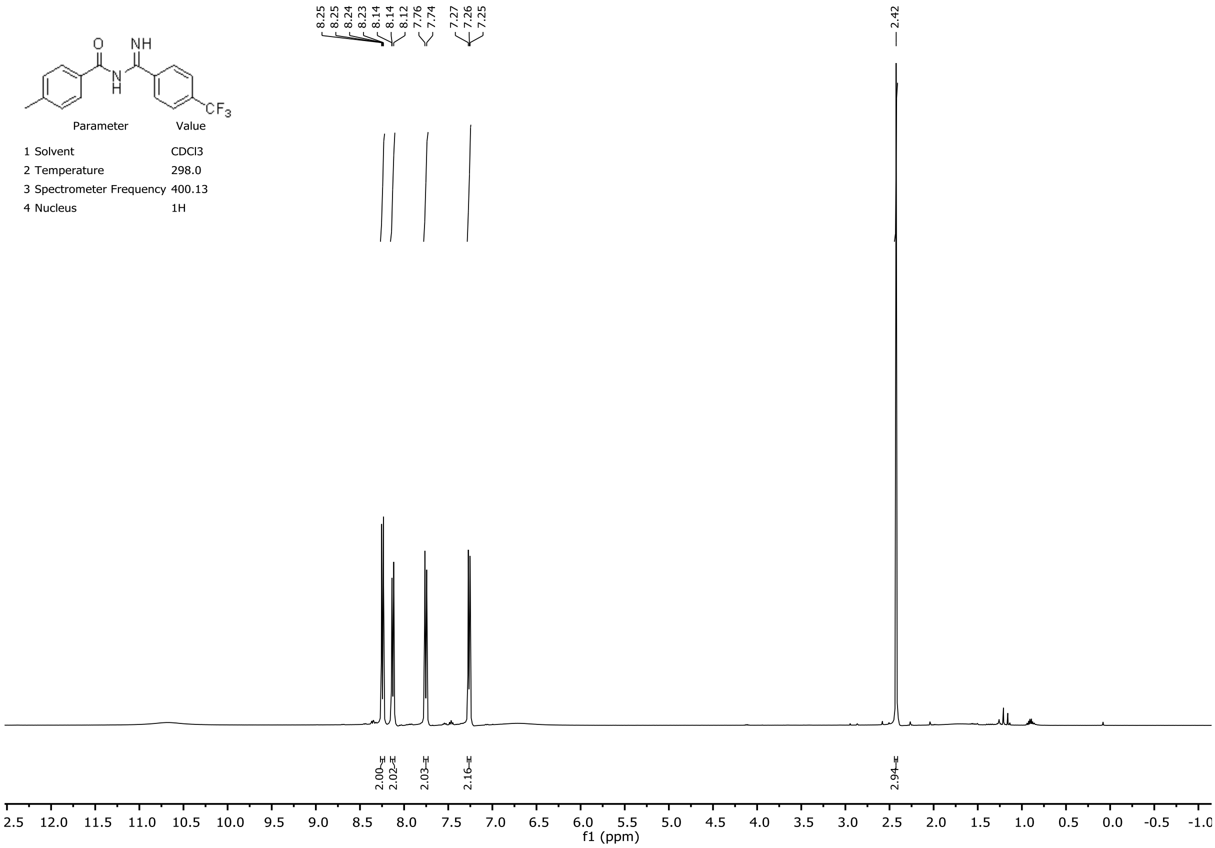

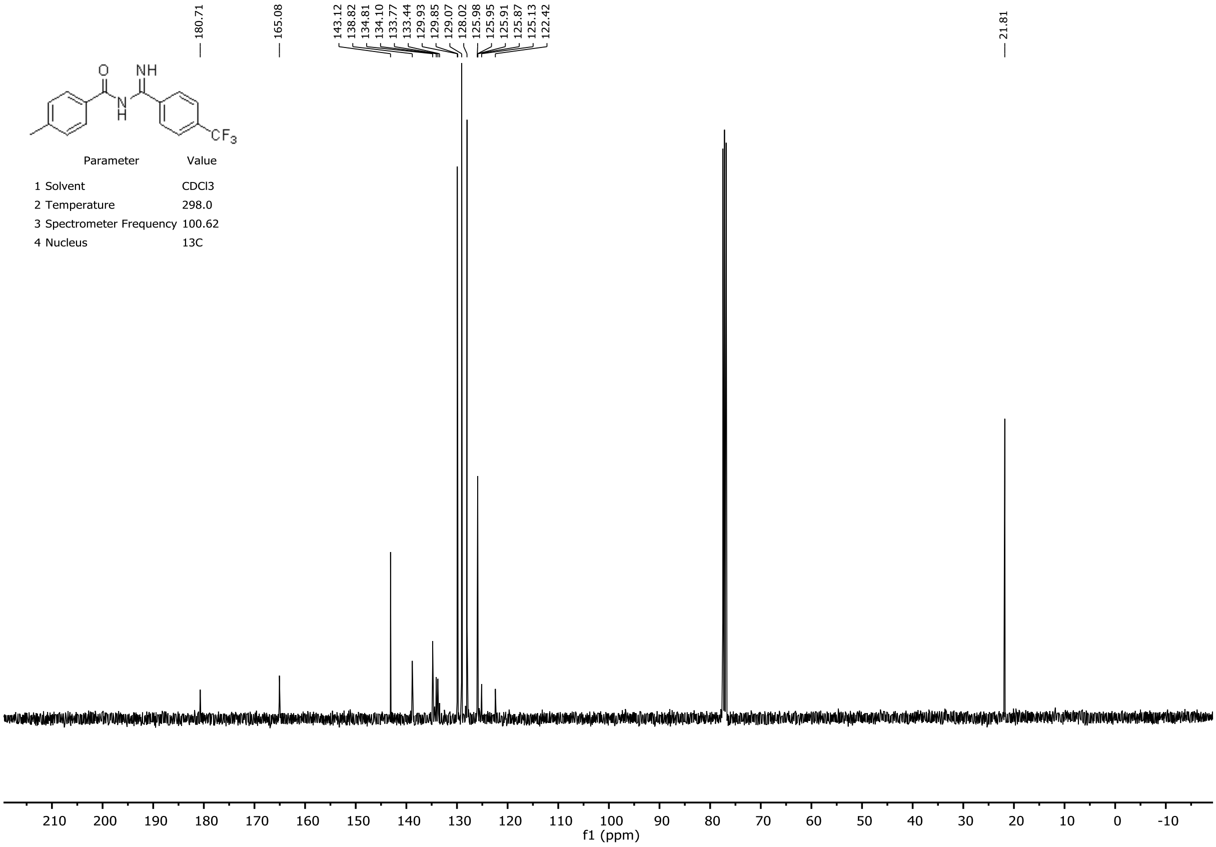

N-(imino(4-(trifluoromethyl)phenyl)methyl)-4-methylbenzamide ***3v*** *CAS: -*

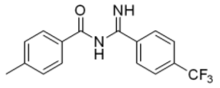

Parameter	Value
1 Solvent	CDCl3
2 Temperature	298.0
3 Spectrometer Frequency	376.47
4 Nucleus	19F

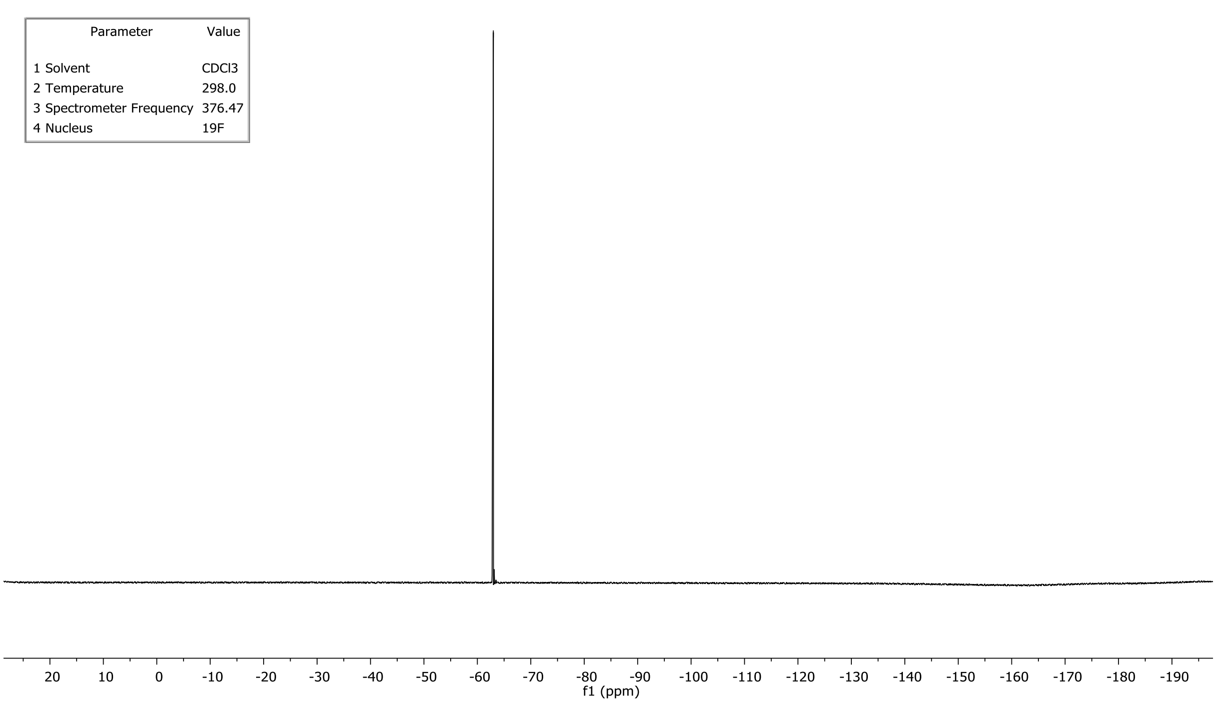

N-(imino(4-(trifluoromethyl)phenyl)methyl)-4-methylbenzamide ***3v*** *CAS: -*

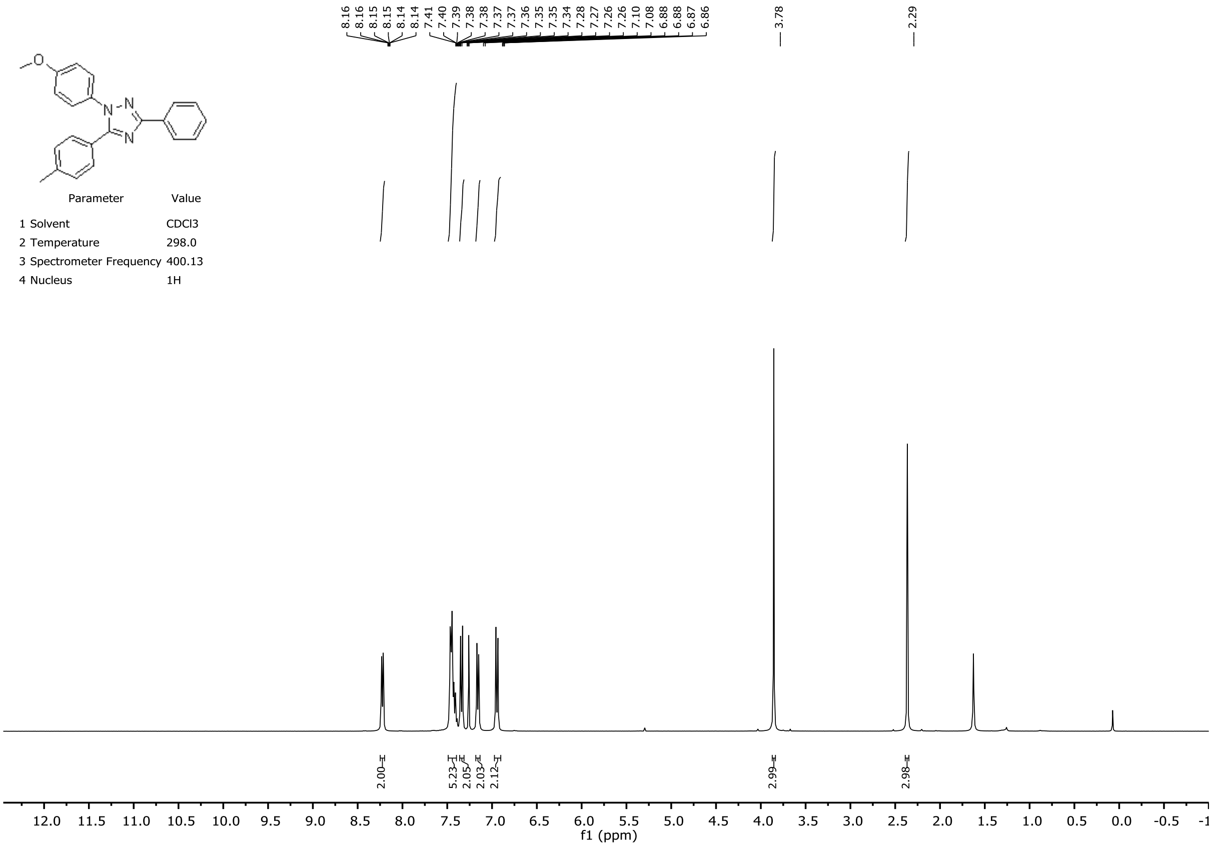

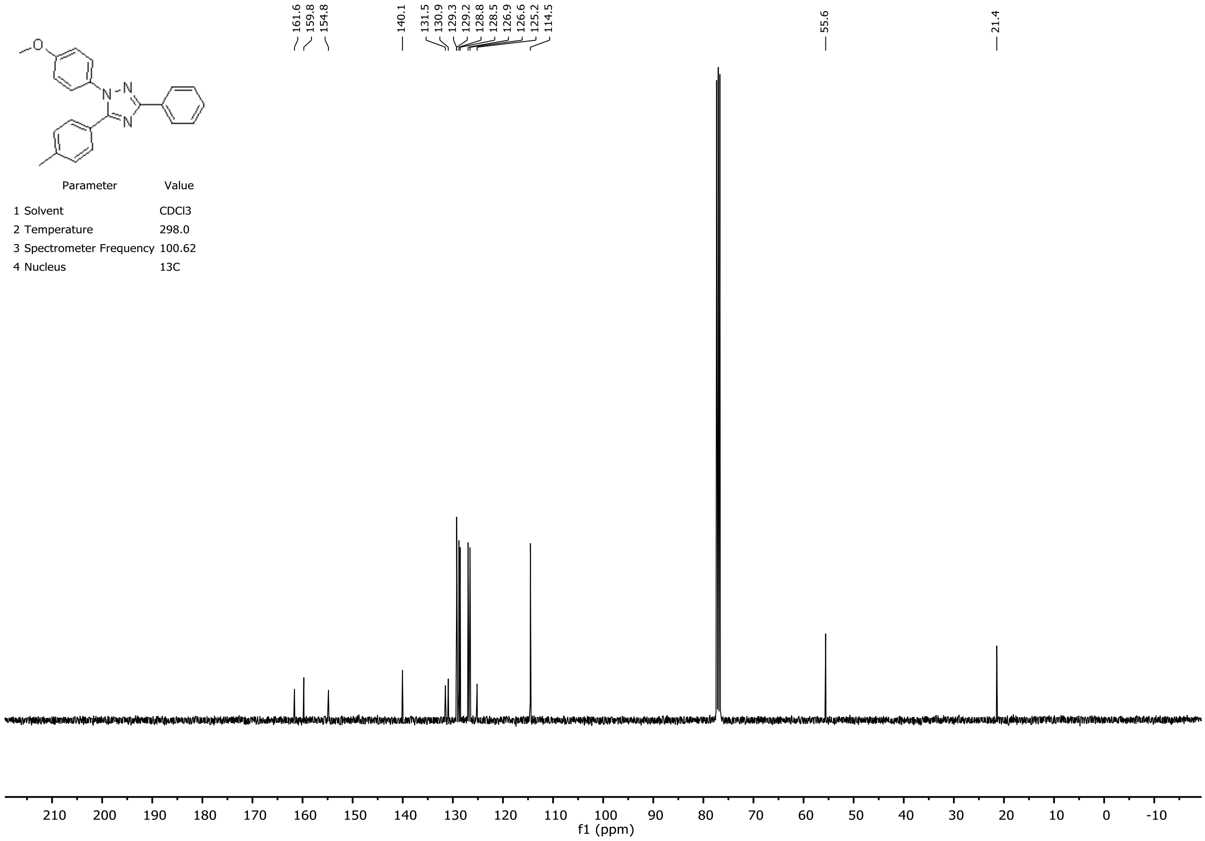

1-(4-methoxyphenyl)-3-phenyl-5-(p-tolyl)-1H-1,2,4-triazole **5** *CAS: -*

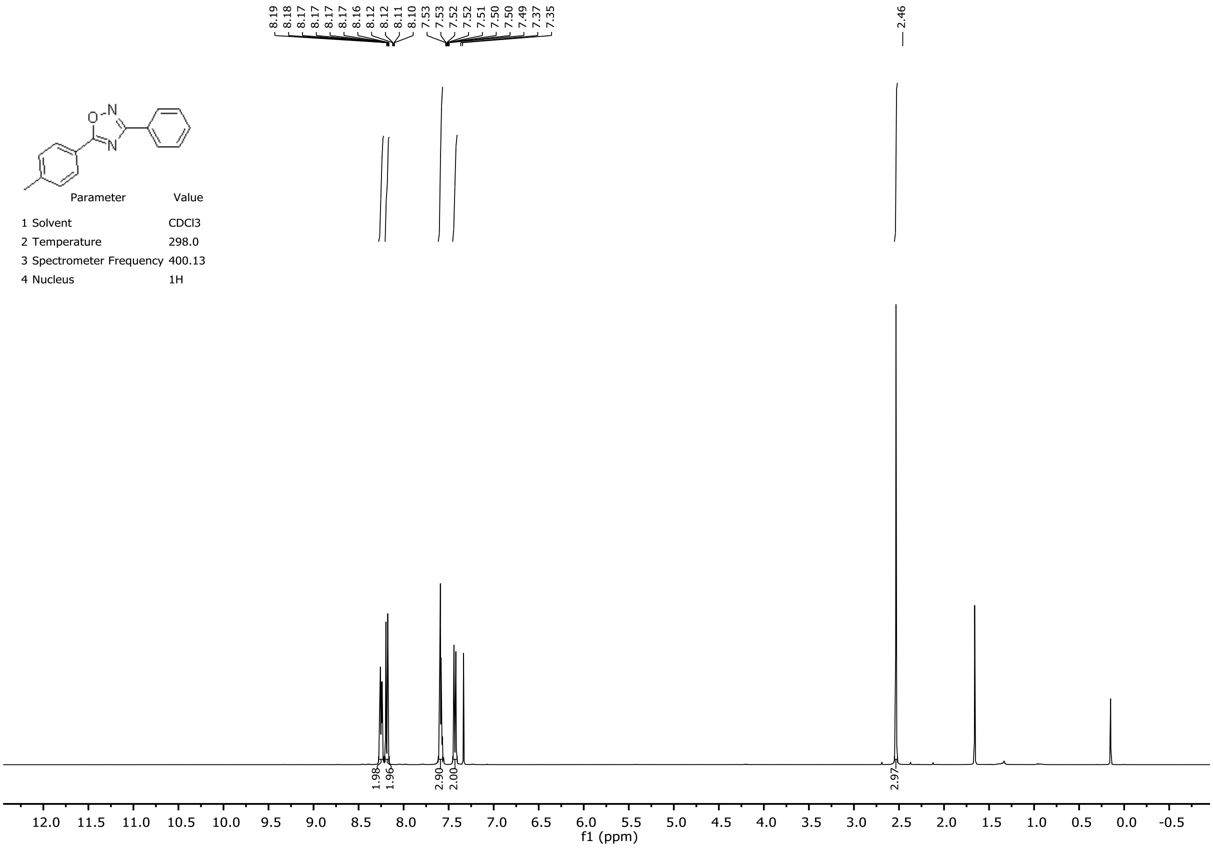

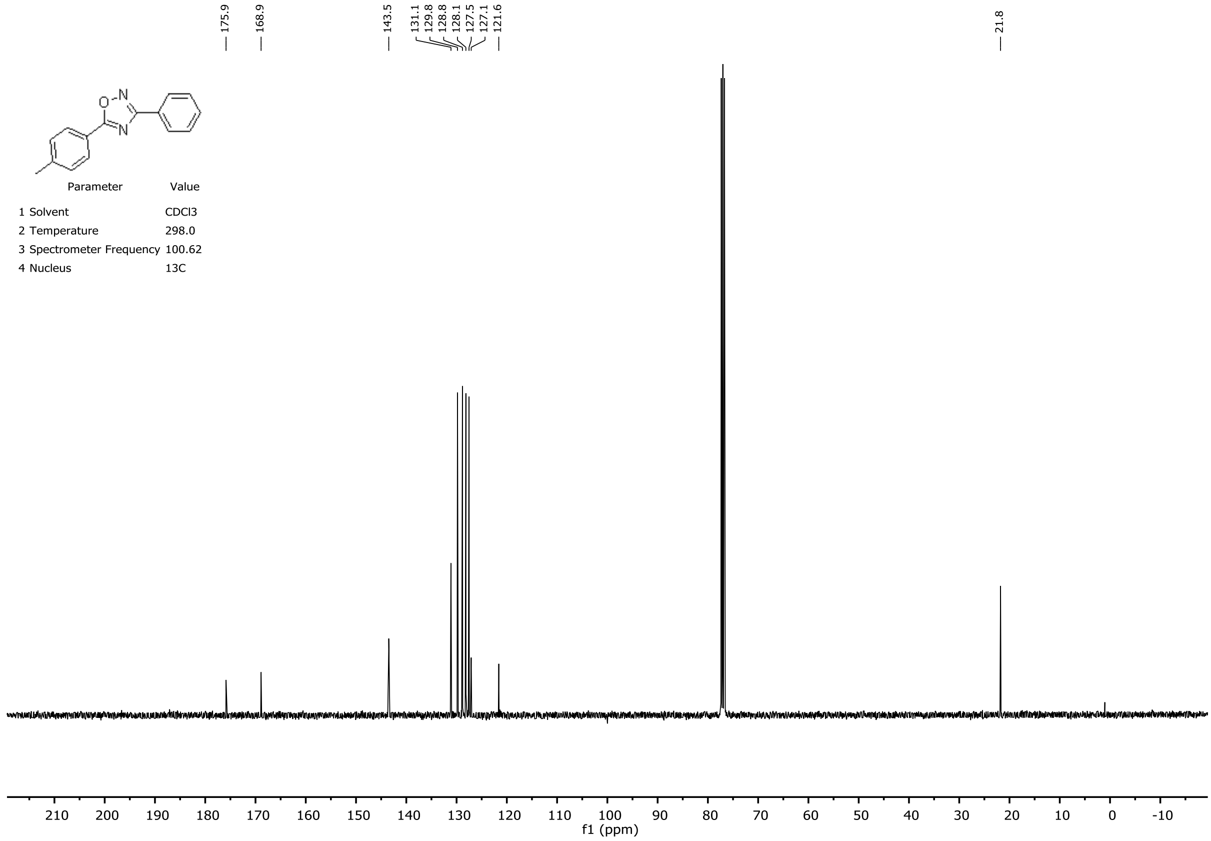

*3-phenyl-5-(p-tolyl)-1,2,4-oxadiazole **6a** CAS: 16112-24-6*

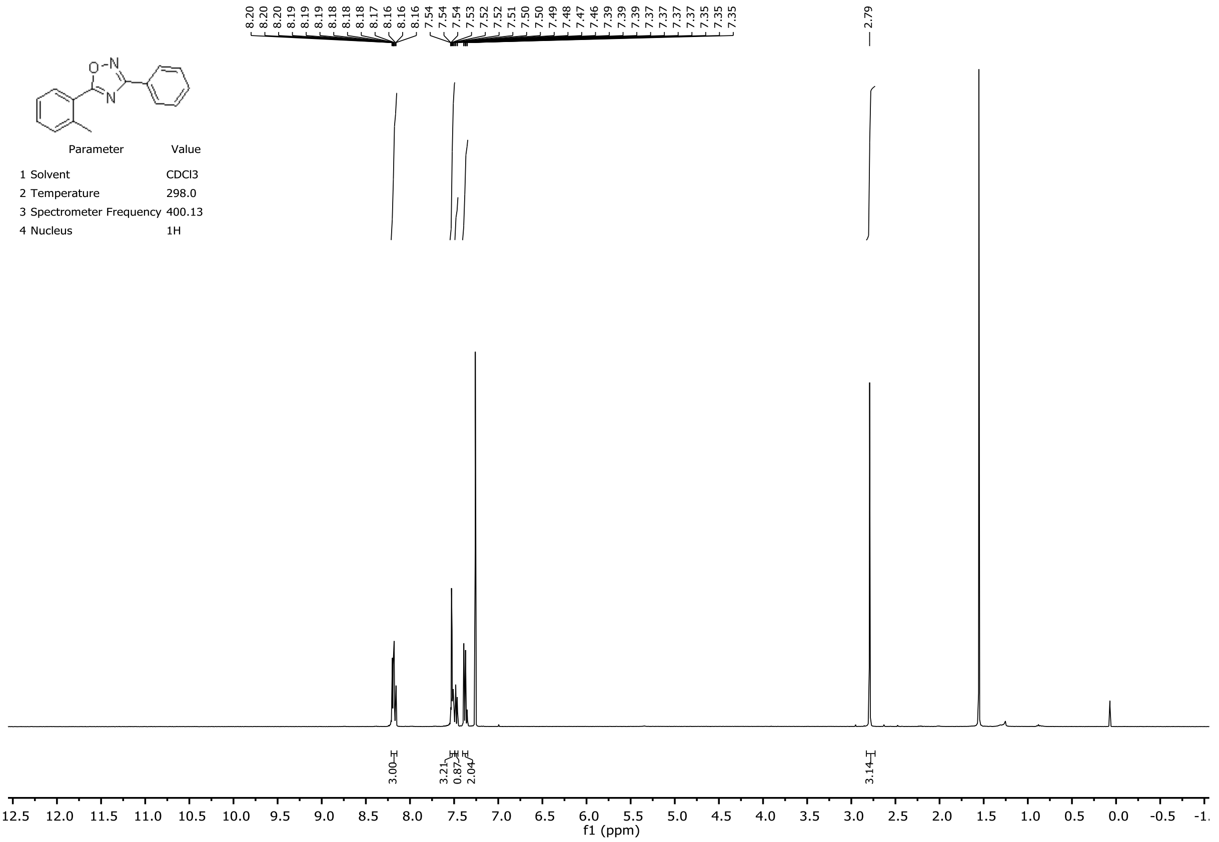

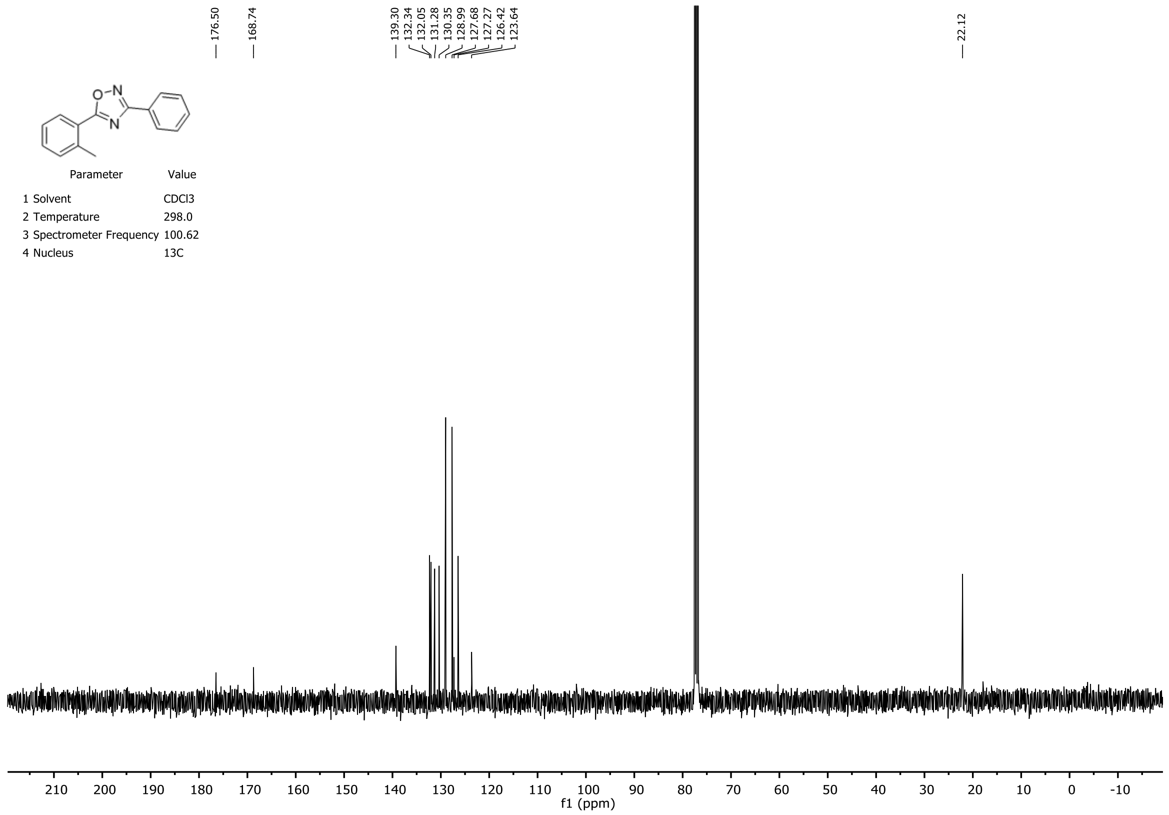

3-phenyl-5-(o-tolyl)-1,2,4-oxadiazole ***6b*** *CAS: 54494-15-4*

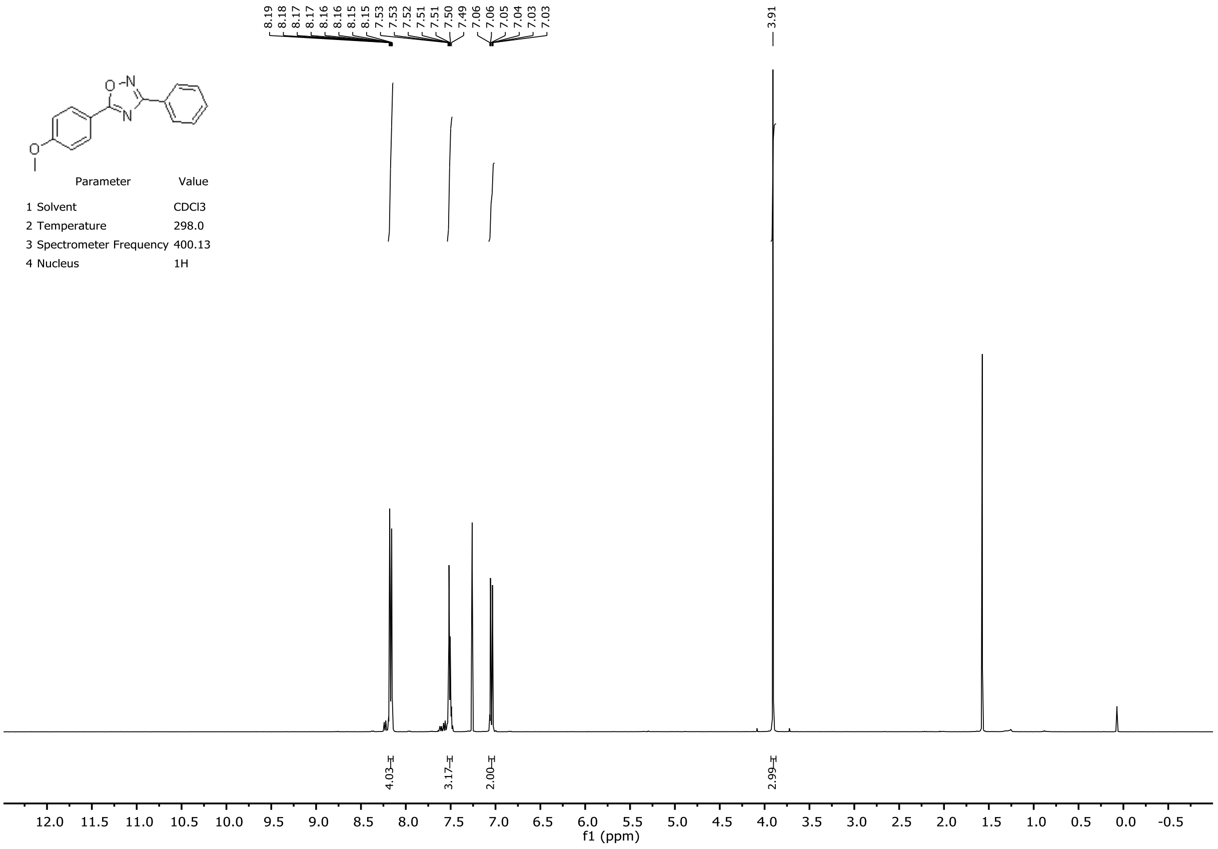

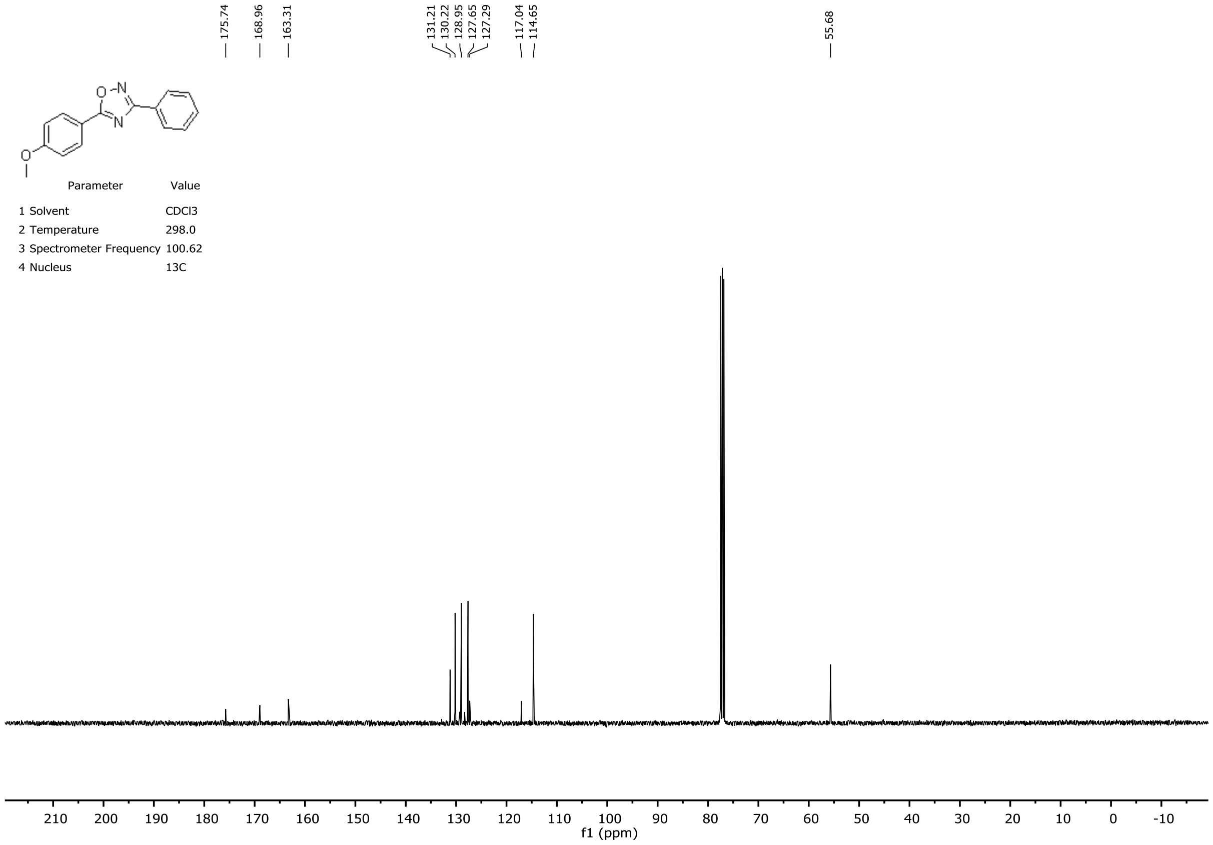

5-(4-methoxyphenyl)-3-phenyl-1,2,4-oxadiazole[1] ***6c*** *CAS: 36364-17-7*

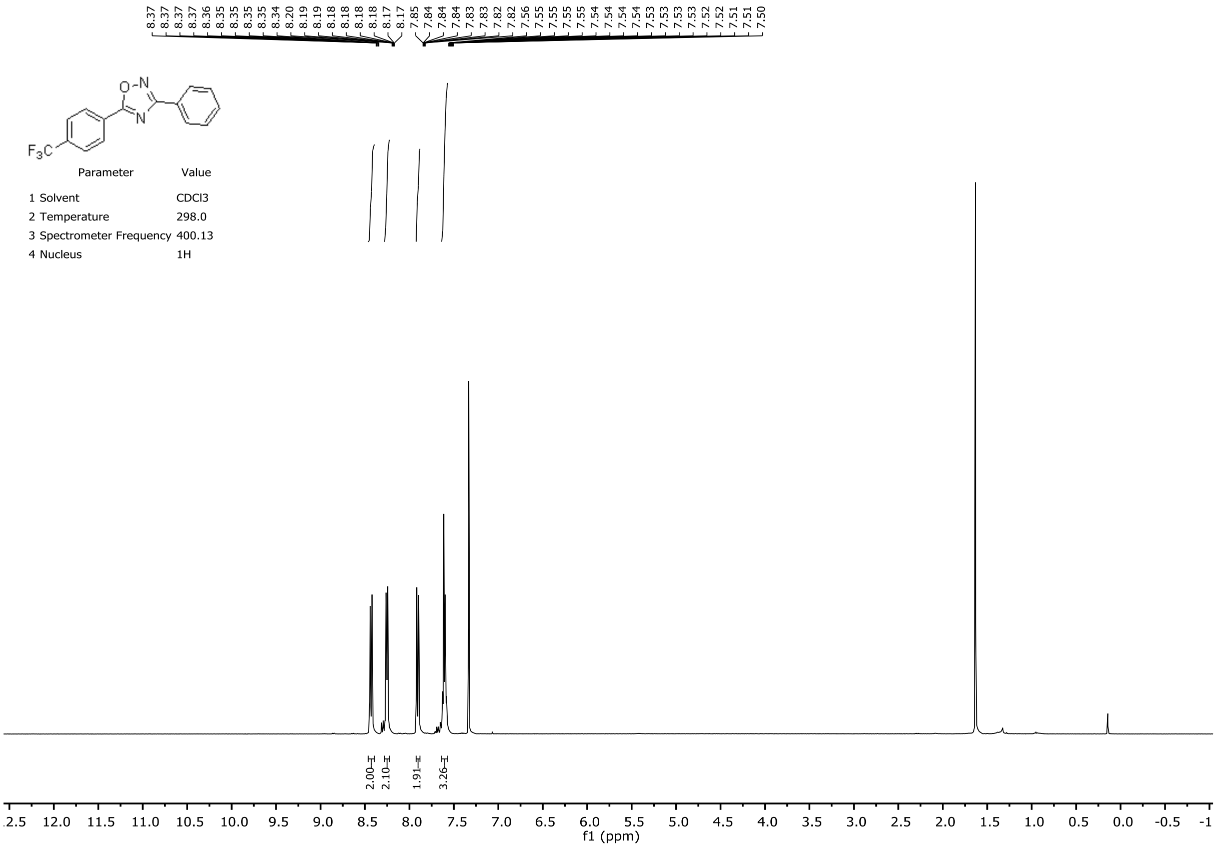

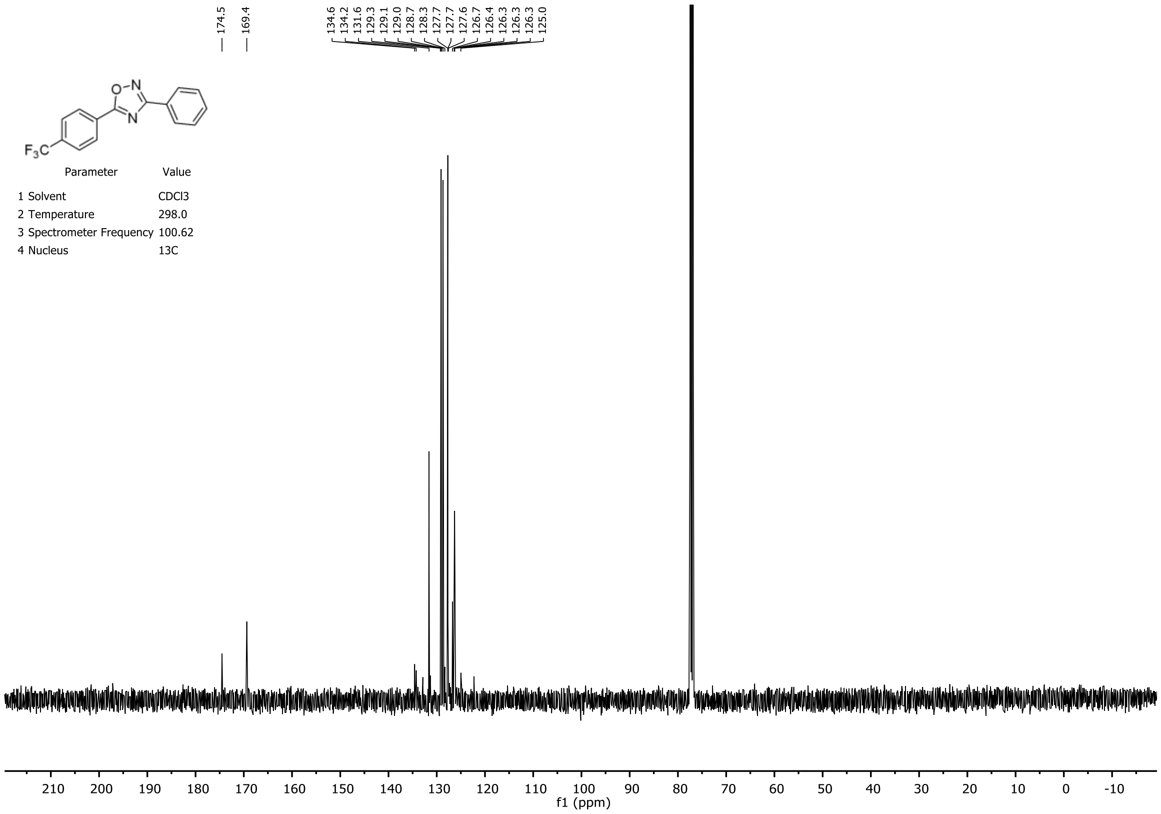

3-phenyl-5-(4-(trifluoromethyl)phenyl)-1,2,4-oxadiazole ***6d*** *CAS: 89804-66-0*

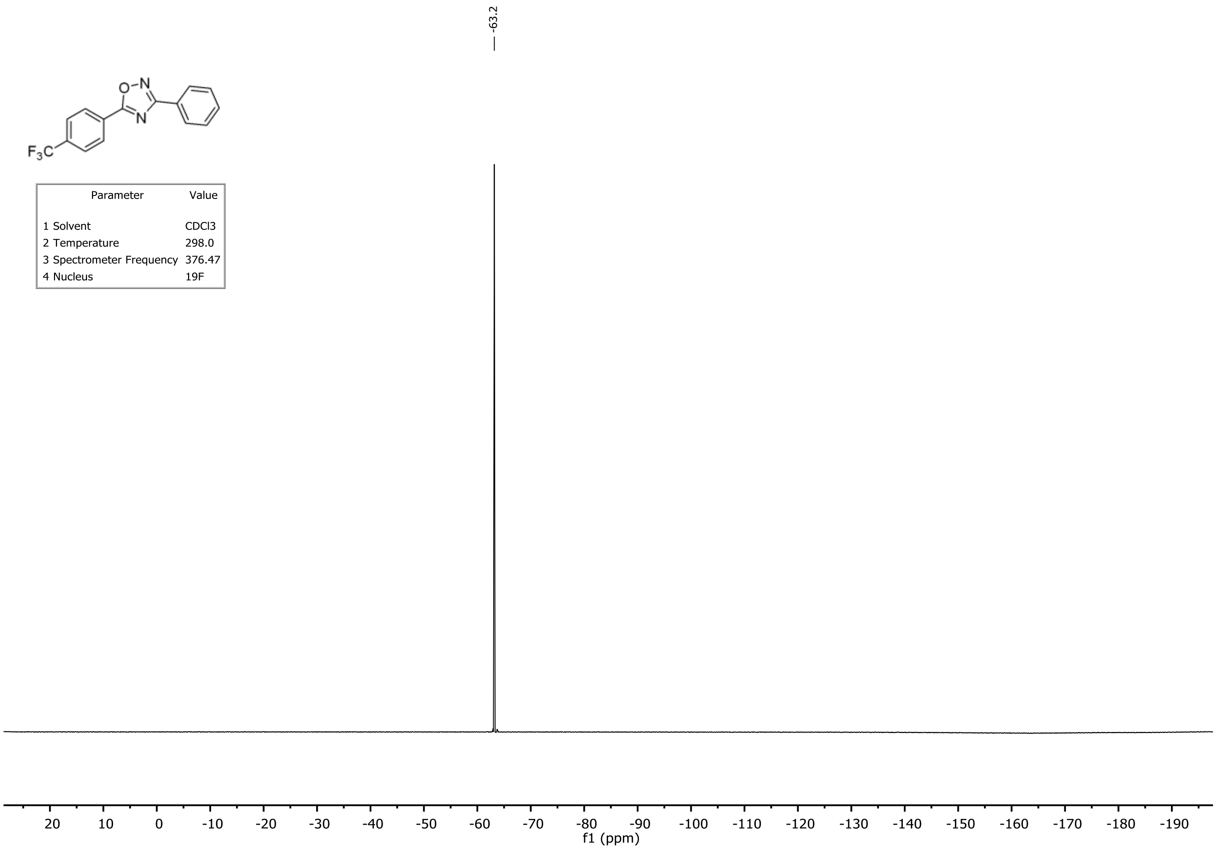

3-phenyl-5-(4-(trifluoromethyl)phenyl)-1,2,4-oxadiazole ***6d*** *CAS: 89804-66-0*

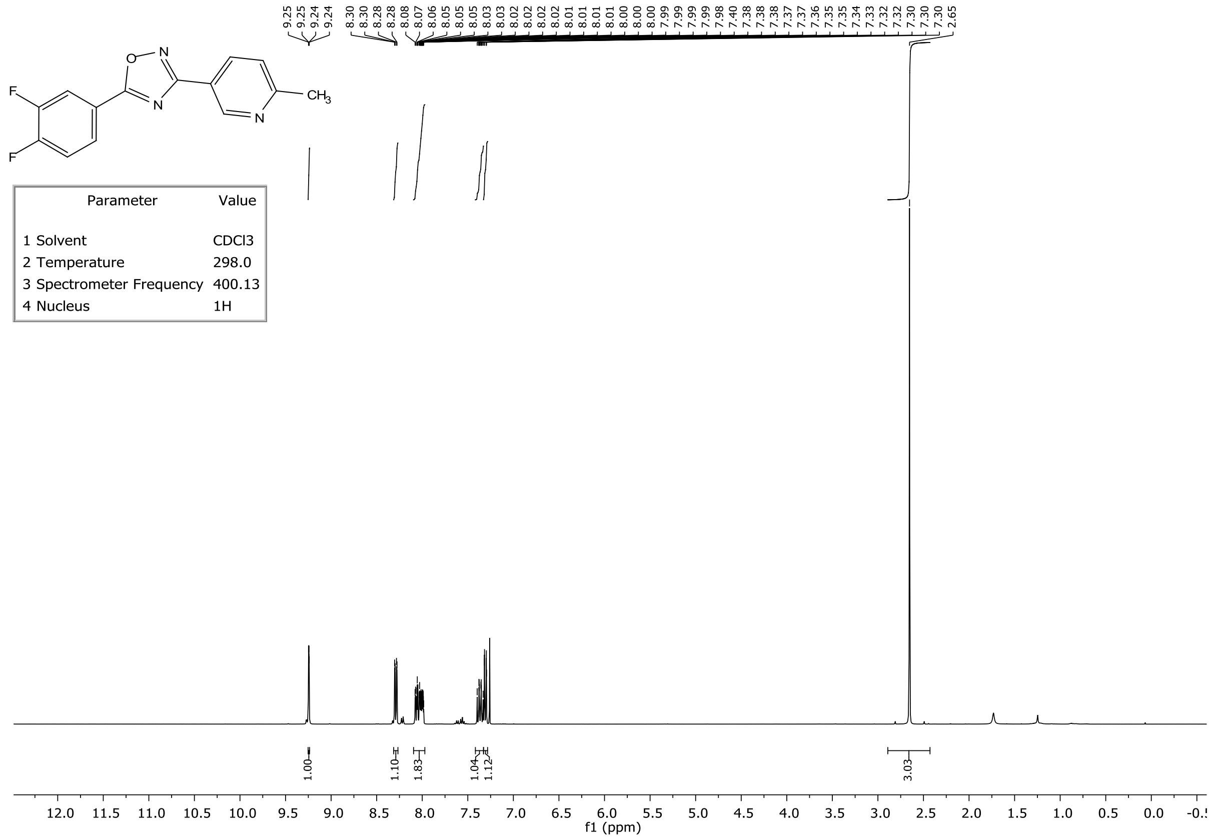

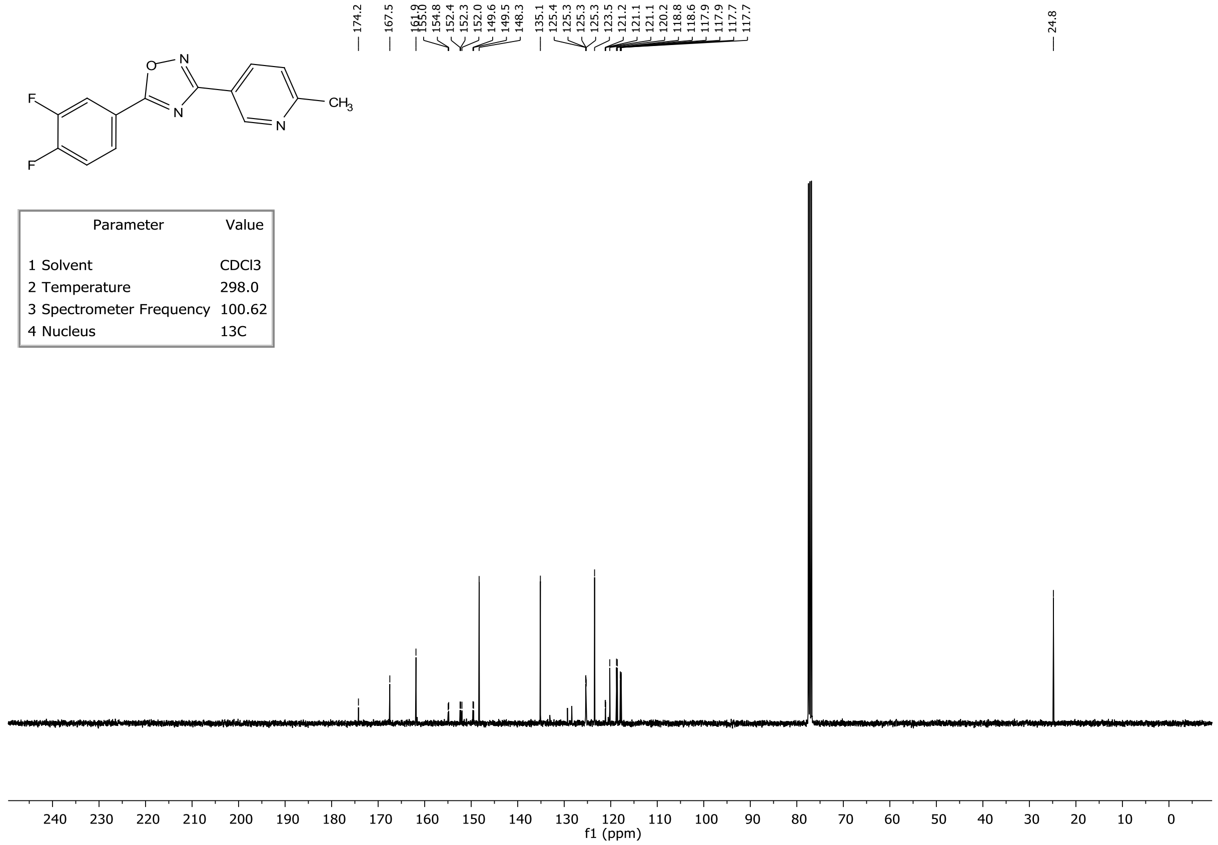

5-(3,4-difluorophenyl)-3-(6-methylpyridin-3-yl)-1,2,4-oxadiazole ***DDO-7263*** *CAS:*

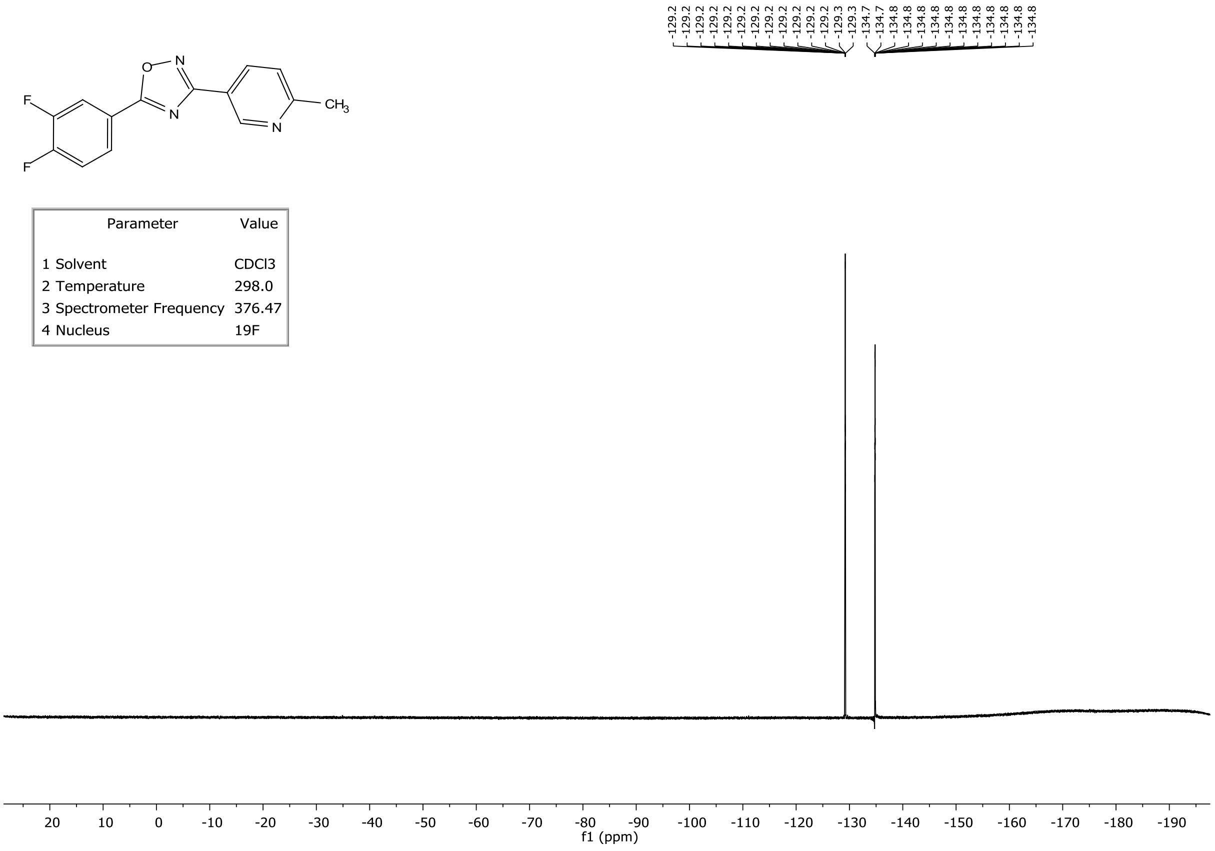

5-(3,4-difluorophenyl)-3-(6-methylpyridin-3-yl)-1,2,4-oxadiazole ***DDO-7263*** *CAS:*

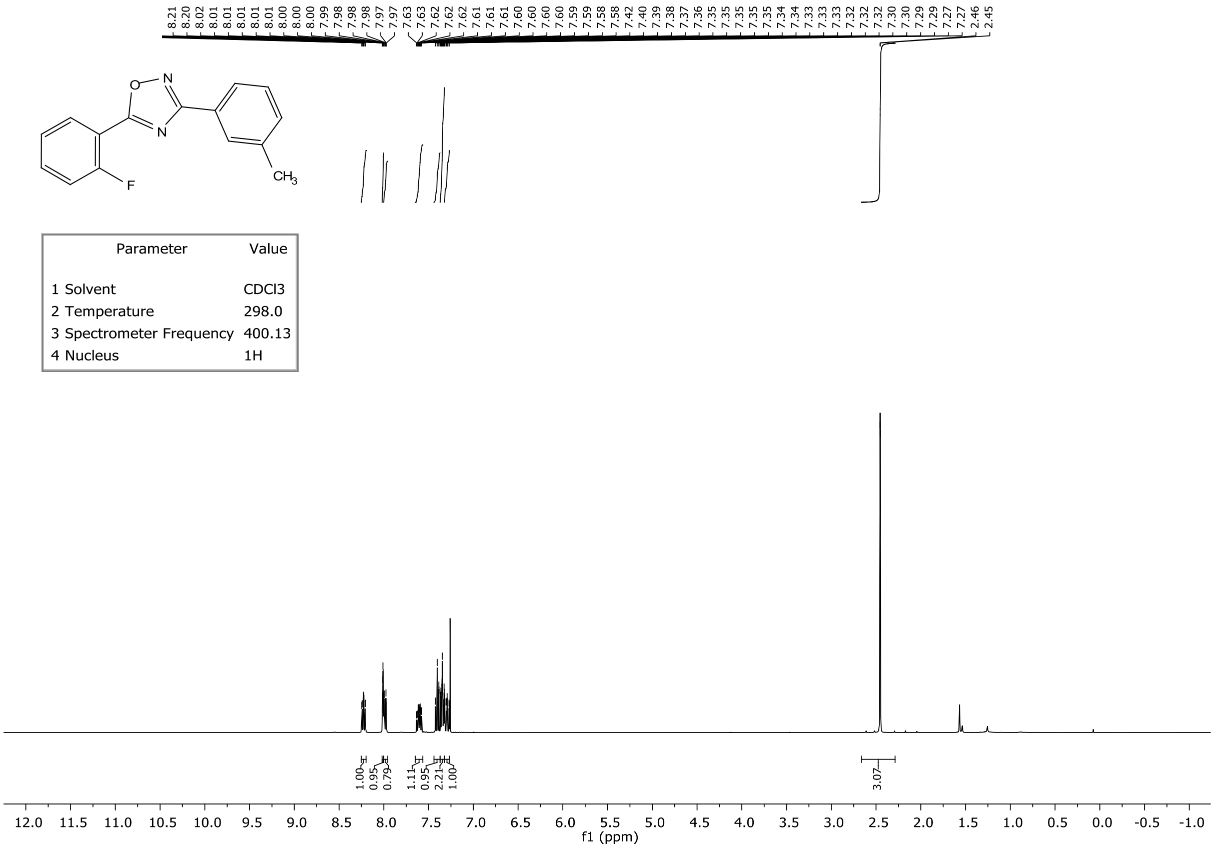

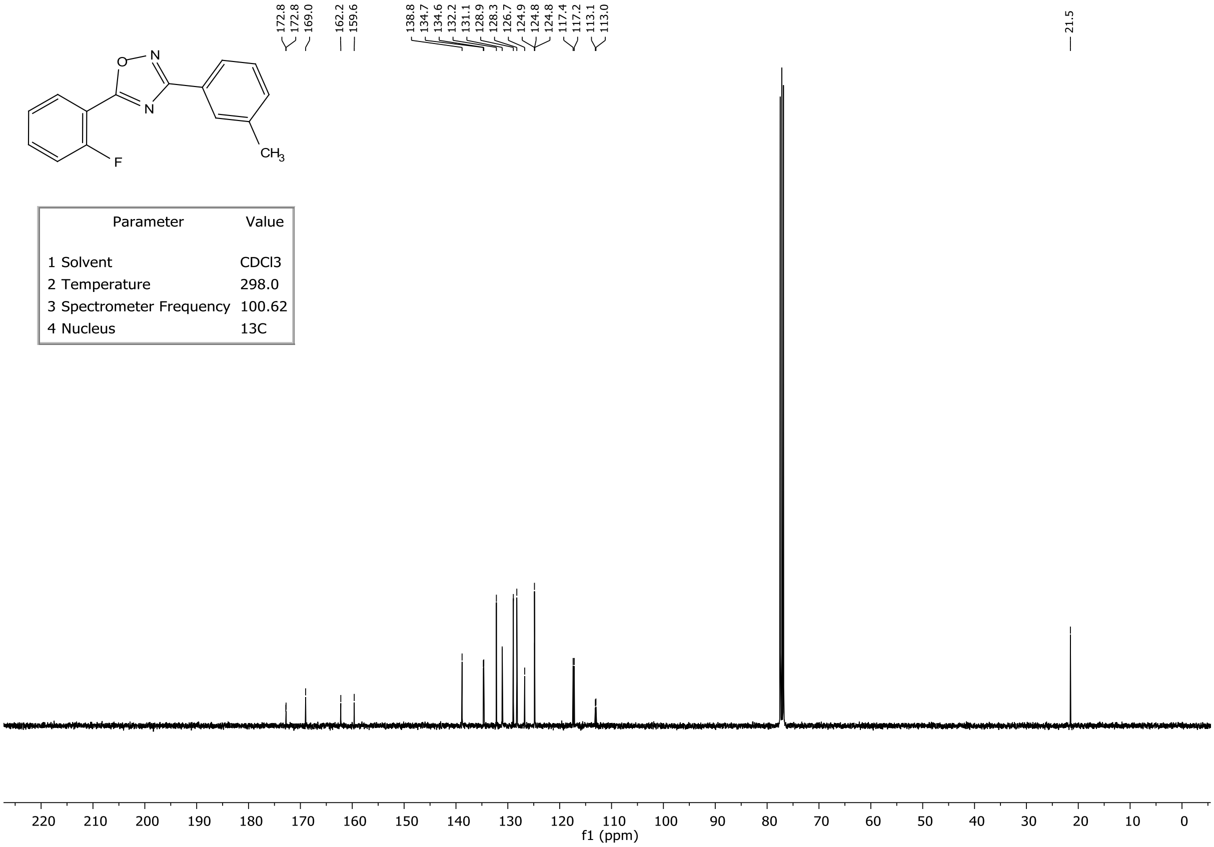

5-(2-fluorophenyl)-3-(m-tolyl)-1,2,4-oxadiazole ***ataluren precursor*** *CAS:*

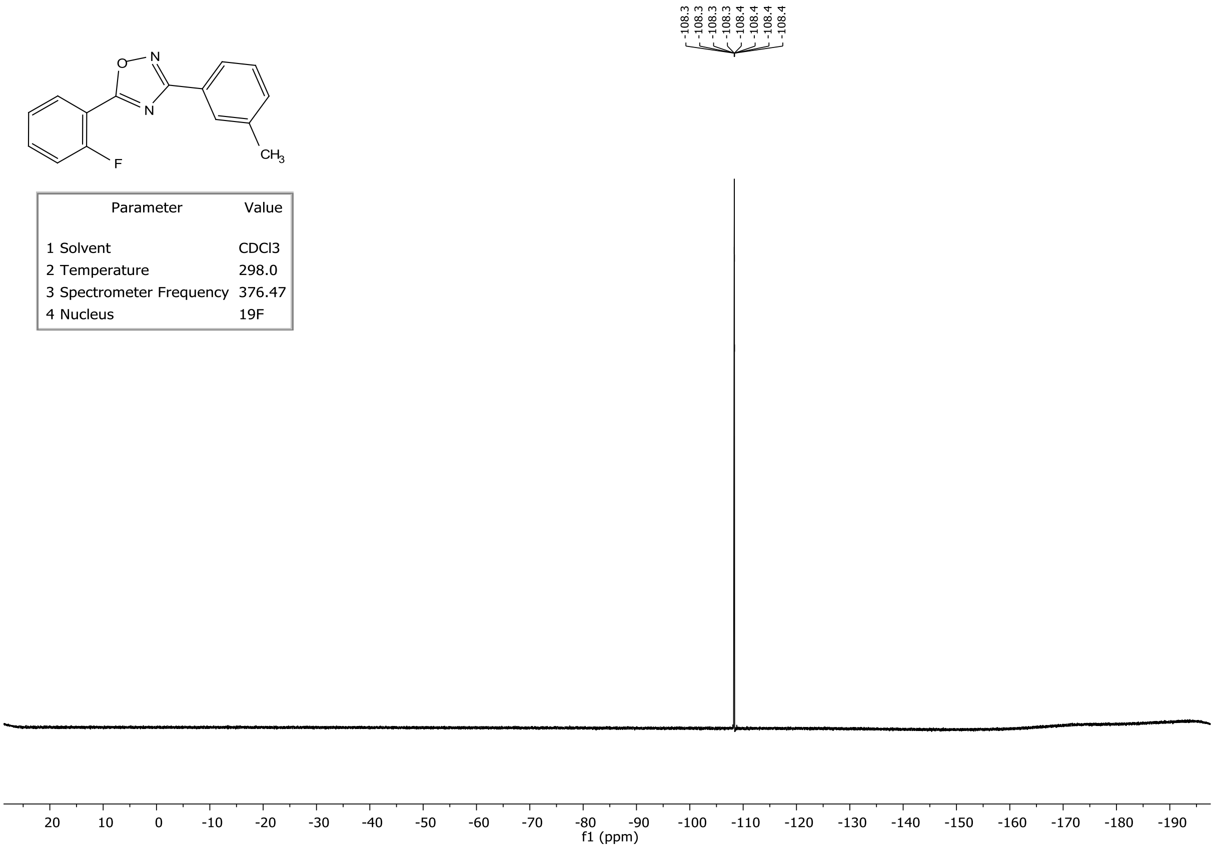

5-(2-fluorophenyl)-3-(m-tolyl)-1,2,4-oxadiazole ***ataluren precursor*** *CAS:*

HPLC-chromatogram of isolated ^{11}C-labeled compounds

[carbonyl-^{11}C]N-(Imino(phenyl)methyl)-4-methoxybenzamide ***^{11}C-3f*** *CAS: 1445133-92-5*

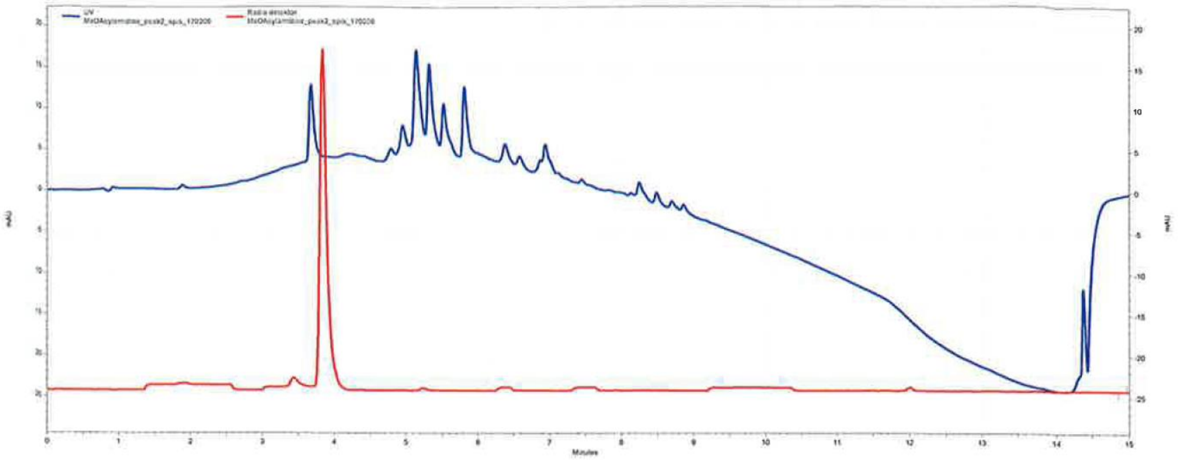

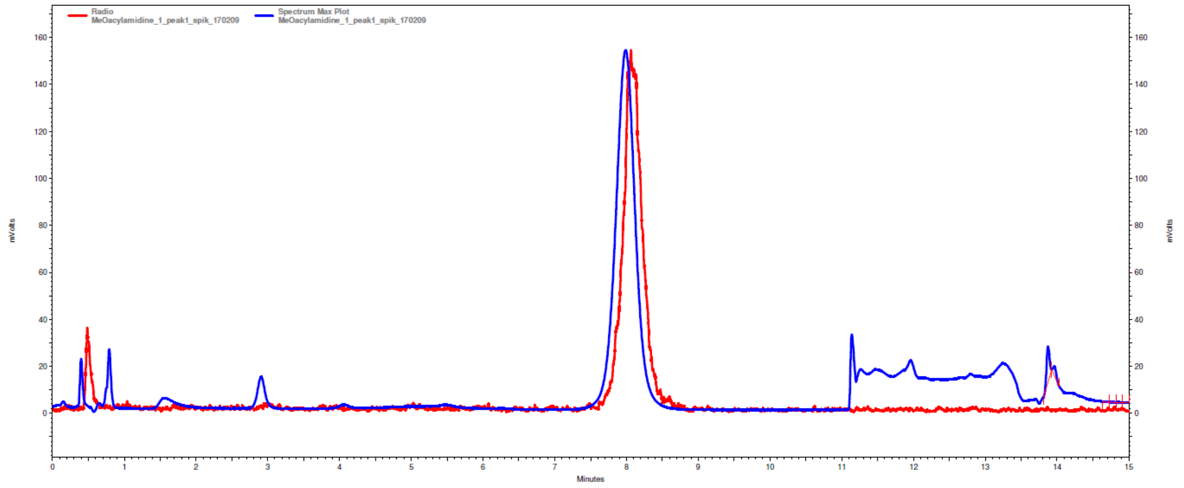

Exp 1. Top, system A; bottom, system B. Red signal = radiodetector, blue signal = UV detector.

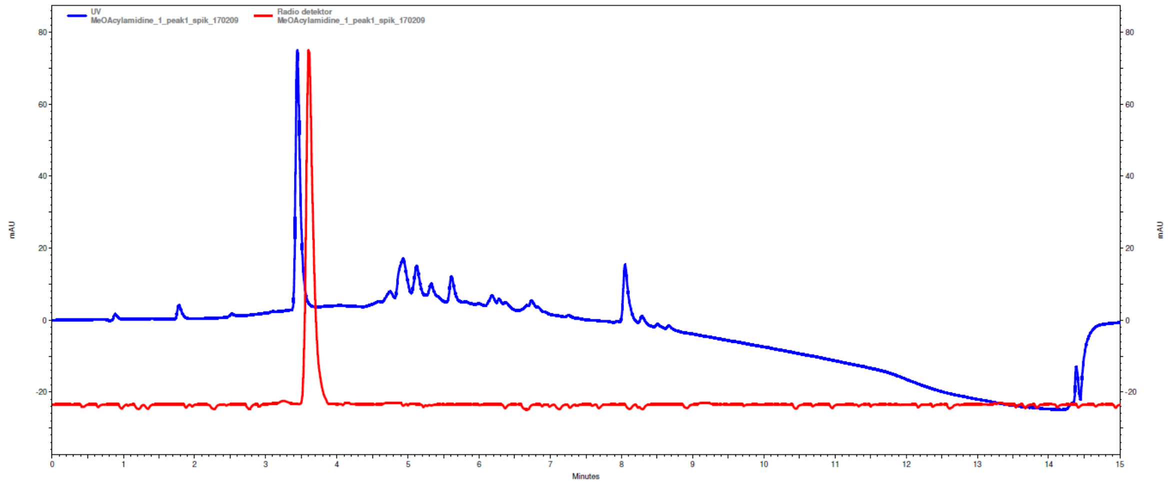

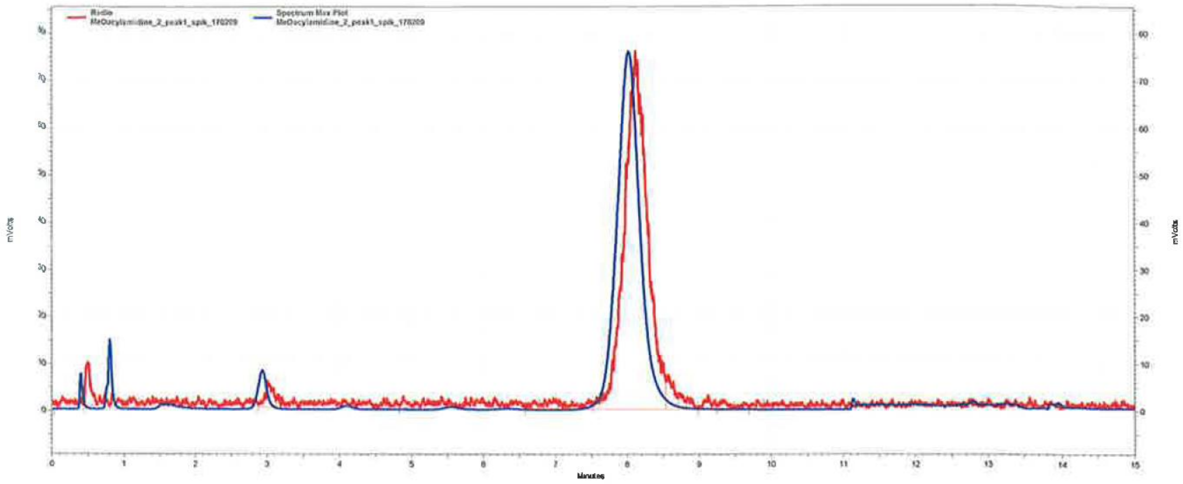

Exp 2. Top, system A; bottom, system B. Red signal = radiodetector, blue signal = UV detector.

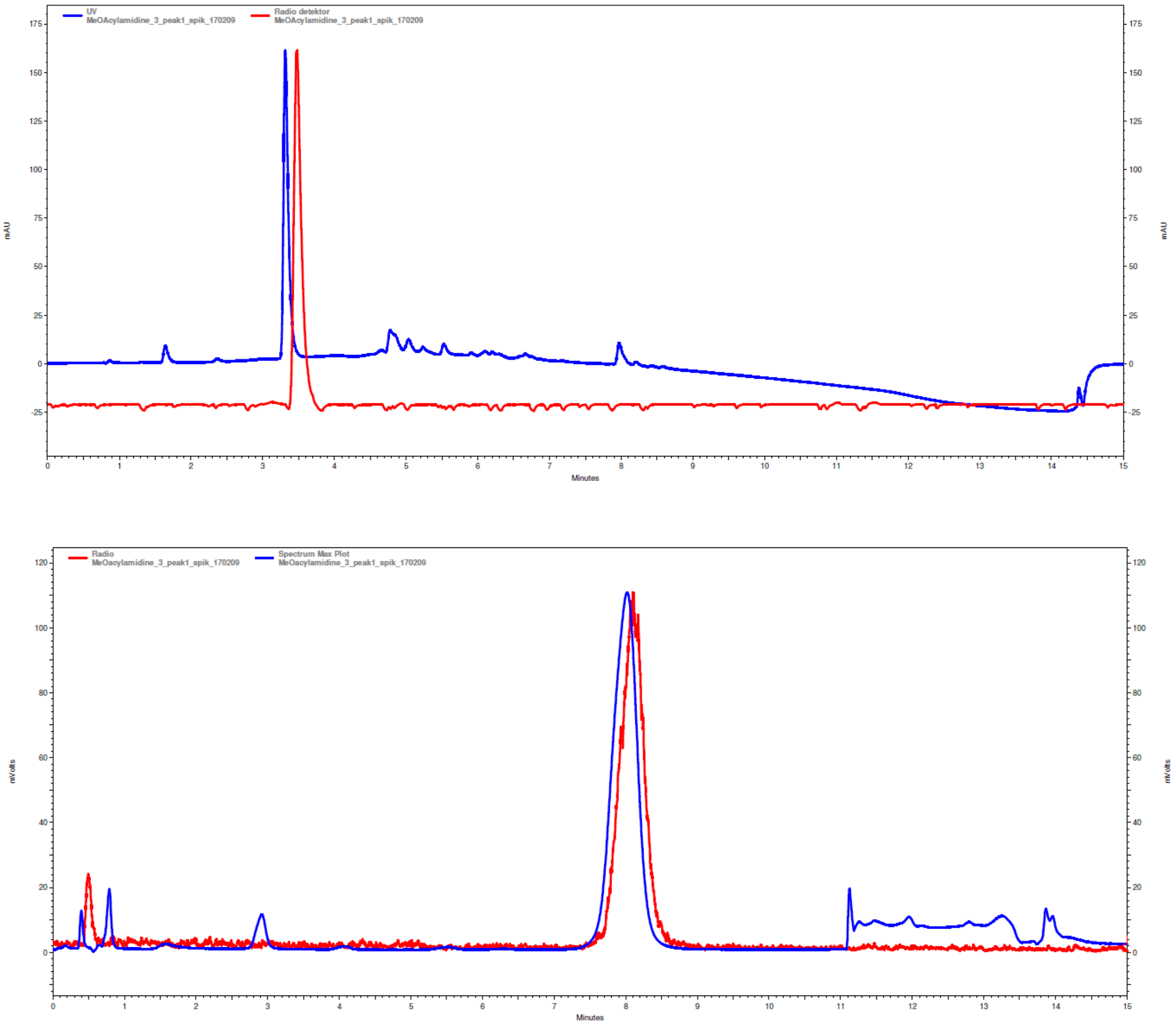

Exp 3. Top, system A; bottom, system B. Red signal = radiodetector, blue signal = UV detector.

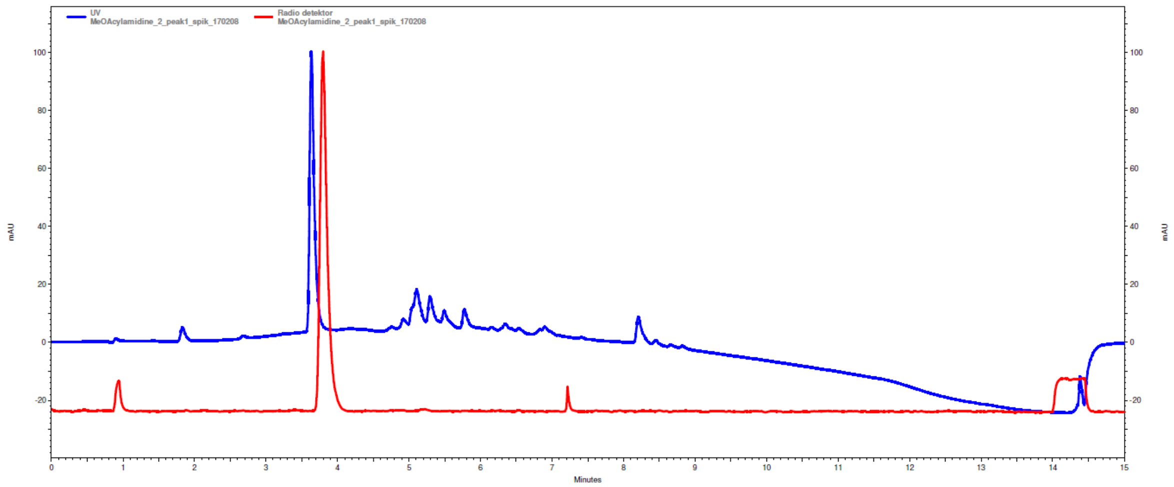

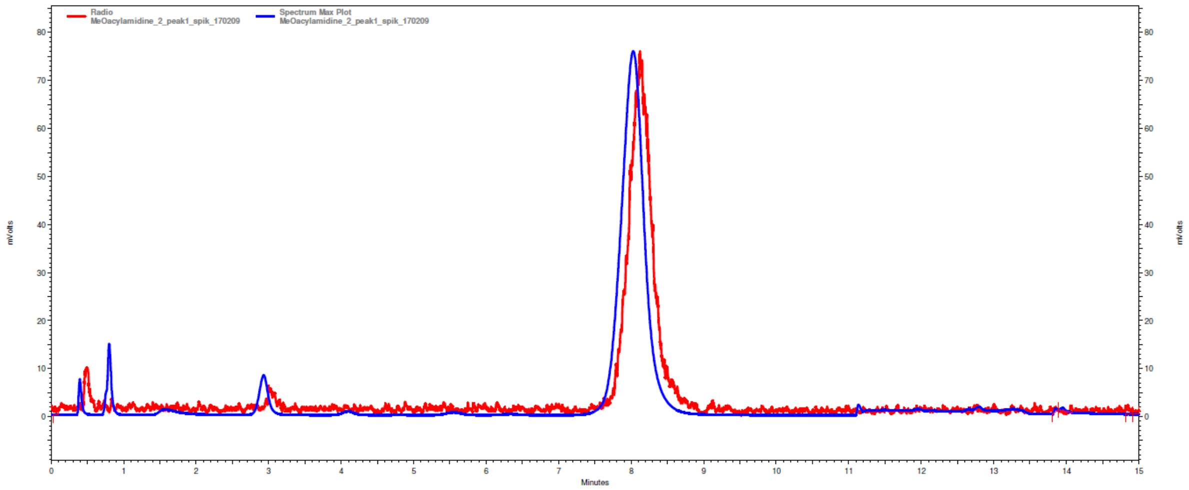

Exp 4. Top, system A; bottom, system B. Red signal = radiodetector, blue signal = UV detector.

[carbonyl-^{11}C]4-Acetyl-N-(imino(phenyl)methyl)benzamide ***[11]C-3b*** *CAS:-*

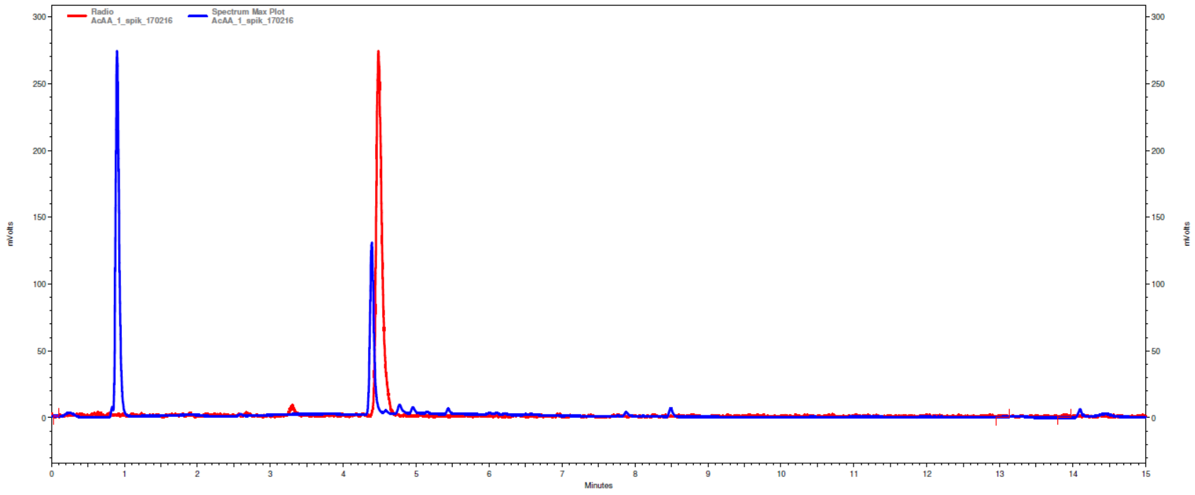

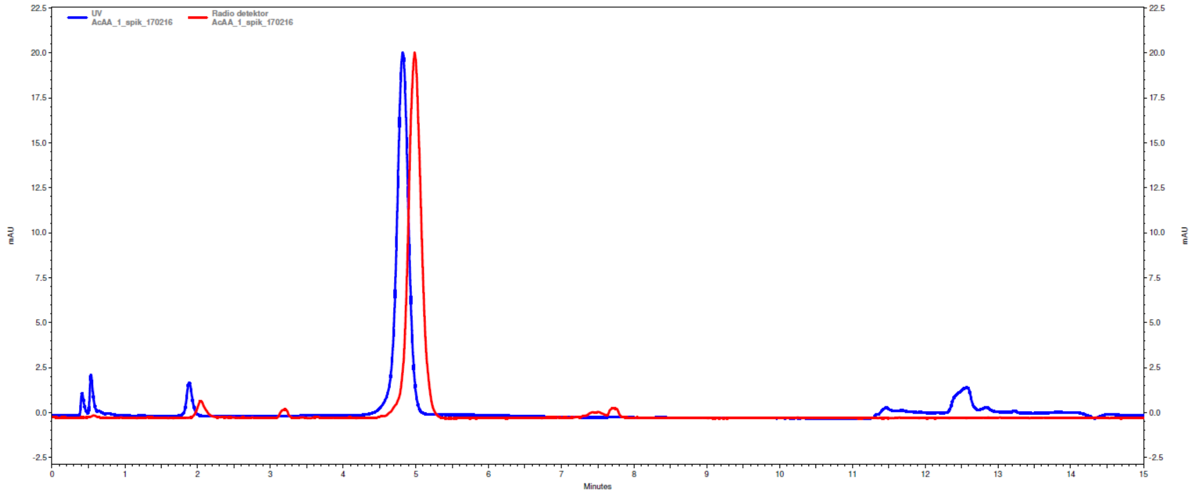

Exp 1. Top, system A; bottom, system B. Red signal = radiodetector, blue signal = UV detector.

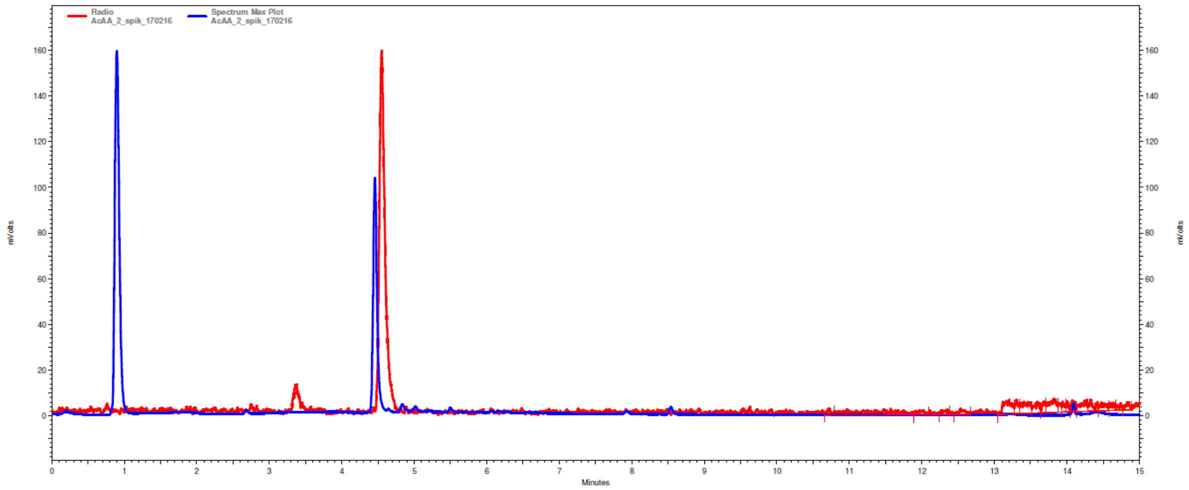

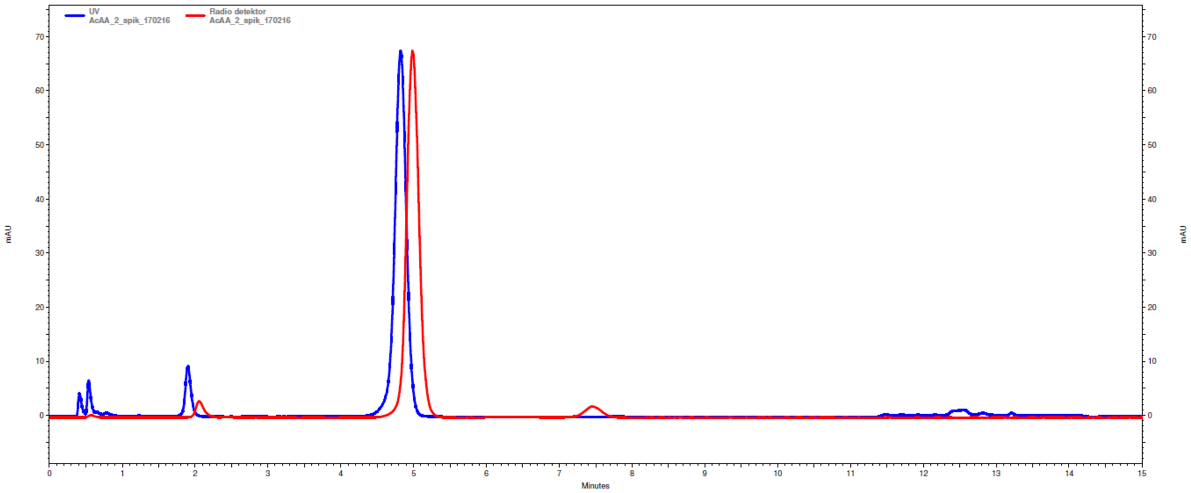

Exp 2. Top, system A; bottom, system B. Red signal = radiodetector, blue signal = UV detector.

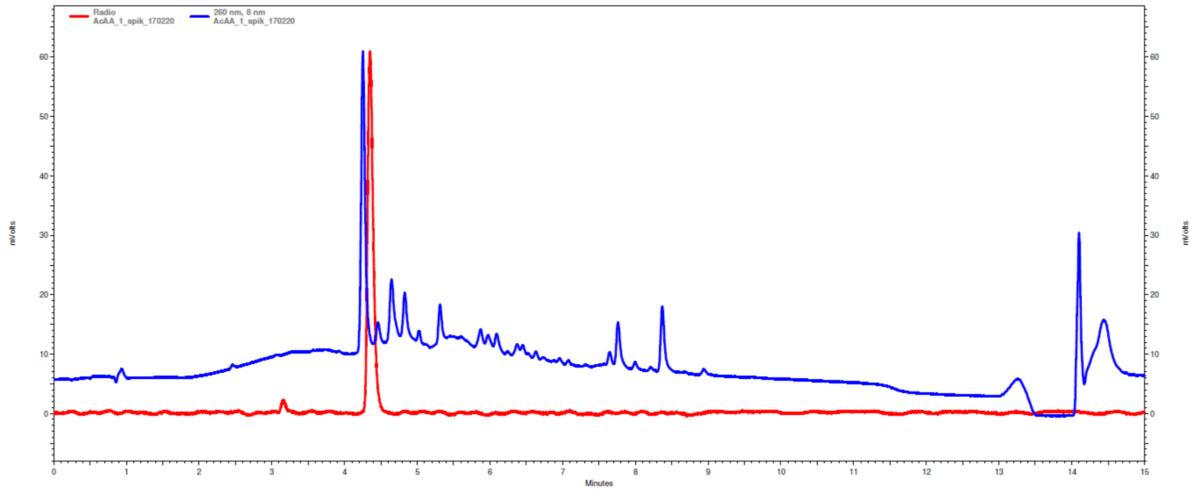

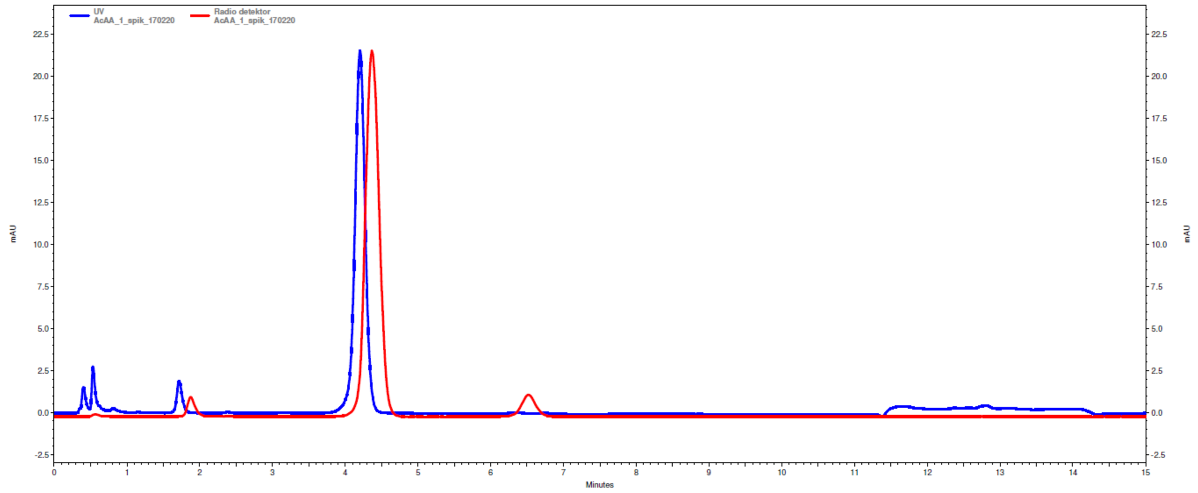

Exp 3. Top, system A; bottom, system B. Red signal = radiodetector, blue signal = UV detector.

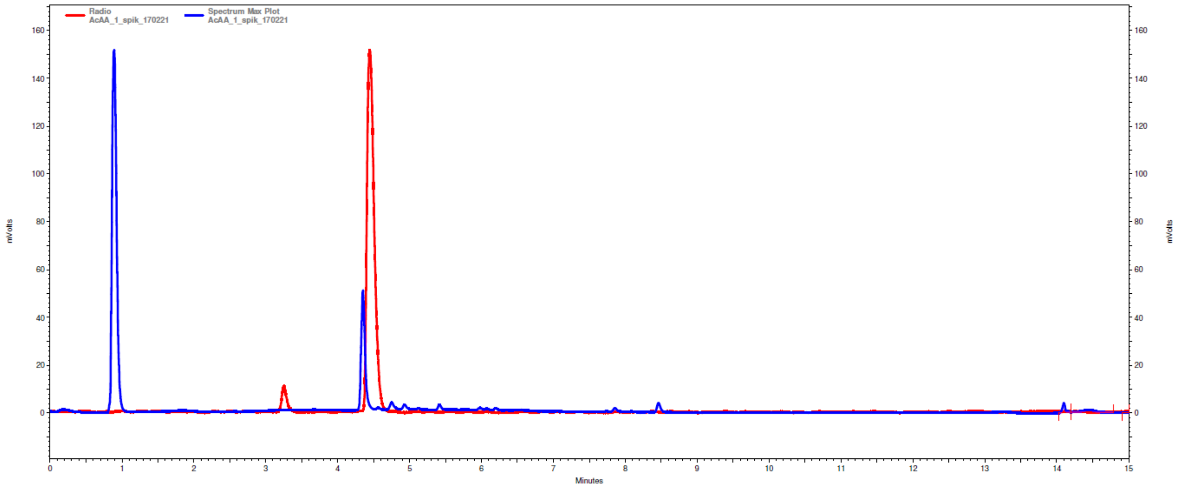

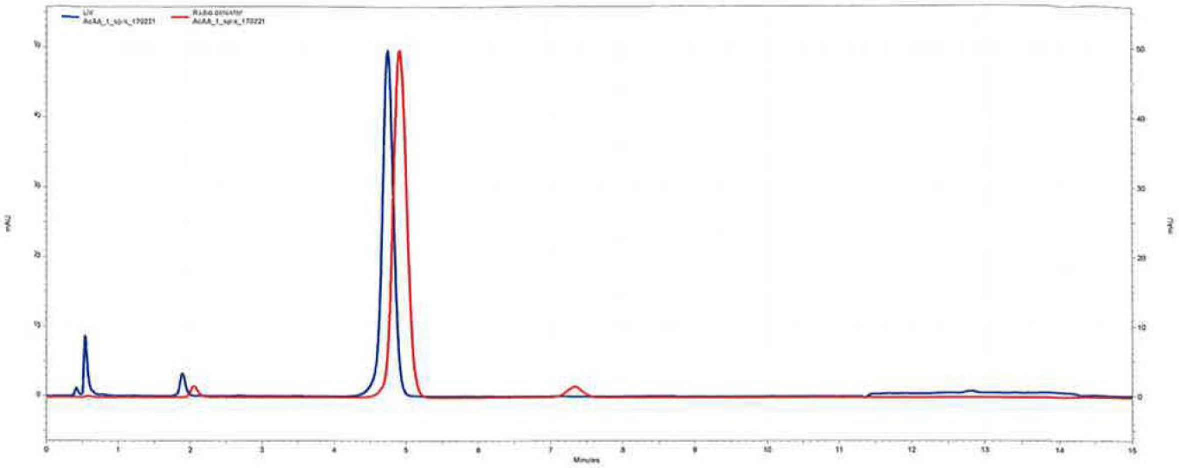

Exp 4. Top, system A; bottom, system B. Red signal = radiodetector, blue signal = UV detector.

[carbonyl-^{11}C]3-Phenyl-5-(p-tolyl)-1,2,4-oxadiazole ^{11}**C-6** *CAS: 16112-24-6*

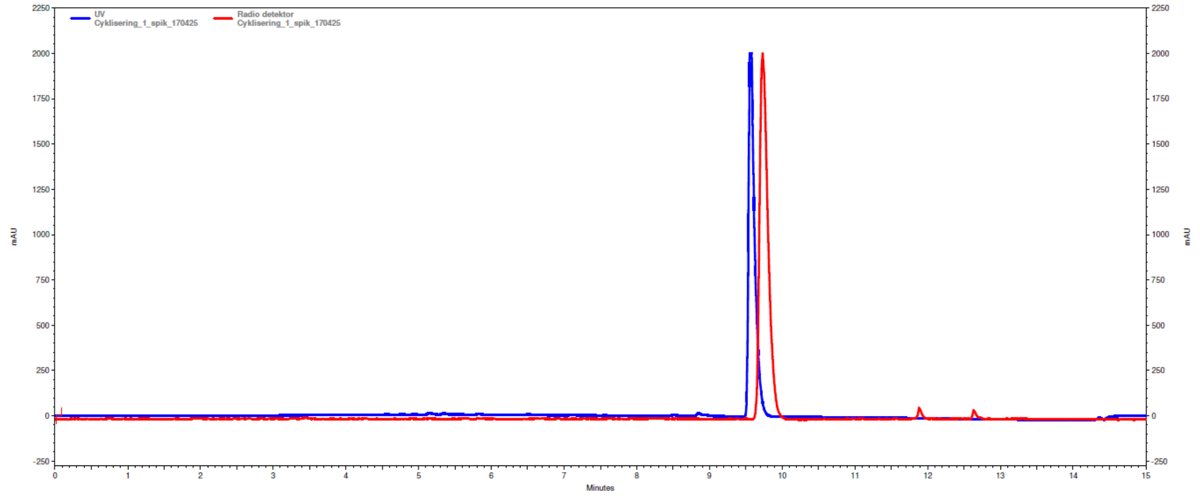

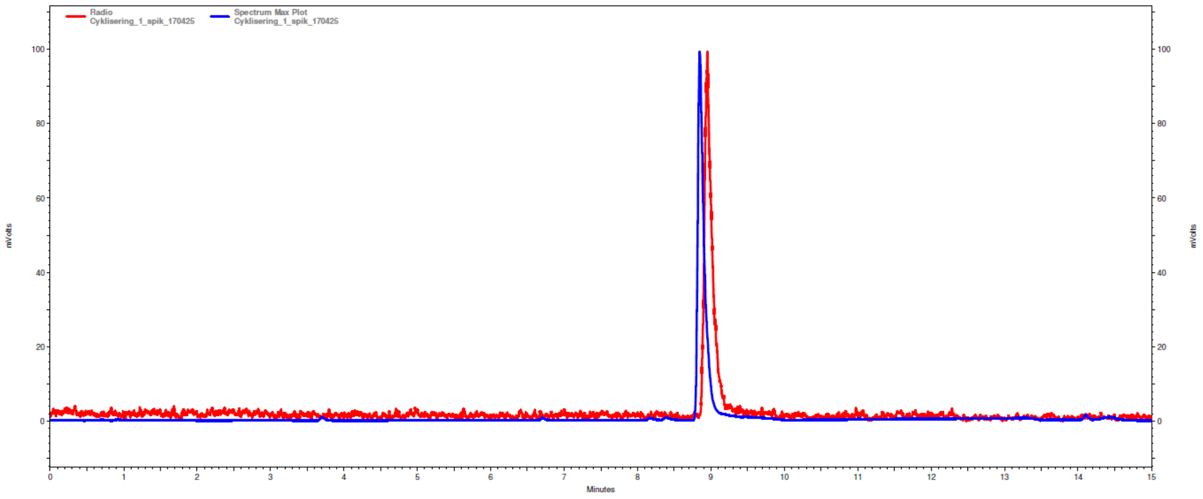

Exp 1. Top, system A; bottom, system B. Red signal = radiodetector, blue signal = UV detector.

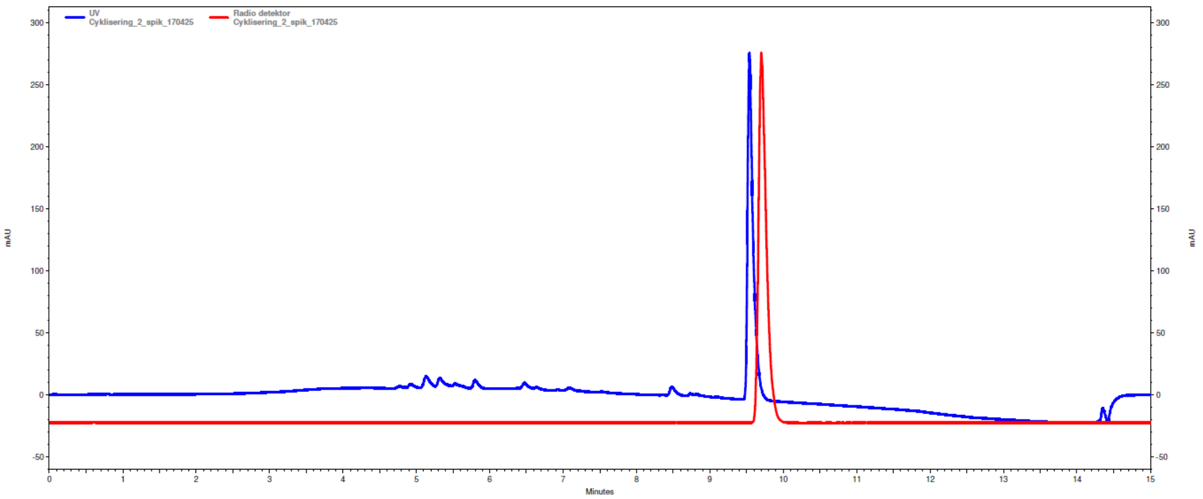

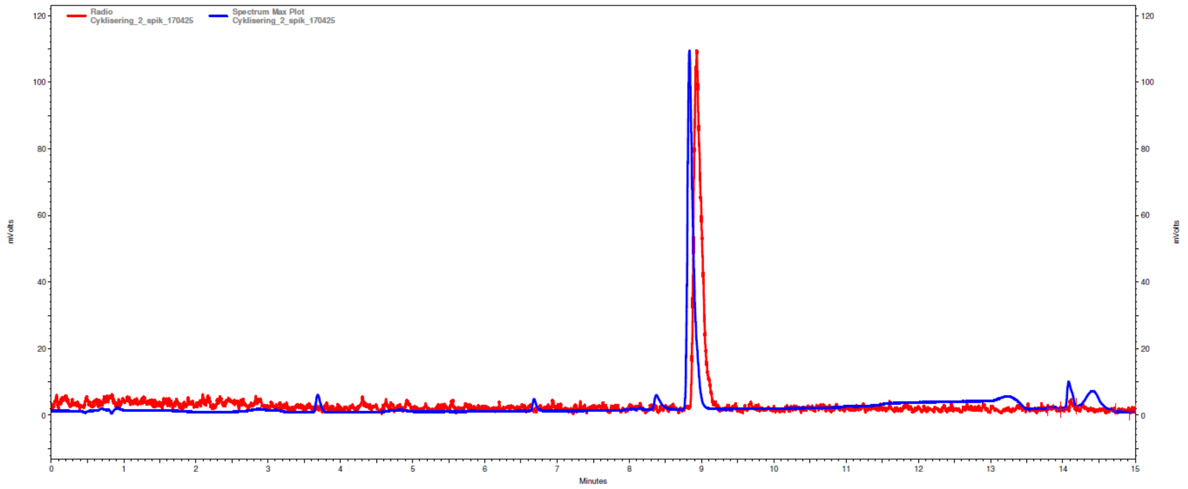

Exp 2. Top, system A; bottom, system B. Red signal = radiodetector, blue signal = UV detector.
